# Dissociation of sodium-chloride cotransporter expression and blood pressure during chronic high dietary potassium supplementation

**DOI:** 10.1172/jci.insight.156437

**Published:** 2023-03-08

**Authors:** Robert Little, Sathish K. Murali, Søren B. Poulsen, Paul R. Grimm, Adrienne Assmus, Lei Cheng, Jessica R. Ivy, Ewout J. Hoorn, Vladimir Matchkov, Paul A. Welling, Robert A. Fenton

**Affiliations:** 1 Department of Biomedicine, Aarhus University, Aarhus, Denmark.; 2 Departments of Medicine, Nephrology and Physiology, Johns Hopkins School of Medicine, Baltimore, USA.; 3 University/BHF Centre for Cardiovascular Science, The Queen’s Medical Research Institute, The University of Edinburgh, Edinburgh, United Kingdom.; 4 Department of Internal Medicine, Erasmus Medical Center, University Medical Center Rotterdam, Rotterdam, The Netherlands.

**Keywords:** Nephrology, Epithelial transport of ions and water, Hypertension, Sodium channels

## Abstract

Dietary potassium (K^+^) supplementation is associated with a lowering effect in blood pressure (BP), but not all studies agree. Here, we examined the effects of short- and long-term K^+^ supplementation on BP in mice, whether differences depend on the accompanying anion or the sodium (Na^+^) intake and molecular alterations in the kidney that may underlie BP changes. Relative to the control diet, BP was higher in mice fed a high NaCl (1.57% Na^+^) diet for 7 weeks or fed a K^+^-free diet for 2 weeks. BP was highest on a K^+^-free/high NaCl diet. Commensurate with increased abundance and phosphorylation of the thiazide sensitive sodium-chloride-cotransporter (NCC) on the K^+^-free/high NaCl diet, BP returned to normal with thiazides. Three weeks of a high K^+^ diet (5% K^+^) increased BP (predominantly during the night) independently of dietary Na^+^ or anion intake. Conversely, 4 days of KCl feeding reduced BP. Both feeding periods resulted in lower NCC levels but in increased levels of cleaved (active) α and γ subunits of the epithelial Na^+^ channel ENaC. The elevated BP after chronic K^+^ feeding was reduced by amiloride but not thiazide. Our results suggest that dietary K^+^ has an optimal threshold where it may be most effective for cardiovascular health.

## Graphical abstract

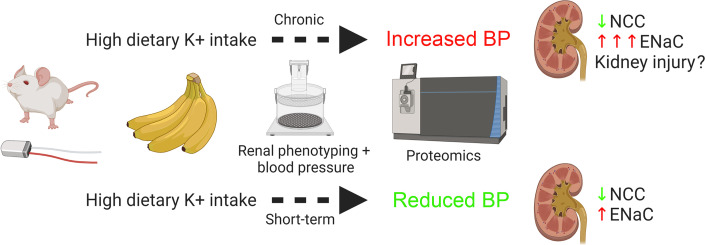

## Introduction

Chronically elevated blood pressure (BP), or hypertension, is a major cause of preventable mortality worldwide (1). Despite application of various BP-lowering strategies, many patients do not respond with an effective lowering of BP, in line with current guidelines (1). Hence, there remains a need to define molecular mechanisms underpinning hypertension with an aim to develop and progress novel therapeutic strategies.

Diet has a strong influence on BP. Sustained high dietary Na^+^ consumption is associated with increased BP, with epidemiological and clinical reports presenting a positive, independent linear relationship between 24-hour Na^+^ excretion and BP (2–4). The effects of dietary potassium (K^+^) on BP are more controversial. Globally, dietary K^+^ intake is 1.5- to 2-fold lower than recommended (2–4). Increasing dietary K^+^ to recommended intake can reduce BP in hypertensive patients (5, 6). Conversely, nonhypertensive subjects kept on a constant Na^+^ diet demonstrate an average systolic BP (SBP) increase of 10 mmHg when subjected to dietary K^+^ depletion (7). Furthermore, the Dietary Approaches to Stop Hypertension (DASH) regime of dietary Na^+^ restriction alongside increased K^+^ intake causes significant reductions in BP (8), and urinary K^+^ excretion negatively correlates with BP (4, 9–11). Notably, a recent study demonstrated that, in older patients with hypertension, substituting 25% of dietary NaCl intake with KCl lowered SBP by ~3.5 mmHg and significantly reduced the rates of major cardiovascular events (12). Whether this is attributable to the higher K^+^ intake, lower Na^+^ intake, or a combination is unclear. Contrasting these studies, urinary K^+^ excretion ≥ 1 g/day, or a decrease in the urinary Na^+^/K^+^ ratio, was not associated with lower SBP in a reanalysis of the DASH trial data set obtained during the initial period where participants consumed their regular diet (13). Furthermore, a “U-shaped” association between K^+^ intake and BP was uncovered in a meta-analysis of randomized-controlled trials (duration ≥4 weeks), with SBP increasing when dietary intake was under 30 mmol/day or over 80 mmol/day (14).

The kidney is essential for K^+^ homeostasis. Almost all filtered K^+^ is reabsorbed in proximal segments, whereas K^+^ secretion in the distal segments matches urinary K^+^ excretion with dietary K^+^ intake. The activity of the thiazide-sensitive sodium-chloride-cotransporter (NCC) in the distal convoluted tubule (DCT) is important for controlling the delivery of Na^+^ to the downstream K^+^ secreting segment: the aldosterone-sensitive distal nephron (ASDN), where K^+^ secretion by the renal outer medullary K^+^ channel (ROMK) channel is electrochemically coupled to Na^+^ reabsorption via the epithelial Na^+^ channel ENaC (15). Aldosterone-regulated flow-dependent K^+^ secretion can also occur via big-K^+^ channels (“maxi-K” channels) (16, 17). The importance of Na^+^ delivery for K^+^ secretion is highlighted by disorders of Na^+^ reabsorption in the DCT. Urinary K^+^ wasting and hypokalemia develops in patients with Gitelman syndrome, as loss of NCC function causes excessive Na^+^ delivery to the ASDN. In contrast, urinary K^+^ retention and hyperkalemia are hallmarks of patients with pseudohypoaldosteronism type II (PHAII) who have hyperactivation of NCC (18, 19).

ENaC and NCC abundance and/or activity are altered by dietary K^+^. ENaC expression and cleavage (indicating higher activity) increase with high dietary K^+^ (20, 21) and decrease with K^+^ restriction (22). Conversely, NCC abundance and phosphorylation (indicating higher activity) often increase when dietary K^+^ intake is restricted (20, 22–24) but generally decrease subsequent to high dietary K^+^ (20, 24–26). The effects on NCC may be dependent on the period of dietary K^+^ intervention or the anion accompanying dietary K^+^ (27–29).

In an attempt to address the variable BP differences observed in humans and mice after K^+^ supplementation, and to better align the period of dietary intervention in mice with human studies, here we (a) determined if short-term (4 days) or chronic (21 days) K^+^ supplementation to mice alters BP; (b) assessed if any effects of chronic K^+^ supplementation on BP are dependent on the accompanying anion or the Na^+^ intake; and (c) determined if specific molecular alterations in the kidney underlie BP differences. Our results show that a K^+^-free diet increases BP in an NCC-dependent manner — an effect augmented by a high NaCl diet. Independent of the Na^+^ intake or accompanying anion, chronic K^+^ supplementation also increases BP, whereas short-term high KCl (+KCl) feeding reduces BP. These differential effects on BP occur despite a reduction in NCC abundance and phosphorylation under both dietary K^+^ conditions. The chronic feeding effects on BP may be driven by enhanced ENaC activity, but kidney damage may also play a role.

## Results

### Low dietary K^+^ intake increases BP.

**Figure 1:**
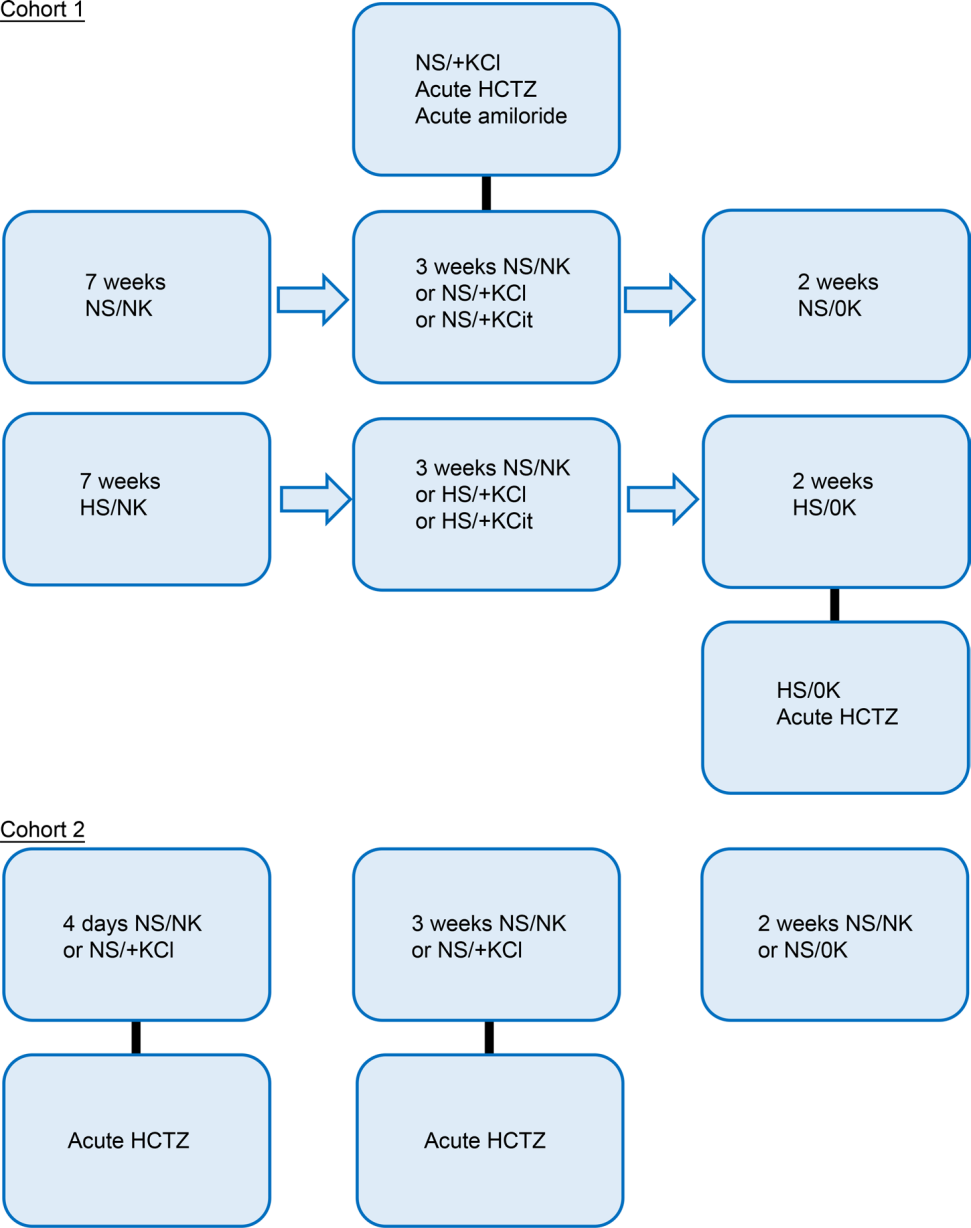
Project outline. In the initial cohort (cohort 1), mice were subjected to a chronic dietary regime with blood pressure determined at each dietary stage. Diet key for all figures: NS, normal NaCl (0.3% Na^+^); HS, high NaCl (1.57% Na^+^); NK, normal KCl (1.05% K^+^); +KCl, high KCl (5.25% K^+^); +KCit, high K citrate (5.25% K^+^); 0K, zero K^+^ diet. HCTZ, hydrochlorothiazide. In cohort 2, mice were subjected to either short-term or chronic dietary regime, with blood pressure determined at each period.

**Figure 2:**
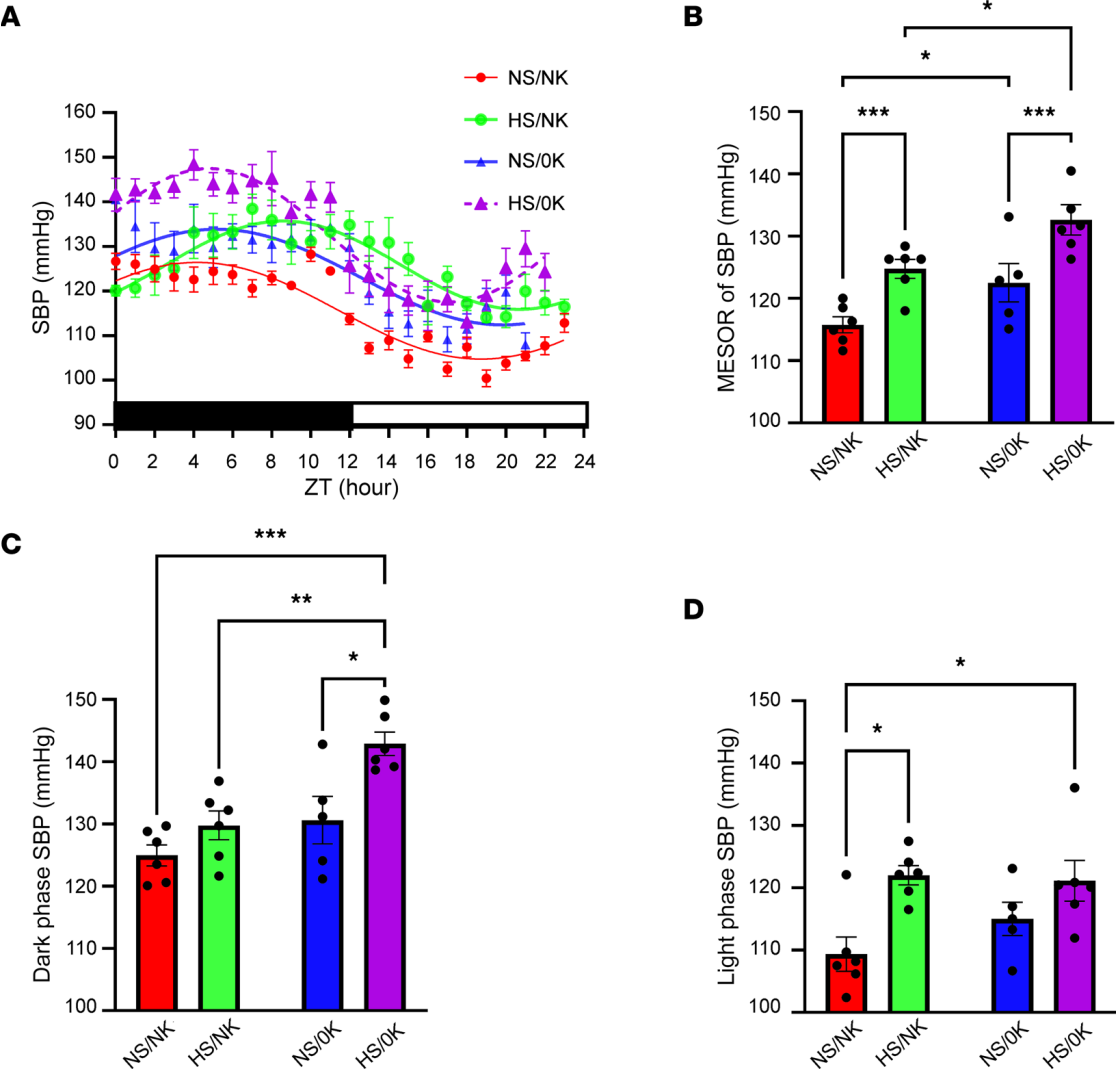
Low dietary K^+^ intake increases BP. Systolic blood pressure (SBP) was recorded by in vivo radio telemetry from individual free-roaming animals. (**A**) Comparison of fits analysis of curves showed a significant difference (*P* < 0.0001) across a 24-hour period (indicating significantly higher SBP) between animals fed high-NaCl (HS/NK) diet compared with animals fed control NaCl (NS/NK) diet. A significant difference (*P* < 0.0001) in curves was also observed after 2-week feeding of a K^+^-deplete diet (NS/0K), with a high-NaCl, K^+^-deplete diet (HS/0K) having the largest difference in SBP relative to control (NS/NK) diet (*P* < 0.0001). Data are shown as mean ± SEM, *n* = 5–6 per dietary condition. Dark/light times are shown by lower bar strip. ZT hour 0 = 18:00 hours. (**B**–**D**) Quantification of the MESOR value of SBP (representing the SBP averaged across a 24-hour period), 12-hour SBP when animals are in darkness (active phase), and 12-hour light phase. Data are shown as mean ± SEM with individual values shown. Data was analyzed using 2-way ANOVA with Tukey’s multiple-comparison testing. *P < 0.05; **P < 0.01; ***P < 0.001.

In cohort 1, mice were subjected to a chronic dietary regime alongside measurements of BP (Figure 1). Mice maintained on a control diet (normal Na^+^ normal K^+^ [NS/NK]) exhibited a circadian pattern of BP (Supplemental Figure 1; supplemental material available online with this article; https://doi.org/10.1172/jci.insight.156437DS1). After feeding a high Na^+^/normal K^+^ diet (HS/NK) for 7 weeks, mice exhibited a significantly increased SBP compared with NS/NK-fed mice (Figure 2A), with the Midline Estimate of Rhythmicity (MESOR) value (representing the mean SBP across 24 hours) significantly elevated (Figure 2B). After feeding a K^+^-deplete diet (0K) for 2 weeks combined with normal Na^+^ (NS/0K) or high Na^+^ (HS/0K), SBP was significantly elevated over 24 hours compared with NS/NK-fed animals (Figure 2A), with a tendency for the effects to be higher during darkness when phosphorylated NCC (pNCC) levels may be higher (30). Assessment by MESOR showed similar changes (Figure 2B). SBP was significantly higher for HS/0K-fed animals compared with NS/0K-fed animals over 24 hours and by MESOR value (Figure 2B). Although HS/NK feeding increased SBP during the dark period (Figure 2C), effects were greater during the light period (Figure 2D). Augmentation of SBP following HS/0K feeding occurred predominantly during the dark phase (Figure 2, C and D). Changes of SBP were mirrored by changes in diastolic BP (DBP) and mean arterial pressure (MAP) (Supplemental Table 1). Pulse pressure (PP) was not significantly different between the diets (Supplemental Table 1), suggesting that SBP and DBP have a similar proportional effect following dietary changes.

**Figure 3:**
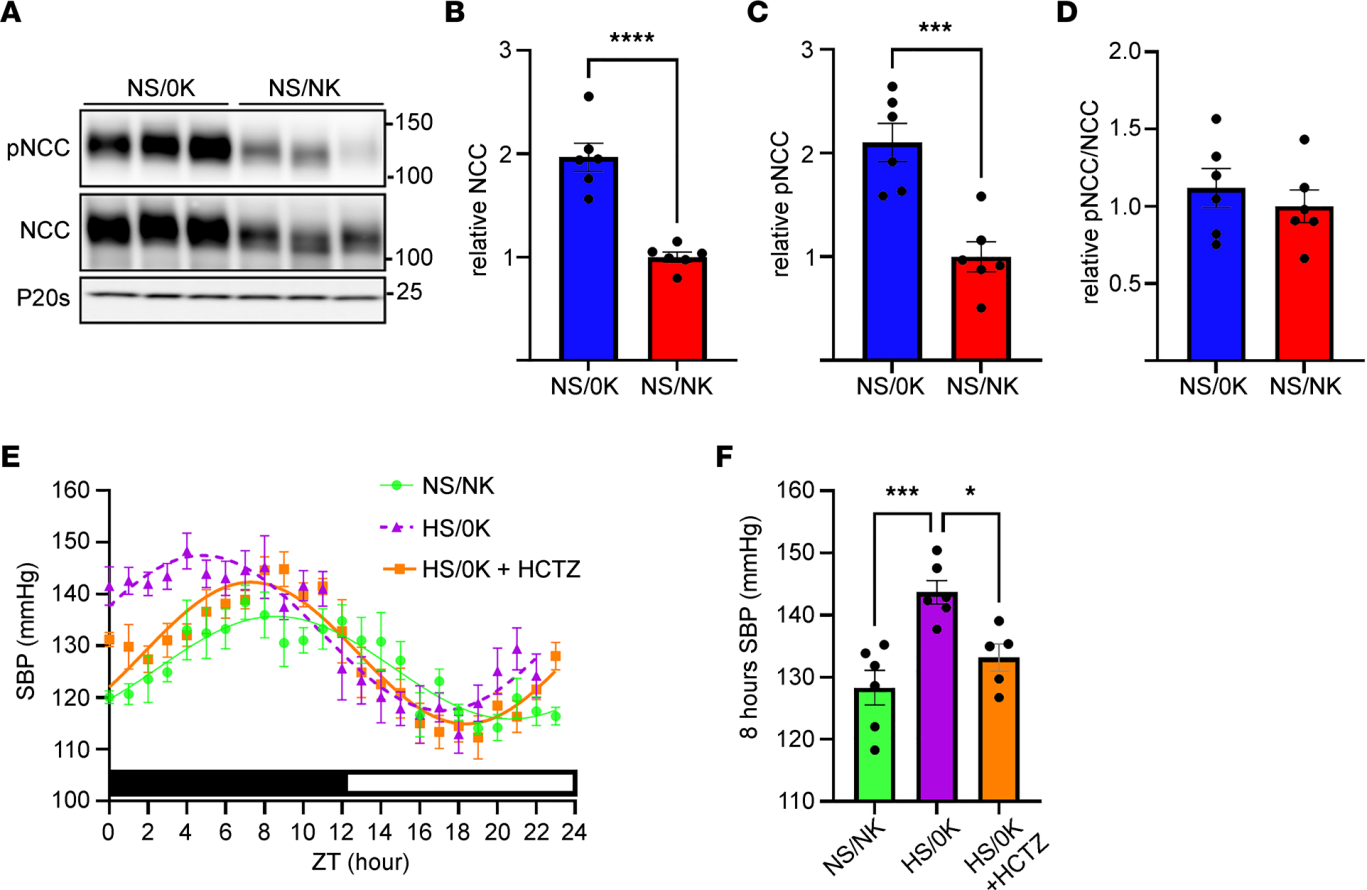
NCC is increased following low dietary K^+^ feeding and is responsible for the increase in BP. Lysates of total kidney from animals fed control diet (NS/NK) or K^+^-deplete diet (NS/0K) for 2 weeks were assessed by western blotting. (**A**) Representative immunoblots of total NCC, phosphorylated NCC (pNCC), or proteasome 20s (P20s, loading control). Molecular weight (KDa) is shown on the right. (**B** and **C**) NCC and pNCC are significantly increased by the NS/0K diet. (**D**) Ratio of pNCC/NCC is not significantly different between NS/0K and NS/NK-fed animals. Data are shown as mean ± SEM with individual values shown. ***P < 0.001 by 2-tailed *t* test. (**E**) Comparison of fits analysis of curves showed a significant difference (*P* < 0.0001) in SBP across a 24-hour period between animals fed a high-NaCl, K^+^-deficient (HS/0K) diet compared with animals fed high-NaCl, normal K^+^ (HS/NK) diet. Over a 24-hour period, curves were significantly different (*P* < 0.0001) between hydrochlorothiazide-treated (HCTZ-treated) animals on a HS/0K diet compared with HS/0K diet alone. Dark/light times are shown by lower bar strip. Time 0 = 18:00 hours. Data are shown as mean ± SEM, *n* = 5–6 per condition. (**F**) HCTZ significantly reduces SBP averaged over 8 hours following injection. Data are shown as mean ± SEM with individual values shown. *P < 0.05; ***P < 0.001 by 1-way ANOVA with Dunnett’s multiple-comparison test.

Elevation of SBP by dietary K^+^ depletion was paralleled by an increase in NCC and pNCC, suggesting higher NCC activity (Figure 3, A–D; all full-length noncropped blots for all figures are shown in Supplemental Data File 1). The NCC inhibitor hydrochlorothiazide (HCTZ) (31) significantly lowered the SBP of HS/0K-fed animals across 24 hours (Figure 3E). In line with its half-life (32), the effects of HCTZ were greatest over the 8 hours following the injection (Figure 3F). Vehicle injection had no significant effect on SBP (Supplemental Figure 2).

### Chronic high K^+^ feeding elevates BP.

**Figure 4:**
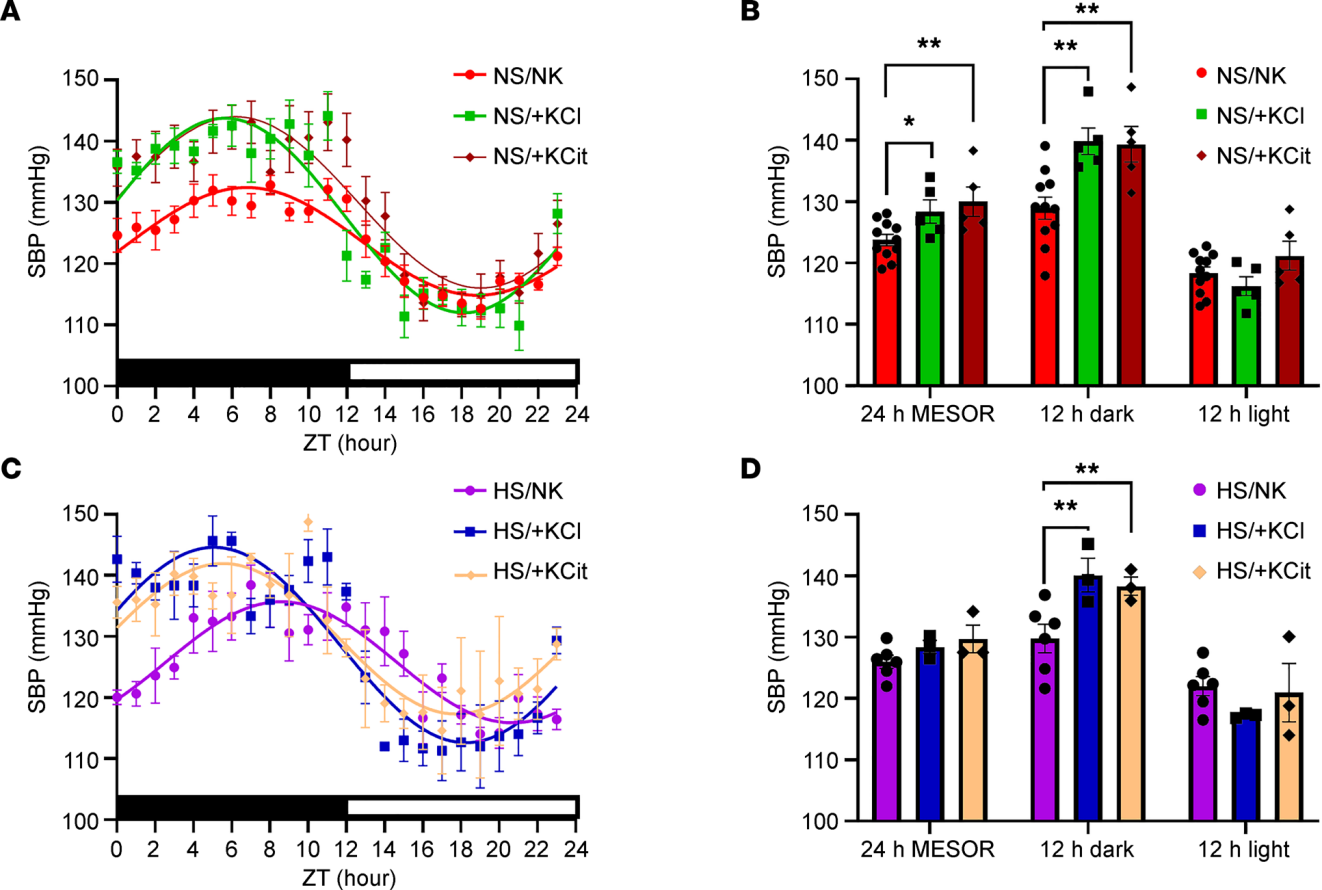
Chronic high dietary K^+^ feeding elevates BP. Systolic blood pressure (SBP) was recorded by in vivo telemetry from individual free roaming animals after 3 weeks of dietary intervention. (**A**) Comparison of fits analysis of curves showed a significant difference across a 24-hour period between animals fed a control diet (NS/NK) compared with animals fed a high-KCl (NS/+KCl, *P* < 0.0001) or high-KCit (NS/+KCit, *P* < 0.0001) diet. Dark/light times are shown by lower bar strip. ZT hour 0 = 18:00 hours. Data are shown as mean ± SEM, *n* = 5–11 per condition. (**B**) Quantification of the average SBP across a 24-hour period, or stratified by dark/light period. Data are shown as mean ± SEM with individual values shown. Data was analyzed using 2-way ANOVA with Tukey’s multiple-comparison testing. *P < 0.05; **P < 0.01. (**C**) Comparison of fits analysis of curves showed a significant difference across a 24-hour period between animals fed HS/NK diet compared with animals fed a HS/+KCl (*P* < 0.0001) or HS/+KCit (*P* < 0.0001) diets. Dark/light times are shown by lower bar strip. Time 0 = 18:00 hours. Data are shown as mean ± SEM, *n* = 3–6 per condition. (**D**) Average SBP across a 24-hour period, or stratified by dark/light period. Data are shown as mean ± SEM with individual values shown. Data were analyzed using 2-way ANOVA with Tukey’s multiple-comparison testing. **P < 0.01.

Three weeks of NS diet supplemented with +KCl (NS/+KCl) or high K^+^ citrate (NS/+KCit) significantly increased SBP relative to NS/NK across 24 hours (Figure 4A). This rise was most notable during darkness, but during the light period, no significant effect on SBP was detected (Figure 4B). Similarly, when mice were maintained on the high-Na^+^ diet supplemented with +KCl or +KCit for 3 weeks, SBP was significantly elevated over 24 hours (Figure 4C). Such an effect was again driven predominantly by elevation in SBP during darkness (SBP increased by 10.3 mmHg with HS/+KCl and by 8.5 mmHg with HS/+KCit) (Figure 4D). The low animal number in this analysis warrants further investigation of BP changes under different combinations of dietary NaCl and K^+^ intakes. Changes of SBP were mirrored by changes in DBP and MAP, but no effects on PP were observed (Supplemental Table 2). Effects of HK are independent of animal activity, with no difference in mouse activity in the dark period when SBP was increased (Supplemental Figure 3).

### Chronic high K^+^ feeding reduces NCC, despite elevated BP.

**Figure 5:**
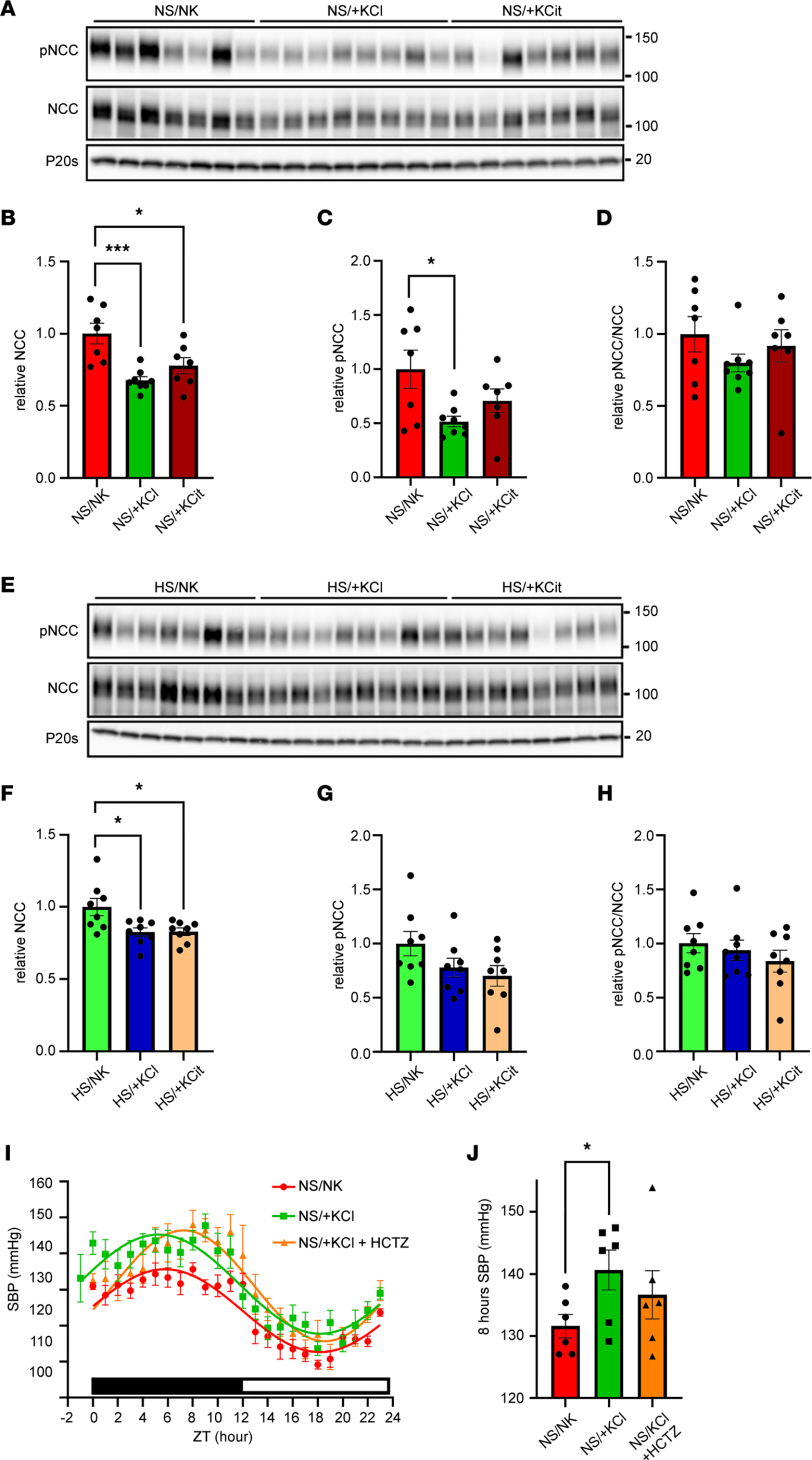
Chronic high K^+^ feeding reduces NCC. Western blot analysis of kidney lysates from animals fed various diets for 3 weeks. (**A**) Representative immunoblots of NCC, phosphorylated NCC (pNCC), and proteasome 20s (P20s, loading control) following normal salt diets. Molecular weight (KDa) of markers is shown on the right. (**B** and **C**) NCC and pNCC are significantly decreased by chronic K^+^ supplementation. (**D**) Ratio of pNCC/NCC is not significantly affected by K^+^ supplementation under NS diet. (**E**) Representative immunoblots of total NCC, phosphorylated NCC (pNCC), and proteasome 20s (P20s, loading control) following high-Na^+^ diets. (**F**) NCC is significantly decreased by chronic K^+^ supplementation. (**G** and **H**) pNCC and ratio of pNCC/NCC are not significantly changed by chronic K^+^ supplementation. (**I**) The higher SBP on a NS/+KCl diet is not significantly altered by hydrochlorothiazide (HCTZ) treatment over a 24-hour period. Dark/light times are shown by lower bar strip. ZT Time 0 = 18:00 hours. Data are shown as mean ± SEM, *n* = 6 per condition. (**J**) The higher SBP on a NS/+KCl diet is not significantly altered by HCTZ in the initial 8 hours following injection. For graphs, all data are shown as mean ± SEM with individual values shown. Data in **B**–**D** and **F**–**H** were analyzed by 1-way ANOVA followed by Dunnett’s multiple-comparison test. Data in **J** were assessed by 2-tailed *t* test, with the level of significance set as 0.033 to correct for the FDR (76). *P < 0.05; **P < 0.01; ***P < 0.001; ***P < 0.0001.

High dietary K^+^ feeding for up to 10 days can reduce NCC and pNCC (discussed in ref. 15). Here, NCC abundance was reduced in mice fed a NS diet supplemented with chronic +KCl (NS/+KCl, ~35 % reduction) or chronic +KCit (NS/+KCit, ~25 % reduction) for 3 weeks (Figure 5, A and B). pNCC levels were also significantly reduced with +KCl (~50 % reduction), with a similar trend observed with +KCit (~30 % reduction) (Figure 5, A, C, and D). In mice on the high-Na^+^ diet, chronic K^+^ supplementation significantly reduced total NCC, but this was attenuated relative to a NS diet (~17.5 % reduction) (Figure 5, E and F). K^+^ supplementation had no significant effect on pNCC levels (Figure 5, G and H) when compared with HS/NK-fed animals. Levels of pNCC and NCC were not significantly different in mice fed a HS/NK diet relative to a NS/NK diet (Supplemental Figure 4), and this may be related to the mice having similar plasma K^+^ levels on the 2 diets, despite lower aldosterone levels during HS/NK feeding. To confirm that the effects of chronic dietary K^+^ supplementation to increase BP were independent of NCC, the effects of HCTZ on BP were determined in mice fed the NS/+KCl diet (Figure 5I). In the 8-hour period after injection, SBP was not significantly lower in HCTZ-treated mice (136.6 ± 3.9 mmHg) relative to vehicle-treated mice (140.6 ± 3.2 mmHg) (Figure 5J).

### Effect of chronic high K^+^ feeding on urine and plasma chemistries.

K^+^ supplementation significantly increased water intake and 24-hour urine volume under both NS and HS conditions (Table 1). Except for a higher Cl^–^ excretion in +KCl mice (likely due to Cl^–^ supplementation), measured urinary parameters were similar in animals fed NS/+KCl and NS/+KCit diets. K^+^ excretion was higher for mice on K^+^ supplemented diets and Na^+^ excretion elevated for HS-fed animals. Polyuria was observed in mice fed HS/0K relative to HS/NK (Supplemental Figure 5). No significant differences in creatinine clearance (as a proxy for GFR) were observed with any the diets (Table 1). Plasma [K^+^] was significantly reduced in NS/0K mice compared with NK-fed animals (Table 2). A NS/+KCl diet caused a significant rise in plasma K^+^, but this was not observed with NS/+KCit, HS/+KCl, or HS/+KCit diets. Plasma copeptin levels were significantly lower in NS/0K- compared with NS/NK-fed animals. Under NS diet, chronic high K^+^ feeding had no significant effect on the plasma copeptin levels, but plasma copeptin after HS/+KCl feeding was significantly lower compared with HS/NK and NS/+KCl-fed animals (Table 2).

### A potential role of ENaC in mediating the increase in BP subsequent to chronic high dietary K^+^.

**Figure 6:**
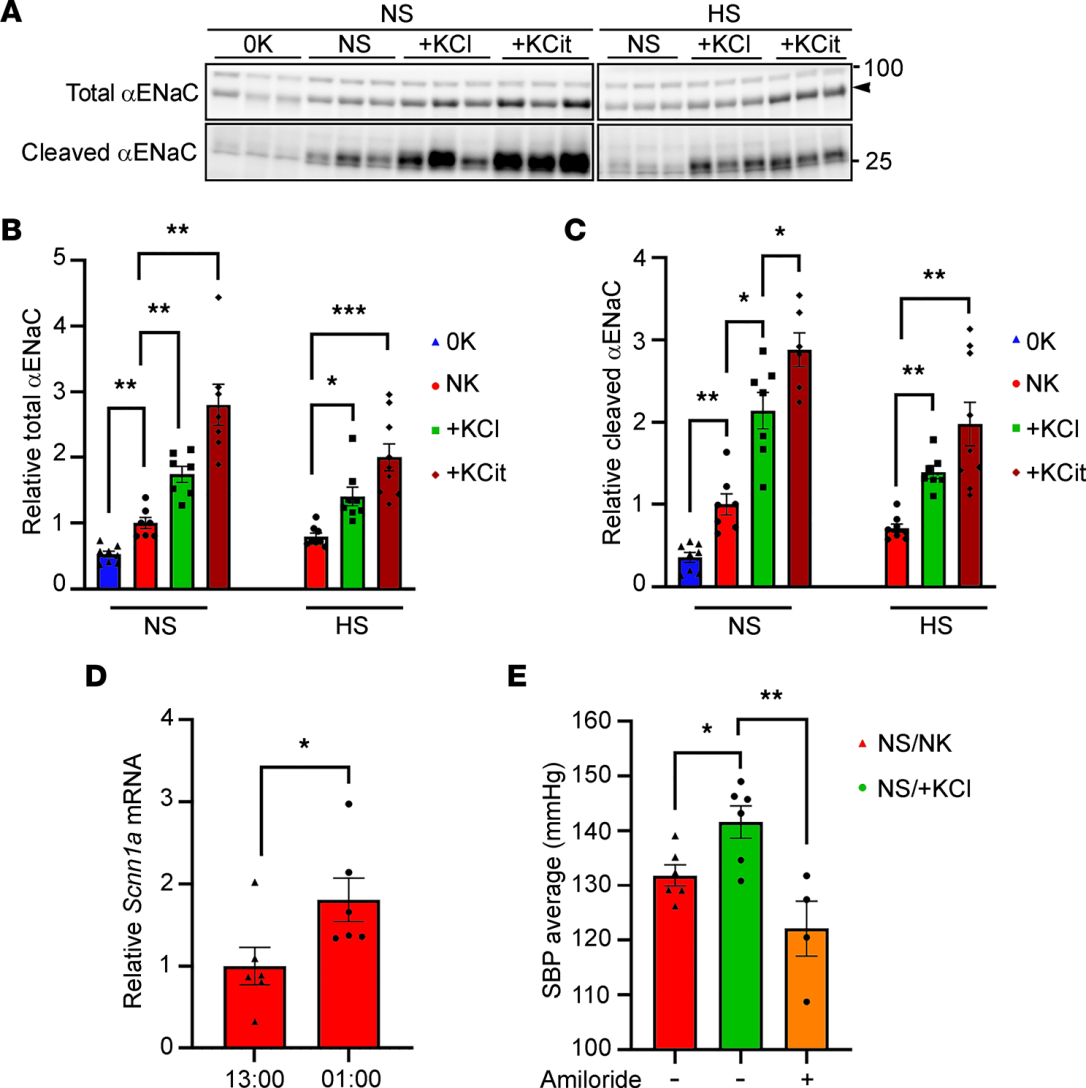
Increased ENaC expression plays a role in the increased BP following chronic high K^+^ feeding. Lysates of total kidney from animals fed the various diets were assessed by Western blotting. (**A**) Representative immunoblots of uncleaved and cleaved (active) αENaC. Molecular weight (KDa) is shown on the right, and the arrowhead represents the specific αENaC band. (**B** and **C**) Total αENaC (uncleaved + cleaved) cleaved αENaC were significantly increased by 3 weeks K^+^ supplementation on both a normal or high NaCl intake (NS, HS) and were significantly decreased following 2 weeks K^+^ depleted diet (0K). Data were analyzed using a 1-way ANOVA followed by Dunnett’s multiple-comparison test. *P < 0.05; **P < 0.01; ***P < 0.001. (**D**) mRNA levels for *Scnn1a* (encoding αENaC) is significantly higher in whole-kidney samples collected when animals are in dark phase (01:00 hours) compared with when tissue was collected in the light phase (13:00 hours). Data were analyzed by 2-tailed *t* test. (**E**) Amiloride significantly reduces SBP over the 8-hour period after injection for animals fed a NS diet supplemented with high KCl (NS/+KCl) for 3 weeks. Data are shown as mean ± SEM with individual values shown (*n* = 4–6). Data were analyzed using a 1-way ANOVA followed by Dunnett’s multiple-comparison test. *P < 0.05; **P < 0.01. All data are shown as mean ± SEM, with individual values shown.

ENaC is important for K^+^ and BP homeostasis (33). On a chronic NS/0K intake, total (uncleaved + cleaved) and cleaved (active) αENaC were reduced with respect to NS/NK-fed animals, but they increased with high K^+^ intake (Figure 6, A–C). Similar changes were observed with a high NaCl–supplemented diet (Figure 6, A–C). mRNA expression of *Scnn1a* (encoding αENAC) was significantly higher in kidneys collected from control diet–fed mice euthanized in the dark period compared with the light period (Figure 6D). To determine whether ENaC functionally contributes to the increased BP observed with chronic high K^+^ intake, we examined the effect of amiloride, a potent ENaC inhibitor (17), on BP in mice fed a NS/+KCl diet for 3 weeks. A single dose of amiloride significantly lowered SBP over the 8 hours following injection (Figure 6E), whereas vehicle had no effect (Supplemental Figure 2).

### Short-term and chronic +KCl feeding have opposing effects on BP.

**Figure 7:**
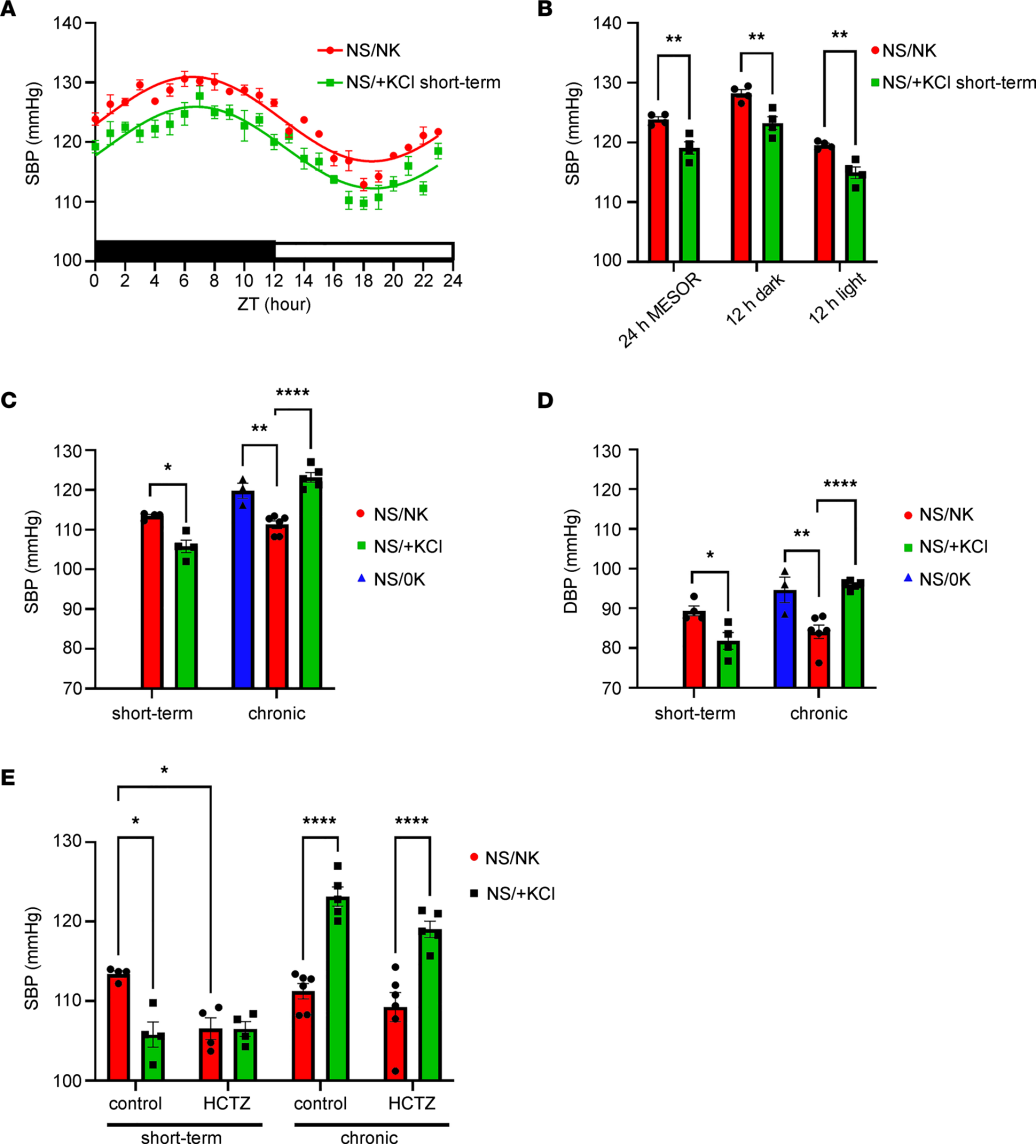
Short-term and chronic KCl feeding have opposing effects on BP. (**A** and **B**) Short-term (4 days) high KCl feeding combined with a normal NaCl diet (NS/+KCl) significantly reduces SBP recorded from implanted telemetry probes across 24 hours (*n* = 4/diet) (**A**) and over 24 hours as defined by MESOR or during the 12 hours dark or light phase (**B**), compared with control diet (NS/NK). The magnitude of error from the mean may be less than the smallest size of symbol available.(**C** and **D**) SBP and DBP recorded using tail cuff plethysmography during the dark phase are significantly lower during short-term (4 days) high KCl feeding but significantly higher during chronic KCl feeding (3 weeks) compared with control-fed animals (NS/NK). Chronic 0K feeding (2 weeks) significantly increases SBP and DBP compared with NS/NK-fed animals. (**E**) A single of dose of hydrochlorothiazide (HCTZ) 4 hours before tail cuff recording significantly reduced SBP of NS/NK-fed animals, but not short-term NS/+KCl-fed animals; it had no significant effect on elevated SBP in chronically fed NS/+KCl animals. Data were analyzed by 2-tailed *t* test between 2 groups (**B**), using a 1-way ANOVA followed by Dunnett’s multiple-comparison test (3 groups, **C** and **D**), or by 2-way ANOVA followed by Tukey’s multiple-comparison testing (**E**). *P < 0.05; **P < 0.01; ****P < 0.0001. All data are shown as mean ± SEM, with individual values shown.

The effects of K^+^ supplementation on BP in rodents are complex and differ dependent on the period of feeding or the genetic/experimental model examined (34, 35). For example, SBP was reduced within 1 week in rats provided 1% KCl in drinking water, but subsequently, SBP began to rise with continued KCl ingestion (36). Using radiotelemetry, we found that 4 days of NS/+KCl feeding significantly lowered SBP relative to NS/NK-fed mice over 24 hours (Figure 7A), independently of the dark or light phase (Figure 7B). However, SBP was elevated during the dark phase in NS/+KCl-fed animals after 7 days (Supplemental Figure 6), matching our observations after 3 weeks of NS/+KCl feeding (Figure 4). Intrigued by this time-dependent effect of NS/+KCl, the BP of an independent cohort of mice (cohort 2) was directly compared during short-term (4 days) and chronic (21 days) NS/+KCl feeding. Due to the large number of mice used in this cohort, we used tail-cuff plethysmography to measure BP. Matching our telemetry results, short-term NS/+KCl significantly decreased, whereas chronic NS/+KCl feeding significantly increased SBP and DBP (Figure 7, C and D). Chronic NS/0K feeding elevated SBP and DBP (Figure 7, C and D) to a level similar to that following chronic NS/+KCl feeding, with no evidence of hypokalemic nephropathy such as proximal tubular vacuolization or interstitial fibrosis (Supplemental Figure 7). HCTZ had no significant effect on SBP in short-term NS/+KCl–fed mice and did not attenuate elevated BP in chronically NS/+KCl–fed animals (Figure 7E).

### Short-term and chronic dietary K^+^ effects on plasma K^+^ and aldosterone.

**Figure 8:**
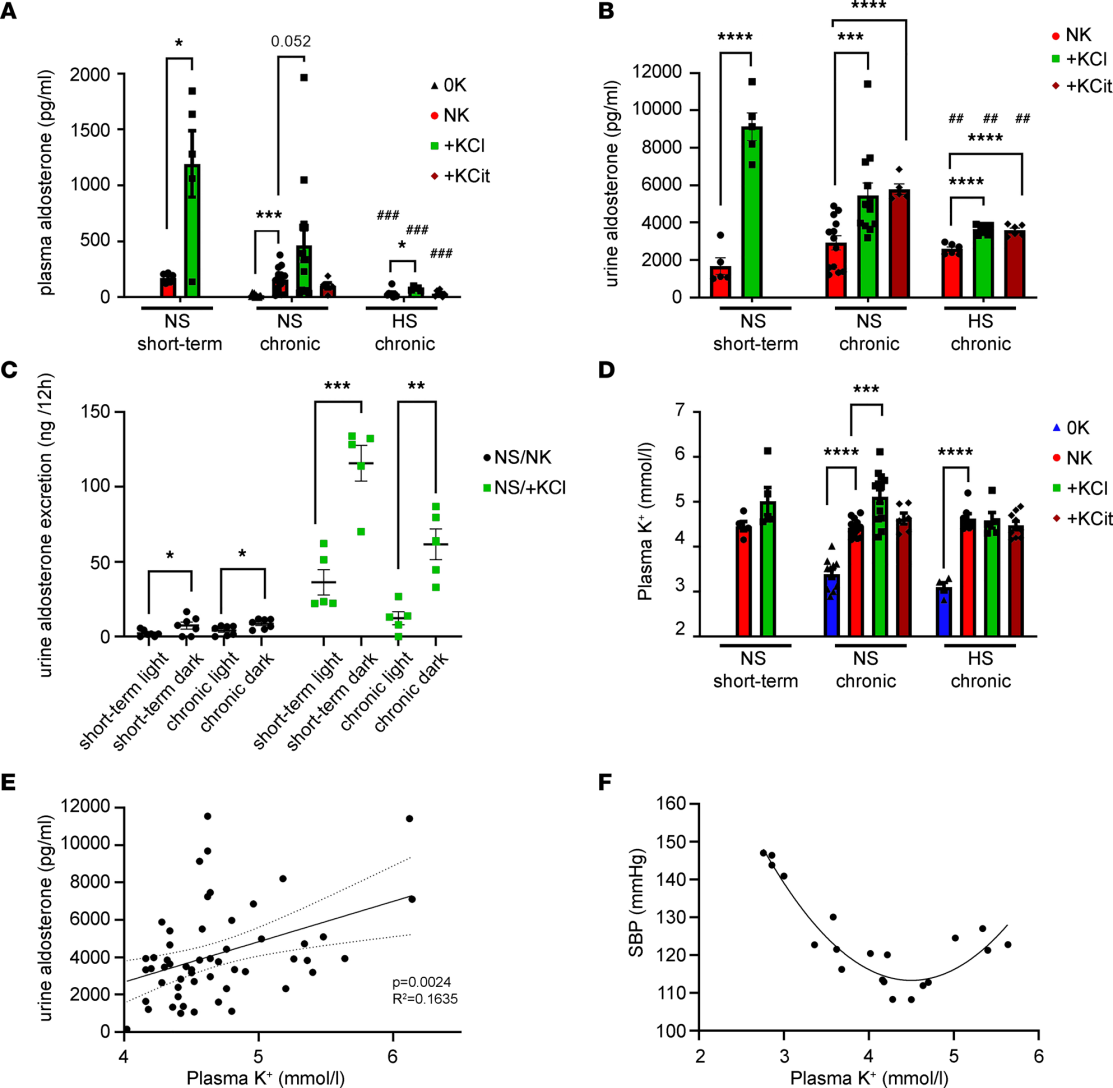
Potassium and aldosterone are elevated in high-KCl–fed animals. (**A**) Plasma [aldosterone] is increased following short-term and chronic KCl feeding. Chronic 0K feeding (2 weeks) significantly reduced [aldosterone]. HS diet reduced [aldosterone] across all groups. Only 2 HS/0K-fed animals had a detectable level of aldosterone, so this condition was not analyzed. Data were analyzed as 2-way ANOVA with Dunnett’s multiple-comparison testing. (**B**) Urine aldosterone concentrations under the different chronic dietary K^+^ conditions. Data were analyzed by a 2-way ANOVA main effects model, with multiple comparisons with NS/NK group. (**C**) Urine aldosterone excretion was significantly higher during the 12-hour dark phase (18:00–06:00) compared with the light phase, with the magnitude of increase clearly being greater for high-KCl–fed (+KCl-fed) animals. No clear difference was detected between short-term (4 days) and chronically (3 week) fed animals. Analysis within groups by 2-tailed *t* test. (**D**) Plasma [K^+^] under the different short-term or chronic dietary K^+^ conditions. Data are shown as mean ± SEM, with individual values shown. Data were analyzed as 2-way ANOVA with Dunnett’s multiple-comparison testing. (**E**) Urine aldosterone concentration significantly positively correlates with plasma K^+^ concentration. Best fit analysis, linear regression, with 95% CI limits (dotted lines) displayed. Data are shown as mean ± SEM. (**F**) The relationship between SBP against plasma [K^+^] fits a second order quadratic equation. Data are from individuals where BP and blood sampling are made from same animal. All individual values considered for best-fit nonlinear regression.*P < 0.05; **P < 0.01; ***P < 0.001. ^##^*P* < 0.01, ^###^*P* < 0.001 versus respective chronic NS condition.

Urine and plasma chemistries from cohort 2 are shown in Table 3 and Table 4, respectively. For urine, the effects of short-term feeding broadly mirror effects seen following chronic NS/+KCl feeding. Short-term NS/+KCl feeding had no significant effect on plasma renin activity, but after chronic NS/0K and NS/+KCl diets, renin activity was significantly reduced (Table 4). Considering all data collected from cohort 1 and cohort 2, relative to NS/NK-fed animals, plasma aldosterone was increased by short-term and chronic NS/+KCl feeding and reduced by chronic NS/0K feeding (Figure 8A). Chronic HS feeding reduced plasma aldosterone, but HS/+KCl feeding still significantly elevated plasma aldosterone (Figure 8A). Differences in urine aldosterone mimicked changes observed in plasma (Figure 8B). Total urinary aldosterone excretion was higher during the 12-hour dark phase compared with urine collected from the same individuals following 12 hours of light (Figure 8C). Plasma K^+^ was significantly lower in mice fed chronically a NS/0K or HS/0K diet relative to NS/NK-fed animals (Figure 8D). Plasma K^+^ was higher in chronically NS/+KCl–fed animals, but although trending, it was not significantly higher after 4 days of NS/+KCl diet (Figure 8E and Table 4). There was a positive linear relationship between urine aldosterone and plasma K^+^ (when [K^+^] > 4 mM considered; Figure 8E). Interestingly, a second-order quadratic (U-shaped) relationship was observed between SBP and plasma [K^+^] (when considering data from NS-fed mice from cohort 1 and 2) (Figure 8F).

### Short-term and chronic KCl feeding reduces NCC and increases cleaved ENaC.

**Figure 9:**
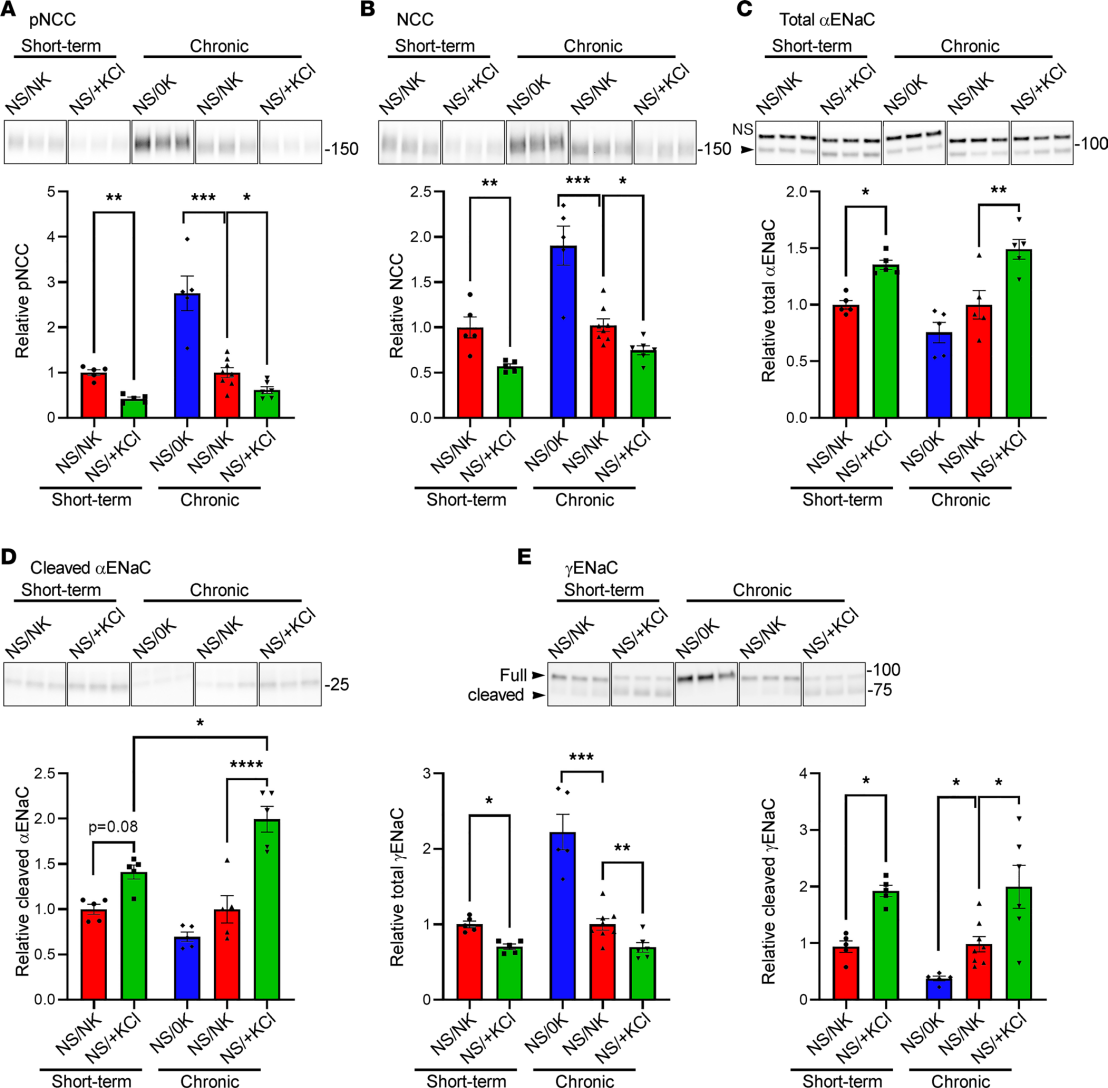
High KCl feeding reduces NCC and increases ENaC. Lysates of total kidney from animals fed the various diets were assessed by Western blotting. Representative panels (samples run on the same gel but noncontiguous) from representative blots are shown above quantification graphs. (**A** and **B**) pNCC (**A**) and NCC (**B**) were significantly decreased after short-term and chronic KCl feeding but were increased after chronic K^+^ depletion (0K, 2 weeks). (**A** and **B**) Total αENaC (**C**) and cleaved αENaC (**D**) were increased following +KCl feeding, but the magnitude of increase in cleaved αENaC after chronic feeding is significantly greater relative to short-term feeding. (**E**) Total γENaC (full length plus cleaved protein bands) was significantly decreased after short-term and chronic KCl feeding, whereas cleaved γENaC was significantly increased. Data from all individuals plotted with mean ± SEM. Analysis by 1-way ANOVA followed by Tukeys multiple comparisons. *P < 0.05; **P < 0.01; ***P < 0.001; ****P < 0.0001.

Compared with NS/NK-fed animals, NCC and pNCC levels were reduced in mice after short-term (4 days) or chronic (3 weeks) NS/+KCl feeding, whereas pNCC and NCC were significantly elevated by chronic NS/0K feeding (Figure 9, A and B). Total and cleaved αENaC were increased by short-term and chronic NS/+KCl feeding, but cleaved αENaC was higher after chronic feeding (Figure 9, C and D). Total γENaC was reduced following short-term or chronic NS/+KCl feeding, but it increased after a NS/0K diet (Figure 9E). Conversely, cleaved γENaC was greatly increased by short-term and chronic NS/+KCl diets and decreased after a chronic NS/0K diet. IHC of kidneys from the same mice cohort demonstrated reduced staining intensity for NCC and pNCC after short-term and chronic NS/+KCl feeding as well as increased labeling intensity after NS/0K feeding compared with NS/NK-fed control animals (Supplemental Figure 8). After feeding a NS/0K or NS/NK diet, γENaC labeling was predominantly intracellular, whereas after short-term NS/+KCl feeding, it is both located intracellularly and in the apical plasma membrane domain. On a chronic NS/+KCl diet, γENaC is predominantly observed in the apical plasma membrane domain.

Immunoblots for modulators of NCC and/or ENaC activity, or proteins important for Na^+^, K^+^, or water balance, showed minimal changes after the dietary manipulations (Supplemental Figure 9). The NCC modulatory kinase WNK4, and its active form pWNK4, were increased during chronic dietary K^+^ depletion, whereas the Na^+^-K^+^-2Cl^–^ cotransporter NKCC2 and Aquaporin-2 were decreased. Additionally, the abundances of the K^+^ channel ROMK and the Cl^–^/HCO_3_^–^ exchanger pendrin were increased following chronic NS/+KCl feeding.

### Correlations of NCC and ENaC to aldosterone and plasma K^+^.

**Figure 10:**
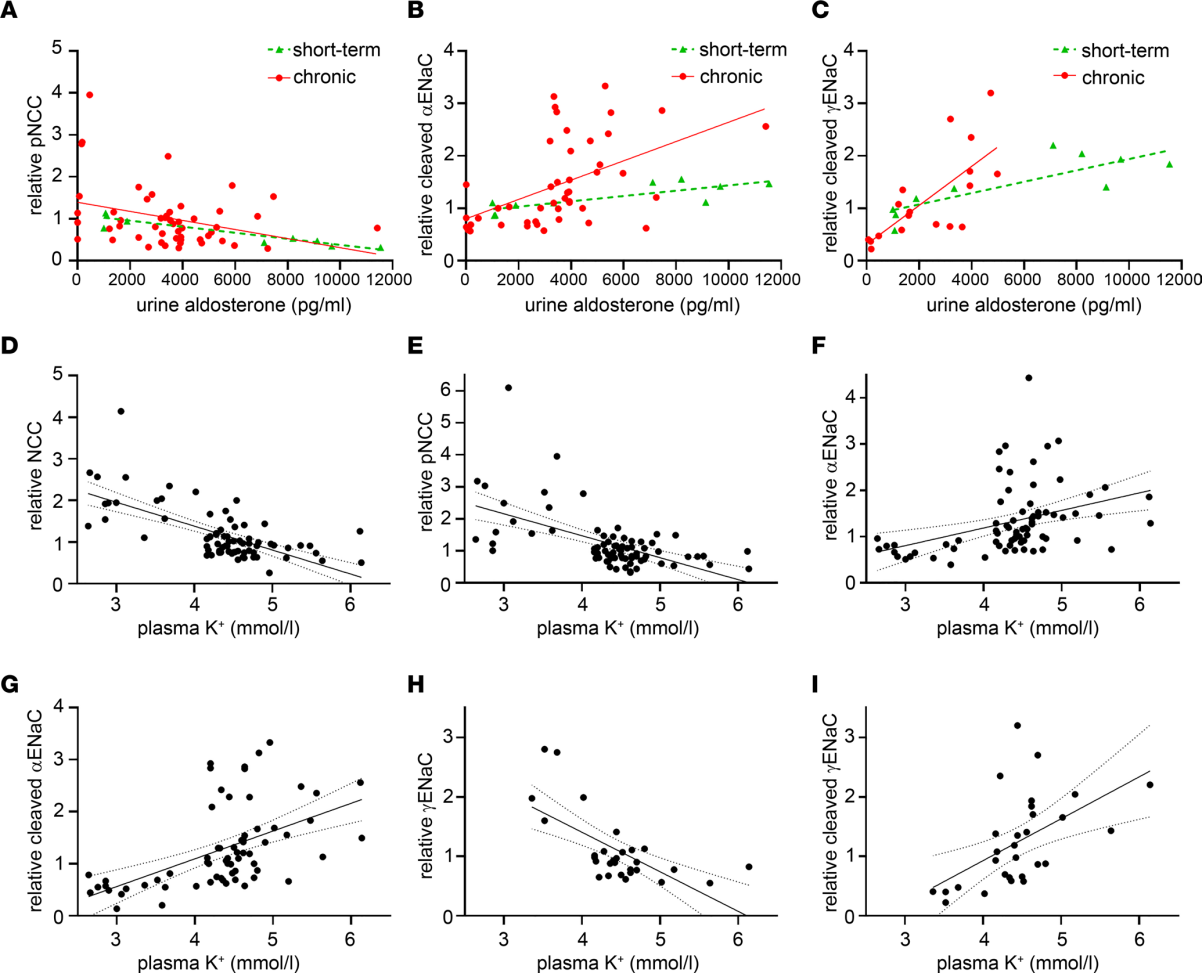
Correlation of NCC and ENaC to urine aldosterone and plasma potassium level. (**A**–**C**) Best fit analysis for short-term and chronically fed animals. (**A**) pNCC significantly negatively correlates with urine [aldosterone]. The correlation for short-term feeding (5 days, *R*^2^ = 0.839) and chronically fed animals (3 weeks, *R*^2^ = 0.110) did not significantly differ for slope or *y* intercept. (**B**) Cleaved αENaC significantly positively correlates with urine [aldo]. Correlation for short-term feeding (*R*^2^ = 0.652) and chronic feeding (*R*^2^ = 0.258) tended to differ for slope (*P* = 0.0546) with a significantly different *y* intercept (*P* = 0.0469). (**C**) Cleaved γENaC significantly positively correlates with urine [aldo]. The correlation for short-term feeding (*R*^2^ = 0.669) and chronic feeding (*R*^2^ = 0.516) significantly differed (*P* < 0.01). (**D**–**I**) Best fit analysis with 95% CI (dotted lines) for whole data set. (**D** and **E**) NCC and pNCC significantly negatively correlate with plasma [K^+^]. (**F** and **G**)Total αENaC and cleaved αENaC significantly positively correlate with plasma [K^+^]. (**H** and **I**) Total γENaC significantly negatively correlates with plasma [K^+^], while cleaved γENaC significantly positively correlates. Each dot represents protein abundance (arbitrary units), and urine analysis from the same animal. Data are from all animals in cohort 1 and 2 combined, with the exception of γENaC data, which are only from cohort 2.

pNCC negatively correlated with urine [aldosterone] under short-term (*R*^2^ = 0.839) or chronic conditions (*R*^2^ = 0.110) (Figure 10A). NCC showed a similar relationship (Supplemental Figure 10A). Cleaved αENaC or γENaC positively correlated with urine [aldosterone], with the strength of the relationship greater after chronic compared with short-term feeding (Figure 10, B and C). Total αENaC showed a similar relationship, whereas total γENaC had a negative linear relationship with urine [aldosterone] (Supplemental Figure 10, B and C). NCC and pNCC significantly negatively correlated with the plasma [K^+^], while total and cleaved αENaC positively correlated (Figure 10, D–G). Total γENaC had a significant negative correlation with plasma K^+^ concentration, whereas cleaved γENaC had a positive correlation (Figure 10, H and I).

### High dietary KCl promotes DCT remodeling.

**Figure 11:**
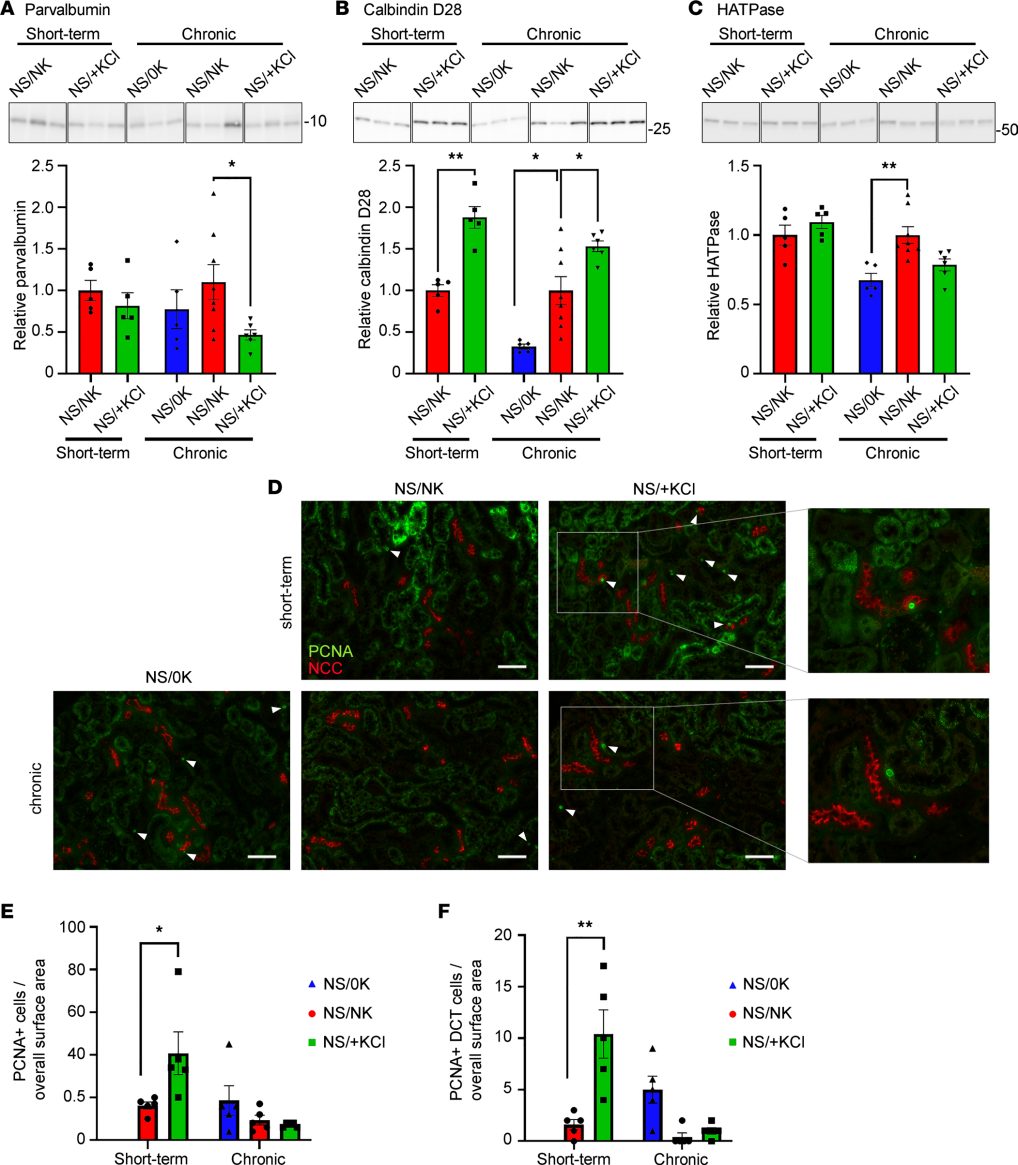
High KCl feeding promotes kidney remodeling. (**A** and **B**) After chronic NS/+KCl feeding, parvalbumin (**A**) was significantly reduced, whereas calbindin D28 (**B**) was significantly increased after both short-term and chronic NS/+KCl feeding. (**C**) The H^+^-ATPase B1 subunit (HATPase) was significantly reduced following chronic K^+^ depletion (0K, 2 weeks). Representative panels (samples run on the same gel but noncontiguous) from representative blots are shown above quantification graphs. (**D**) Representative images of kidney sections labelled with proliferating cell nuclear antigen (PCNA, green) alongside NCC (red). White arrowheads highlight PCNA positive nuclei. Boxed area highlights a DCT that is expanded in the right panel. Scale bar: 50 μm. (**E**) Quantification of PCNA staining from imaged whole kidney and from DCT cells. (**F**) A significantly increased number of PCNA^+^ cells are observed in whole kidney and the DCT after 4 days of NS/+KCl feeding. Data were analyzed using a 1-way ANOVA followed by Dunnett’s multiple-comparison test. *P < 0.05; **P < 0.01. Data are shown as mean ± SEM.

Low dietary K^+^ intake or constitutively activating the kinase SPAK in the DCT (both increasing NCC phosphorylation) are associated with DCT hypertrophy and connecting tubule (CNT) atrophy (37, 38), whereas inhibiting NCC is associated with DCT atrophy and CNT hypertrophy (39, 40). Here, chronic NS/+KCl feeding reduced abundances of the DCT-specific protein parvalbumin, and both short-term and chronic NS/+KCl feeding increased abundance of the late DCT and CNT protein calbindin D28 (Figure 11, A and B). In contrast, chronic NS/0K feeding reduced calbindin D28 and the H^+^-ATPase B1 subunit (expressed from the late DCT through the CNT and CD) (Figure 11C). Short-term KCl feeding increased the number of proliferating cell nuclear antigen–positive nuclei throughout the kidney and in the DCT (late and early DCT not separated). These effects were not observed during chronic dietary manipulation (Figure 11, D–F). Together, these results suggest that high dietary KCl intake results in DCT atrophy and CNT hypertrophy.

### Proteomic analysis of short-term and chronic effects of high dietary KCl intake.

**Figure 12:**
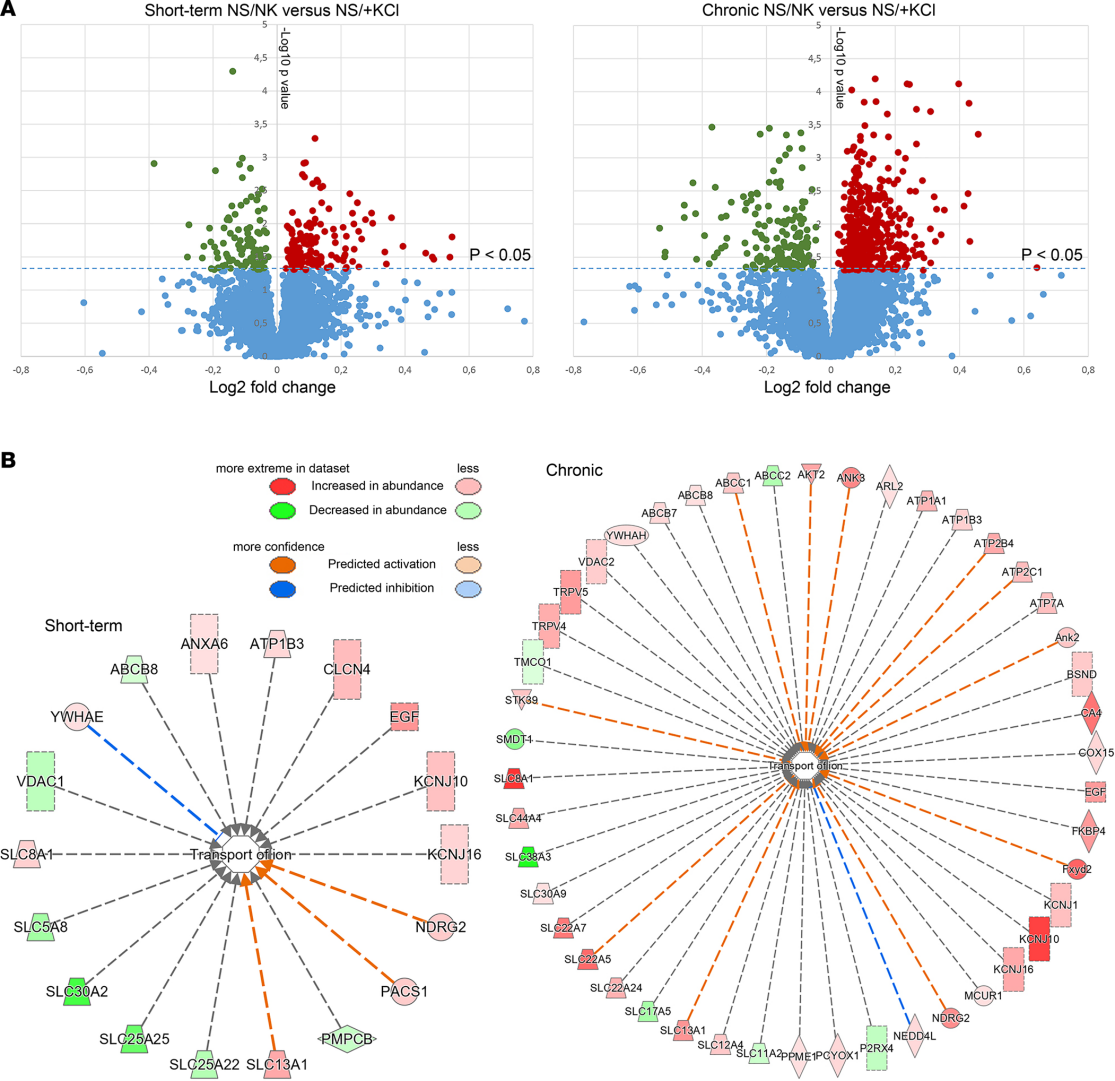
Large-scale proteomic profiling of short-term and long-term effects of high KCl intake in kidney cortex. Kidney cortex homogenates were examined using LC-MS/MS–based quantitative proteomics. (**A**) Volcano plot of the protein quantification where the primary axis shows the log_2_ (mean peptide abundance ratio), while the secondary axis designates the −log_10_(*P* value). After short-term feeding of NS/+KCl, 105 proteins were significantly decreased relative to NS/NK diet and 147 were significantly increased. After chronic feeding, 163 proteins were significantly reduced and 479 significantly increased in abundance. (**B**) IPA of the significantly changed proteins (equivalent human gene name shown) highlighted that many more proteins associated to the transport of ions were changed in abundance during chronic NS/+KCl feeding.

To investigate the influence of dietary K^+^ intake on the protein landscape of the kidney and to identify novel mechanisms for the differential effect of high dietary K^+^ intake on BP, we used liquid chromatography–tandem mass spectrometry–based (LC-MS/MS–based) quantitative proteomics. After short-term feeding of NS/+KCl, 5,484 proteins were identified in kidney cortex homogenates, of which 105 were significantly decreased relative to NS/NK diet and 147 were increased (Figure 12A). In total, 6,078 proteins were identified after chronic NS/+KCl feeding, of which 163 were reduced and 479 significantly increased relative to NS/NK-fed mice. All proteins identified and their relative abundances are detailed in Supplemental Data File 2. Of the significantly changed proteins, Ingenuity Pathway Analysis (IPA) highlighted that several had a biological function associated to transport of ions, with many more proteins associated to this category increased during chronic NS/+KCl feeding (Figure 12B). Gene ontology (GO) analysis of significantly increased proteins after short-term KCl feeding revealed an overrepresentation of proteins involved in protein dephosphorylation and various catabolic/metabolic processes, whereas downregulated proteins were associated to cellular respiration and lipid metabolism (Supplemental Figure 11). After chronic KCl feeding, the proteins increased in abundance were associated with transport, mitochondrial reorganization, and protein hydroxylation, whereas significantly reduced proteins were associated to metabolic and small molecule catabolic processes.

**Figure 13:**
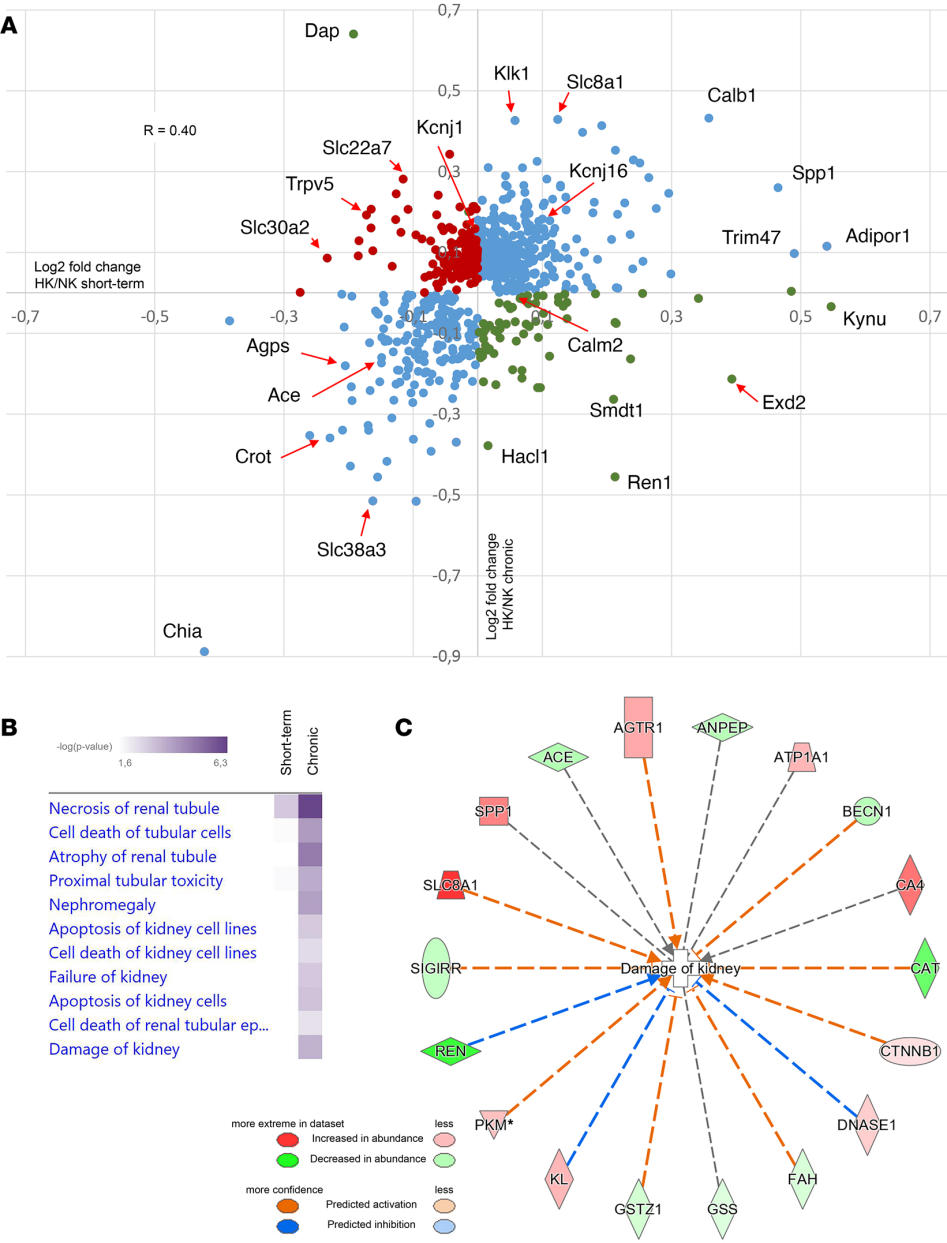
Proteomic profiling identifies proteins differentially regulated following short-term or chronic KCl feeding. (**A**) Each dot represents a protein significantly altered in abundance in at least 1 of the dietary conditions. Pearson’s correlation coefficient for all proteins, *R* = 0.4. Proteins with correlating changes in abundance are shown by blue dots, proteins increased in abundance during short-term but decreased during chronic feeding are represented by red dots, and proteins decreased in abundance during short-term but increased during chronic feeding are represented by green dots. Proteins of interest (corresponding mouse gene name shown) are highlighted. (**B**) Proteins that were significantly altered in abundance after short-term and chronic NS/+KCl feeding were mapped to various nephrotoxicity pathways using IPA. (**C**) After chronic NS/+KCl feeding, a greater number of proteins (corresponding human gene name shown) associated to kidney damage were observed.

To investigate potential mechanisms contributing to the differential effect of high dietary K^+^ intake on BP, we compared proteins changed after short-term or chronic KCl feeding. Although numerous proteins had similar changes in abundance, several did not change to a similar magnitude or they were differentially regulated (Figure 13A). For example, the key COP9 signalosome (CSN) subunit JAB1, the peptidase Kallikrien1, the K^+^ channel ROMK, and the FGF23 coreceptor Klotho were only significantly increased following chronic NS/+KCl feeding (Supplemental Figure 12). Using IPA, we mapped proteins that were significantly altered in abundance after short-term and chronic NS/+KCl feeding to various toxicity pathways that relate to nephrotoxicity and mark clinical pathology endpoints (Figure 13B). Interestingly, after chronic NS/+KCl feeding, a greater number of proteins associated with kidney damage were observed (Figure 13C).

## Discussion

High dietary Na^+^ consumption increases BP (2–4), whereas an increased dietary K^+^ intake can reduce BP (5, 6, 10, 11), especially if initial K^+^ consumption is low (41). As dietary K^+^ depletion increases BP (7), consumption of food containing a relatively high amount of K^+^ and low Na^+^ is proposed as a simple way to improve and maintain good cardiovascular health (42, 43). However, dietary studies in animals and humans and observational or K^+^ intervention studies in humans indicate that the effects of high K^+^ intake on BP are not always beneficial and may be influenced by the period of intervention, the anion accompanying the extra K^+^, and whether BP is high before the intervention (13, 14, 27–29). Reducing and maintaining a lower BP based on dietary intervention requires understanding of the long-term and/or accumulated effects of the intervention and the underlying molecular basis. Since the experimental period for most interventional studies in humans has been greater than 2 weeks, our initial approach here was to monitor BP after 3 weeks of dietary supplementation with KCl or KCit in C57BL/6J mice that were normotensive or hypertensive. High BP (SBP raised by about 6 mmHg) was induced by feeding of a 4% NaCl diet for 7 weeks, in line with previous studies in the C57BL/6J strain (44–46). Furthermore, if we consider the mouse lifespan to be ~36 months, the 3-week K^+^ feeding period would be equivalent to 80 weeks of dietary supplementation in humans (~80-year lifespan). Our major finding was that chronic high K^+^ feeding significantly elevated BP independently of the conjugated anion, whereas short-term K^+^ feeding reduced BP.

BP was significantly higher in mice receiving a 0K diet for 2 weeks relative to mice receiving a NK diet. BP was further increased during concurrent high NaCl intake. This increase in BP was associated with increased total and pNCC but with reduced ENaC abundance. HCTZ reduced the BP of HS/0K-fed mice back to the levels observed in HS/NK-fed animals, strongly suggesting, like others (23, 28), that low dietary K^+^–mediated effects on BP are driven predominantly by NCC (15).

Chronic high K^+^ feeding to mice receiving a normal or a high Na^+^ diet increased BP. These effects were independent of the accompanying anion or Na^+^ intake and predominantly attributed to a higher BP during darkness. Although BP is naturally higher during darkness for nocturnal mice, no differences in mouse activity following chronic K^+^ feeding suggest that altered activity is not a driving factor of the higher BP. Our data generally replicate the BP phenotype reported after 10 days of high K^+^ feeding in mice (29), where higher BP during darkness can be inferred from their presented data. HCTZ did not significantly reduce BP during high K^+^ feeding, suggesting that the relationship between NCC and BP is “disconnected” during chronic K^+^ feeding. Supporting this, KCl and KCit–fed animals had lower levels of NCC, and in general, pNCC levels were also lower, similar to previous reports (28, 29). Previously, 4 days of +KCl feeding was shown to reduce total and pNCC, but high KCit feeding did not (27). We propose that the anion effect might be time dependent, KCl may lower NCC more rapidly than KCit, or there may be different acid-base effects of chloride versus citrate. Ultimately, chronically, the anion accompanying the K^+^ appears not to be crucial for reducing NCC abundance or the ability of high dietary K^+^ to increase BP.

Similar to other studies of shorter duration (20, 47), total and cleaved αENaC and cleaved γENaC were significantly increased following 3 weeks of chronic K^+^ feeding, probably driven by a high K^+^–mediated release of aldosterone. The increased BP subsequent to chronic high dietary K^+^ intake was also sensitive to amiloride, suggesting a role for ENaC in the BP response (48, 49). However, amiloride did not significantly reduce BP in a previous study with mice fed a 3% NaCl/5% K^+^ diet for 10 days, despite raised βENaC levels (28). One possibility for this is that the higher Na^+^ intake (10-fold higher Na^+^ than used in our NS/+KCl diets) may be influencing BP differently than the current study. Secondly, amiloride in the previous study was given in the drinking water, and in our experience, this is not optimal for amiloride dosing as mice avoid drinking. Thus, the ~10-fold greater dietary Na^+^ level (28) may be enough to drive Na^+^ retention and maintain high BP due to residual ENaC activity during amiloride treatment. It may also relate to chronic K^+^ feeding resulting in greater ENaC activity. Indeed, we observed that cleaved αENaC and cleaved γENaC were increased following short-term and chronic K^+^ feeding (suggesting higher ENaC activity), but cleaved αENaC levels were increased significantly more after chronic feeding. Furthermore, qualitative IHC suggests that there is more γENaC in the apical plasma membrane domain during chronic K^+^ feeding relative to short-term, fitting with observations that longer periods of K^+^ feeding enhance ENaC membrane accumulation (29). Together, these data suggest that ENaC contributes to the higher BP during chronic K^+^ feeding.

BP was elevated subsequent to chronic high K^+^ feeding predominantly in the dark phase, a period where we observed significantly higher *Scnn1a* mRNA (encoding αENaC). A circadian pattern of ENaC expression has been previously observed (50, 51). It is plausible that, during the dark phase, mice are more sensitive to augmentation of ENaC, with increased channel activity exceeding a threshold that promotes increased BP. Alternatively, during chronic K^+^ loading in humans, an acute K^+^ load attenuates K^+^-induced natriuresis despite a similar response in plasma aldosterone (52). This suggests that, in a chronic setting, compensatory mechanisms are activated to reduce K^+^-induced natriuresis (15). Further studies are warranted to assess whether circadian patterns on ENaC are direct or caused by feeding behavior. Furthermore, one could speculate that the use of mineralocorticoid receptor antagonists (53) would prevent the increase in ENaC and BP upon a chronic high K^+^ diet, although this may result in severe hyperkalemia.

In line with studies using shorter periods of dietary K^+^ manipulation (20, 29), after 3 weeks of +KCl feeding, plasma K^+^ levels were elevated and NCC levels were reduced relative to animals with a NK intake. However, NCC levels were also reduced after 3 weeks of KCit feeding, which did not significantly raise plasma K^+^. This suggests that, although it may be the initial trigger, a sustained increase in plasma K^+^ per se is not an absolute requirement for NCC levels to be reduced, and other factors, such as altered sensitivity of the DCT to K^+^ or the potential DCT atrophy observed here, may also play a role.

Our proteomic screen highlighted the pleiotropic effect of K^+^ supplementation on the kidney. Many catabolic and metabolic processes were altered during high K^+^ feeding, alongside numerous ion transporters and their regulatory proteins that may play a role for the BP and/or autoregulatory response. Several of these proteins, including JAB1, tissue kallikrien, and Klotho, have been implicated in regulation of NCC and/or ENaC previously (54–58) and were only increased following chronic K^+^ supplementation, suggesting they could be important for the differential effects of K^+^ intake on BP. Furthermore, although GFR was not significantly reduced during chronic dietary K^+^ supplementation, several of the changed proteins in the kidney cortex are associated with nephrotoxic pathways and kidney damage. Currently, we do not know whether these proteins are driving kidney injury or protection from injury, or whether the changes contribute toward or are attributed to a prior increase in BP. However, considering the links between NCC and/or ENaC, inflammation, and Na^+^-sensitive hypertension (59–61), further studies of these responses are warranted.

High BP can also be driven by enhanced vascular tone, even when renal function is normal (62). For example, while angiotensin II infusion raised BP in mice, the BP returned to normal 4 weeks after cessation of infusion. However, aortae exhibited significantly diminished contractility and endothelium-dependent vasodilation, pointing to sustained vascular damage (63). Aldosterone can also have effects on the vasculature, and aldosterone-induced inflammation, vasculopathy, and vascular calcification are linked to the development of arterial stiffness (64, 65). Here, 3 weeks of high dietary KCl elevated plasma aldosterone and increased BP. However, short-term +KCl feeding significantly reduced BP but raised plasma aldosterone to an even greater extent than chronic feeding. Furthermore, we did not detect any effect of chronic high K^+^ feeding on PP, a correlate for vascular stiffness, suggesting that aldosterone-induced arterial stiffness is unlikely to have occurred. Supporting this idea, in other studies where SBP has been reduced via K^+^ supplementation, microvascular and endothelial function are not altered (66, 67). Based on our current data, these findings point to a limited direct role of the vasculature in modulating BP changes in response to various dietary K^+^ loads.

This study is not without limitations. In long-term human interventional studies (12, 43, 68), urine K^+^ excretion is ~1.4-fold higher following dietary K^+^ supplementation compared with control diet, whereas here, the comparable K^+^ excretion is 2.7-fold higher (Supplemental Table 4). Thus, the 5% K^+^ diet used here is higher than that tested in humans. Furthermore, the “U-shaped” association we observe between SBP and plasma K^+^, to some extent similar to that observed in a meta-analysis between K^+^ intake and BP (14), centers the curve on a plasma [K^+^] of ~4.5 mM. This suggests that our control diet contains almost the optimal dietary K^+^ amount to achieve the lowest BP in mice. Further studies to moderately increase dietary K^+^ intake in mice and examine whether it can reduce and sustain lower BP and to examine how this relates to the optimal range for humans are required. The K^+^ diets we used caused polyuria, which may result from osmotic diuresis or vasopressin resistance, as observed during low dietary K^+^ intake (69, 70). If not fully compensated for by increased water intake, the polyuric state may increase vasopressin levels and contribute to the higher ENaC activity and increased BP during high K^+^ intake (71). However, similar plasma copeptin levels (surrogate marker for vasopressin) in mice on the control or high K^+^ diets suggest this mechanism is not a major contributor in the current study.

In summary, our study suggests that, if dietary intake of K^+^ is low, higher NCC levels increase BP in a Na^+^-sensitive manner. We also show that 4 days of +KCl feeding reduces NCC, and this correlates with reduced BP. In contrast, after 3 weeks of high dietary K^+^ intake, BP is increased by a mechanism that involves ENaC and is relatively insensitive to changes in Na^+^ intake. As proposed earlier (72), this indicates that there is a critical interplay between ENaC and NCC for modulation of BP. However, chronic high dietary K^+^ intake, at least at the levels used here, may promote kidney damage and contribute to the hypertensive outcome, but the hypertension per se may also initiate kidney damage. Therefore, the concept that dietary K^+^ intake is inversely related to BP, or potentially can promote a decrease in BP, may only be valid under specific intakes. Further mechanistic studies are warranted to inform on the potential clinical efficacy of dietary K^+^ for maintaining a healthy BP in humans.

## Methods

### Animals and study design.

In an initial cohort (cohort 1), male C57BL/6J mice were implanted with PA-C10 and HDX-11 radiotelemetry devices (Data Sciences International [DSI]) a week before any dietary manipulations commenced, during which they were individually caged. A 4-factor cosine curve was fitted to the repeating pattern of BP (73, 74) as detailed in Supplemental Methods. To limit potential probe malfunctions during mouse handling, this group followed the dietary interventions, but their blood/urine was not sampled. Diets were prepared from powdered rodent diet (Teklad Diet, TD.08251, Envigo) — being nominally K^+^, Na^+^, and Cl^–^ free — with ionic compounds (Sigma-Aldrich) added back to generate modified diets. Twelve-week-old mice were fed a control diet of KCl (1.05% K^+^) and NaCl (0.3% Na^+^) (NS/NK) or a high NaCl (1.57% Na^+^) (HS/NK) diet for 7 weeks. Subsequently, mice were stratified to receive either a +KCl (5.25% K^+^) or a high KCit (KCit, 5.25% K^+^) diet in combination with the control or high NaCl intake for 3 weeks. Following high K^+^ feeding, animals were fed a 0K diet with either control or high NaCl level for 2 weeks. Animals maintained on HS/0K diet were treated with 37.5 mg/kg bodyweight HCTZ (Sigma-Aldrich) administered as 2 i.p. doses 24 hours apart, with BP recorded following the second dose. In a subsequent experiment, animals fed a chronic NS/HK (+KCl) diet were treated with HCTZ (as above) and, after a 3-day washout period, received a single i.p. dose of amiloride (5 mg/kg body weight, Sigma-Aldrich), with BP being recorded for the first 12 hours following injection. HCTZ and amiloride were prepared from solid by dilution in DMSO and then further diluted in sterile physiological saline before injection.

### Tail cuff plethysmography.

In a second cohort of mice (cohort 2), conscious BP was recorded from the tail using an occlusion cuff and volume-pressure recording (VPR) sensor (Coda equipment, Kent Scientific), as detailed in Supplemental Methods.

### LC-MS/MS and bioinformatics.

LC-MS/MS and bioinformatics were performed as described (75) and in detailed in Supplemental Methods. The MS data have been deposited to the ProteomeXchange Consortium via the PRIDE partner repository with the data set identifier PXD035354.

### Statistics.

Statistical information is detailed in Supplemental Methods, with relevant statistical tests described in figure legends.

### Study approval.

The use of experimental animals is in agreement with a license issued by the Animal Experiments Inspectorate; Ministry of Food, Agriculture, and Fisheries; Danish Veterinary and Food Administration.

## Author contributions

RAF conceived the study. RL, SKM, SBP, PRG, AA, JRI, LC, and VM acquired data. RL, SKM, PRG, AA, JRI, LC, EJH, VM, PAW, and RAF analyzed and interpreted data. RL and RAF drafted the manuscript. All authors approved the final version of the manuscript and agree to be accountable for all aspects of the work.

## Supplementary Material

Supplemental data

Supplemental table 1

## Figures and Tables

**Table 1:**
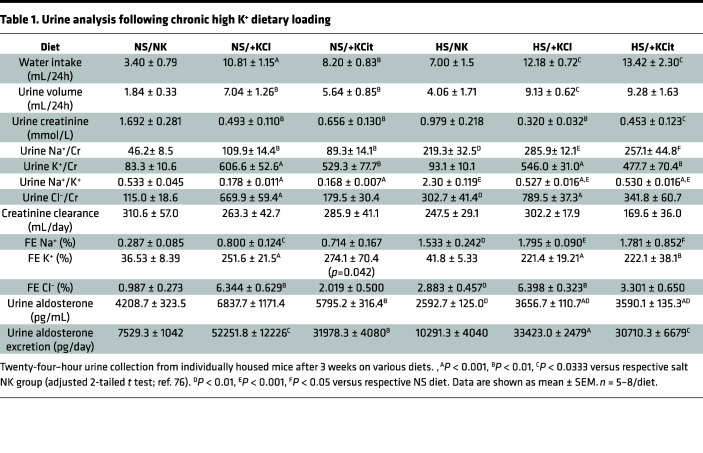
Urine analysis following chronic high K^+^ dietary loading

**Table 2:**
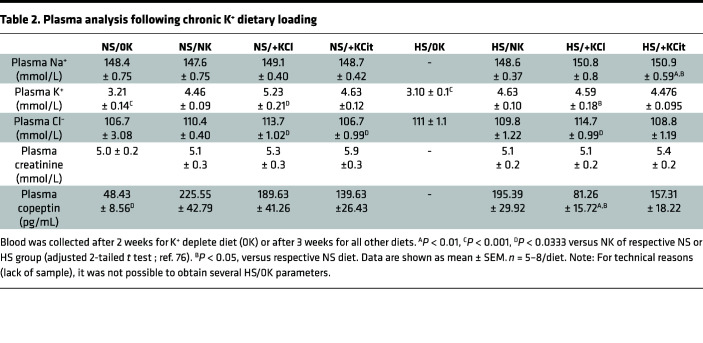
Plasma analysis following chronic K^+^ dietary loading

**Table 3:**
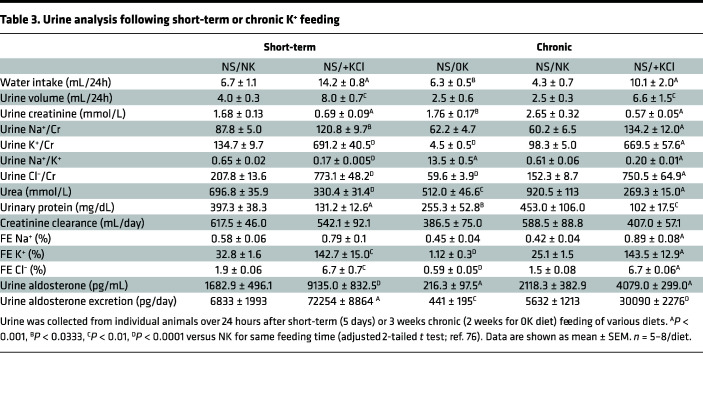
Urine analysis following short-term or chronic K^+^ feeding

**Table 4:**
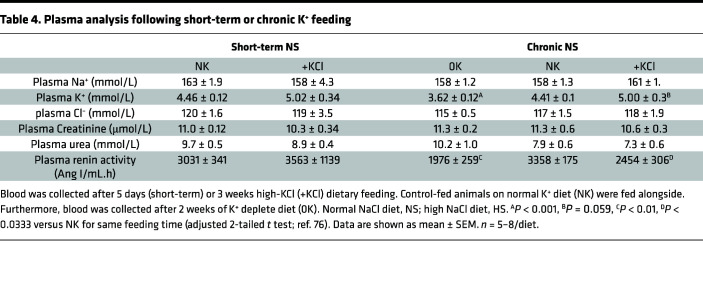
Plasma analysis following short-term or chronic K^+^ feeding

## Version 1 01/31/2023

In-Press Preview

## Version 2 03/08/2023

Electronic publication
