# Supplementary material for: Dissociation of sodium-chloride cotransporter expression and blood pressure during chronic high dietary potassium supplementation
Source: JCI Insight. 2023 Mar 8;8(5):e156437. doi: 10.1172/jci.insight.156437 (PMC10077486; doi:10.1172/jci.insight.156437)
Supplement: Supplemental data [file jciinsight-8-156437-s264.pdf]

Supplemental Material

Dissociation of Sodium-Chloride Cotransporter Expression and Blood Pressure During Chronic High Dietary Potassium Supplementation

Robert Little[1], Sathish K. Murali[1*], Søren B. Poulsen[1*], Paul R. Grimm[2], Adrienne Assmus[1], Lei Cheng[1], Jessica R. Ivy[3], Ewout J. Hoorn[4], Vladimir Matchkov[1], Paul A. Welling[2] and Robert A. Fenton[1]

[1]Department of Biomedicine, Aarhus University, Aarhus, Denmark

[2]Departments of Medicine, Nephrology and Physiology, Johns Hopkins School of Medicine, Baltimore, USA

[3]University/BHF Centre for Cardiovascular Science, The Queen's Medical Research Institute, The University of Edinburgh, Edinburgh, United Kingdom

[4]Erasmus Medical Center, University Medical Center Rotterdam, Rotterdam, the Netherlands

Contents

Extensive materials and methods 2

Supplemental Table 1. The effect of high salt and/or potassium deplete diet on blood pressure (BP) 8

Supplemental Table 2. The effect of chronic high potassium feeding on BP 9

Supplemental Table 3. Primary antibodies 11

Supplemental Figure 1. Telemetric recordings showed a consistently repeating pattern of systolic blood pressure (SBP). 12

Supplemental Figure 2. Vehicle injection has no significant effect on SBP 13

Supplemental Figure 3. High dietary K^+ intake has no significant effect on animals' activity 14

Supplemental Figure 4. NCC protein expression is not significantly altered following long-term high NaCl feeding. 15

Supplemental Figure 5. High K^+ diets and HS/0K feeding promote polyuria. 16

Supplemental Figure 6. BP increases after 1 week and then is further elevated after 3 weeks of high KCl feeding. 17

Supplemental Figure 7. Sirius red labelling of kidney sections shows no evidence for hypokalemic nephropathy. 18

Supplemental Figure 8. Immunohistochemical localization of NCC and γENaC after dietary K^+ manipulation. 19

Supplemental Figure 9. Western blot analysis of modulators of NCC and/or ENaC activity, or proteins important for Na+, K+ or water balance 21

Supplemental Figure 10. Correlations of NCC and ENaC with urine aldosterone concentration. 22

Supplemental Figure 11. Gene ontology (GO) analysis by ClueGO for proteins signficantly changed by short-term or chronic KCl feeding. 23

Supplemental Figure 12. Examples of proteins identified by mass spectrometry to have differential expression in the kidney cortex following high KCl feeding. 25

Supplemental table 4. Comparing chronic K^+ intake outcomes in current study to human population studies. 26

REFERENCES for supplemental 27

Extensive materials and methods

Animals and study design. All experimental protocols complied with the European Community guidelines for the use of experimental animals and were performed in agreement with a license issued by the Animal Experiments Inspectorate, Ministry of Food, Agriculture, and Fisheries, Danish Veterinary and Food Administration. Male C57Bl6/J mice (Janvier, France) of 10-12 weeks of age were housed under standard conditions with a 12/12 hour dark/light cycle (18:00 lights off) and continual free access to rodent chow and water. In an initial cohort (cohort 1), the animals were implanted with radiotelemetric devices (detailed below) to record their BP a week before any dietary manipulations commenced, during which they were individually caged. To limit potential telemetry probe malfunctions during mouse handling and prevent changes in BP due to volume depletion this group followed the same dietary interventions but their blood and urine was not sampled. Diets were prepared from powdered commercial rodent diet (Teklad Diet: TD.08251.Envigo. USA), being nominally K^+, Na^+ and Cl^- free, with ionic compounds (Sigma-Aldrich) added back to generate respective modified diets. 12 week old mice were fed a control diet of KCl (1.05% K^+) and NaCl (0.3% Na^+) (NK/NS) or a high NaCl (1.57% Na^+) (HS) diet for 7 weeks. Subsequently, mice were stratified to receive either a high KCl (5.25% K^+) or a high potassium citrate (KCit, 5.25% K^+) diet (HK) in combination with the control or high NaCl intake for 3 weeks. Following high K^+ feeding animals were fed a zero K^+ diet (0K) with either control or high NaCl level for 2 weeks. Animals maintained on HS/0K diet were treated with 37.5 mg/kg body weight hydrochlorothiazide (HCTZ, Sigma-Aldrich) administered as two i.p. doses 24 hours apart, with BP being recorded following the 2nd dose. In a subsequent experiment, animals fed a chronic NS/HK (high KCl) diet were treated with HCTZ (as above) and after a 3 day washout period received a single i.p. dose of amiloride (5 mg/kg body weight, Sigma-Aldrich) with BP being recorded for the first 12 hours following injection. HCTZ and amiloride were prepared from solid by dilution in DMSO and then further diluted in sterile physiological saline solution before injection at the stated doses. A scheme of the diets and study outline is shown in ***Figure 1***.

Telemetric Blood Pressure Measurements. BP recordings and associated surgery was performed using PA-C10 and HDX-11 radiotelemetry devices (Data Sciences International (DSI), USA) as described previously (1, 2). *In vivo* BP was recorded using Ponemah software (ver. 6.4, DSI) (two 1 minute recordings every 5 minutes). BP data was collected in a session of up to 3 days and the average BP for each hour was calculated. Where recordings were collected over multiple days, values from matched times of day were averaged and BP values are presented over a 24 hour period from 18:00 (ZT hour 0), the time at which lights in the animal facility automatically switch off. A four factor fixed-effects single-component model was then applied to plot the rhythmicity of the BP using cosinor curve fitting (1, 3). Curves were generated from the following equation:

Y = mesor+amplitude*cos(period*(X-acrophase)).

No default constraints were defined for curve fitting. Initial values were: mesor = 1 (rule = *YMID), amplitude = 0.5 (rule = *YMAX-YMIN), period = 0.268 (rule = initial value, to be fit), acrophase = 1 *(Value of X at YMAX). The effect of diets on BP was analysed by comparison of fits analysis for the curves. The derived Midline Estimated Statistic Of Rhythm (MESOR) value is presented as the mean BP across a 24 hour period.

Tail cuff plethysmography. In a subsequent cohort of animals (cohort 2) conscious BP was recorded from the tail using an occlusion cuff and volume-pressure recording (VPR) sensor (Coda equipment, Kent Scientific). Animals were progressively acclimatised to the restraint and cuffing system over 3 days as previously described (4, 5). VPR traces were obtained from individual animals sequentially not alongside each other, as in our experience animals from multiple channels don't look as relaxed and generally data collected in lower throughput has less variation between cycles. Recording settings; maximal cuffing pressure of 250mmHg, occlusion cuff deflation over 15 seconds for 1 cycle, 5 seconds inter cycle interval. An initial 5 acclimatisation cycles were programmed, followed by up to 15 further repeating cycles where

accepted values (tail volume > 15µL from calm animal) could be used for analysis. Individual cycles were interrogated by the researcher to discard potential movement artefacts. The mean from a minimum of 5 accepted cycles was calculated and is presented as the BP value of an individual for SBP or DBP. All recordings were performed during the dark phase, approximately 19:00 – 22:00, in a blackout room under a single red light. BP was recorded from NK and HK fed animals in the same session. Animals being recorded from were moved to the recording room from a housing room during the light phase and had at least 6 hours acclimatisation to the area. In using this system to assess effects of HCTZ on BP, animals were briefly anesthetised by vaporised isoflurane and HCTZ injected (for 37.5mg/kg body weight) at least 4 hours before recordings were initiated.

Physiological phenotyping. Dietary effects on renal water and solute handling were determined by individually housing animals in metabolic cages (Tecniplast, Italy). Mice had *ad libitum* access to water while fed the different diets as detailed in the 'study design' section. Animals were acclimatised to the cage for 2 days, after which urine was collected over a 24-hour period, starting at 09:00, and for the same period the volume of water drank and food eaten was determined. Urine was centrifuged at 1000 *g* for 10 min before storage at -20°C until required.

Plasma and urine analysis. Animals were anaesthetised under 5% isoflurane for 60 s, Li-heparin capillaries inserted into the retro orbital plexus, and blood collected directly into Li-heparin coated tubes. Blood was immediately centrifuged at 5000 *g* for 2 minutes and the upper plasma layer collected and flash frozen. Samples were stored at -20°C until required. Plasma and 24-hour urine samples were analysed for K^+, Na^+, Cl^- and creatinine by the Clinical Pathology Laboratory at the Medical Research Council (Harwell, Oxfordshire, United Kingdom). Determination of ions was by indirect ISE, and creatinine levels determined by an enzymatic method, with samples run on a Beckman Coulter AU680 clinical chemistry analyser. Aldosterone concentrations were determined using an enzyme immunoassay kit (EIA-5298; range: 20-

1,000 pg/ml; DRG International, Springfield, NJ) as per the manufacturer's protocol. Plasma renin activity was determined with an in-house kinetic assay (Erasmus Medical Center, Rotterdam, the Netherlands) as previously described (6) with detection limit = 0.17 ng Ang I/mL per hour. Plasma copeptin (7) was determined using a mouse specific ELISA (CEA365, Cloud-Clone Corporation).

Immunoblotting. Animals were euthanized by cervical dislocation, kidneys removed and protein homogenates prepared as previously described (1). Standard procedures were utilized for SDS-PAGE using 4–15% gradient polyacrylamide gels (Criterion TGX Precast Protein Gels, BioRad). Equal quantities of total protein were loaded per lane as determined by Coomassie blue staining. The maximal deviations in total protein concentration between samples on individual blots were ± 10%. Primary antibodies used for immunoblotting are listed in ***Supplemental Table 3***. All antibodies have been extensively characterized in previous studies. Secondary antibodies (1:5000, Dako) were incubated for 1 hour at room temperature before immunoblots were visualised using the Enhanced Chemiluminescence system (GE Healthcare) or SuperSignal West Femto Chemiluminescent Substrate (Thermo Scientific) by an ImageQuant LAS 4000 imager (GE Healthcare). Signal intensity in specific bands were quantified using Image Studio Lite (Qiagen) densitometry analysis.

Real-time quantitative PCR (RT-qPCR). Animals were maintained on a similar control diet to the main study (0.25% Na^+, 0.67% K^+ - Rodent Maintenance diet 1, Special Diet Services) under standard facility conditions with 12 h dark/light cycle. Whole kidney was snap frozen at ZT time 6 hours (13:00 hours – light phase) or 12 hours later at ZT 18 hours (01:00 – dark phase). RT-qPCR was performed as described (8) for *Scnn1a (*encoding αENaC) using the Roche Universal Probe Library. *Scnn1a* mRNA level was normalised to the mean expression of 3 control genes; *18S*, *cyclophilin A* and *TBP* and data standardised to show the relative fold change in mRNA level at 01:00 compared to 13:00.

Immunohistochemistry. Half a kidney was immersion fixed in 4% paraformaldehyde (PFA) for 24 h at 4°C. Tissue preparation, sectioning and immunolabeling was as described using primary antibodies targeting NCC, pNCC, γENaC or proliferating cell nuclear antigen (PCNA) and HRP conjugated secondary antibodies (Dako). Sections were visualised using a slide scanner system (Slide Scanner Olympus VS120 using a × 40 objective). For cell proliferation, cells were classed as PCNA positive (PNCA+) if there was clearly visible labelling of the nuclei. The number of PNCA+ cells were counted in 5 non-overlapping fields of view (each 0.31mm^2) across a section and the data reported as PCNA+/1.55mm^2 for each animal.

Liquid Chromatography Mass Spectrometry (LC-MS/MS) and Bioinformatics. 150 µg of mouse kidney cortex homogenates were solubilized, digested to peptides, and quantified as described (9). Peptide samples were labelled individually using the TMTpro™ Label Reagent (Thermo Scientific), fractionated using high pH fractionation and analyzed by LC-MS/MS using an Easy nLC-1200 coupled to a Tribrid Fusion mass spectrometer (Thermo Scientific) for protein identification and quantification as described (10). Raw data were searched using SEQUEST and MASCOT against the reviewed uniprot mouse protein database (dated 13-01-2022) and quantified using Proteome Discoverer, version 2.4 (Thermo Scientific). The parameters for Proteome Discoverer were: precursor mass tolerance, 10 ppm; fragment mass tolerance, 0.02 Da; maximum miss cleavage, 2; static modification: cysteine carbamidomethylation, TMTpro modification on peptide N-terminal and lysine; variable modification: N-terminal acetylation, methionine oxidation. Percolator was used to calculate false discovery rate (FDR). Only rank 1 and high confidence (with a target false discovery rate (FDR) q-value below 0.01) peptides were included in the results. Protein quantification was based on normalized relative TMT reporter ion intensities. Only unique peptides were used for quantification. Proteins with a quantification P value lower than 0.05 were considered significantly changed and subjected to further downstream analysis. The mass spectrometry data have been deposited to the ProteomeXchange Consortium via the PRIDE partner repository with the dataset identifier PXD035354 (Username: reviewer_pxd035354@ebi.ac.uk, Password: dT1rUiz5). For gene

ontology (GO) analysis, data were analyzed using Cytoscape with ClueGO Plugin version 2.5.9, using all without IEA as evidence. Related terms that share similar associated genes were fused to reduce redundancy. $P < 0.05$ was considered significant. Ingenuity pathway analysis (IPA, 2022 release) was performed using proteins with significantly changed abundances and the Ingenuity Knowledge Database (gene only) was used as the reference set for analysis.

Data processing and statistical analysis. Data was curated in Microsoft Excel. A 4 factor cosine curve was fitted to the expected regularly repeating pattern of BP, as previously described (1). Datasets were further analysed using Graph Pad Prism v9.1. For comparison of two groups, data meeting the statistical assumptions of normality were assessed using an unpaired Students *t*-test with level of significance set as 0.05. Comparisons of more than two groups were performed using one- or two-way (regular or repeated measurement) ANOVAs followed by a Dunnett or Tukey multiple comparison test (see individual figure legends). An exception was the analysis of urine and plasma data where individual feeding groups, from a dataset of three or more, were compared to the control group using an unpaired Students *t*-test. In this instance the level of significance was set as 0.033 to correct for the false discovery rate (FDR) (11). Data is plotted as mean ± standard error (SEM) alongside individual values from independent animals, unless otherwise stated.

Study approval. The use of experimental animals is in agreement with a license issued by the Animal Experiments Inspectorate, Ministry of Food, Agriculture, and Fisheries, Danish Veterinary and Food Administration.

Supplemental Table 1. The effect of high salt and/or potassium deplete diet on blood pressure (BP). Diet key: NS = normal NaCl (0.3% Na^+), HS = high NaCl (1.57% Na^+), NK = normal KCl (1.05% K^+), 0K = zero K^+ diet. BP values obtained from implanted telemetry devices after 7 weeks of NS/NK or HS/NK diet, or 2 weeks of NS/0K or HS/0K diet. Pulse pressure (PP) was determined as SBP – DBP at each hour and then averaged. *P<0.05, **P<0.01, ***P<0.001 effect of HS Vs NS. #P<0.05, ##P<0.001 effect of 0K Vs NK. 2-Way ANOVA with Dunnett's multiple comparison test. Values are Mean ± SEM.

24 hour BP

Diet	SBP (mmHg)	DBP (mmHg)	MAP (mmHg)	PP (mmHg)	N animals
NS/NK	115.6 ± 1.3	88.6 ± 1.3	102.4 ± 1.2	27.6 ± 2.9	6
HS/NK	126.0 ± 1.2***	90.8 ± 1.9	108.5 ± 1.5**	34.1 ± 1.4	6
NS/0K	123.3 ± 3.4#	99.1 ± 2.0##	111.4 ± 2.0##	24.2 ± 3.9	5
HS/0K	132.5 ± 2.7***, #	101.1 ± 6.1	118.0 ± 5.3#	31.4 ± 3.9	6

12 hours night period BP

Diet	SBP (mmHg)	DBP (mmHg)	MAP (mmHg)	PP (mmHg)	N animals
NS/NK	124.1 ± 1.6	95.8 ± 2.3	110.4 ± 1.5	29.5 ± 2.8	6
HS/NK	129.8 ± 2.5	103.2 ± 3.0	109.5 ± 3.1	39.5 ± 4.7	6
NS/0K	130.9 ± 4.3	105.0 ± 2.5#	118.2 ± 3.0#	25.9 ± 3.9	5
HS/0K	142.9 ± 2.6***, #	109.8 ± 6.2	128.0 ± 5.3*##	33.1 ± 4.8	6

12 hours light period BP

Diet	SBP (mmHg)	DBP (mmHg)	MAP (mmHg)	PP (mmHg)	N animals
NS/NK	107.0 ± 1.1	80.0 ± 1.33	94.0 ± 0.5	27.1 ± 2.1	6
HS/NK	122.0 ± 1.7***	90.0 ± 2.9*	107.3 ± 2.7**	28.7 ± 5.4	6
NS/0K	115.0 ± 3.0*	92.6 ± 2.9**	104.0 ± 2.0##	22.4 ± 4.0	5
HS/0K	121.1 ± 3.6**	91.6 ± 6.1	107.1 ± 5.3	29.5 ± 2.9	6

Supplemental Table 2. The effect of chronic high potassium feeding on BP. BP values obtained from implanted telemetry devices. Diet key: NS = normal NaCl (0.3% Na^+), HS = high NaCl (1.57% Na^+), NK = normal KCl (1.05% K^+), +KCl= high KCl (5.25% K^+), +KCit = high K citrate (5.25% K^+) diet. Animals were fed a normal (NS/NK) or high (HS/NK) NaCl diet for 7 weeks. They were then stratified to continue on this diet, or receive a diet supplemented with KCl (NS/+KCl or HS/+KCl) or high potassium citrate (NS/+KCit or HS/+KCit) for 3 weeks. Pulse pressure (PP) was determined as SBP – DBP at each hour and then averaged. *P<0.05, **P<0.01, ***P<0.001 vs respective NK (NS or HS). #P<0.05 effect of HS diet vs respective NS diet. 2-Way ANOVA with Dunnett's multiple comparison test. Values are Mean ± SEM.

<u>24-hour BP</u>

Diet	SBP (mmHg)	DBP (mmHg)	MAP (mmHg)	Pulse pressure (mmHg)	N animals
NS/NK	123.5 ± 1.3	96.2 ± 2.4	110.7 ± 1.2	28.1 ± 2.7	11
NS/+KCl	128.4 ± 1.9*	100.2 ± 5.1	114.8 ± 2.9	26.8 ± 4.4	5
NS/+KCit	130.5 ± 2.8**	99.0 ± 3.9	115.1 ± 2.8	31.8 ± 3.6	5
HS/NK	128.9 ± 1.5	93.1 ± 1.5	110.7 ± 1.6	35.2 ± 0.9	6
HS/+KCl	128.7 ± 1.6	94.3 ± 1.7	112.3 ± 1.2	35.7 ± 3.7	3
HS/+KCit	129.7 ± 2.7	93.8 ± 1.8	110.1 ± 1.9	33.7 ± 1.9	3

<u>12-hour night BP</u>

Diet	SBP (mmHg)	DBP (mmHg)	MAP (mmHg)	Pulse pressure (mmHg)	N animals
NS/NK	129.0 ± 1.9	100.6 ± 2.6	115.5 ± 1.9	29.1 ± 2.9	11
NS/+KCl	140.0 ± 2.5**	108.9 ± 5.1	124.9 ± 3.0*	30.1 ± 5.1	5
NS/+KCit	139.4 ± 3.2**	106.3 ± 3.8	123.2 ± 3.0*	34.7 ± 3.7	5
HS/NK	133.7 ± 2.5	94.1 ± 3.1	114.4 ± 3.0	39.6 ± 2.7#	6
HS/+KCl	140.1 ± 3.3	103.5 ± 2.3	122.4 ± 2.5*	39.5 ± 5.8	3
HS/+KCit	138.3 ± 1.3	102.7 ± 0.9	120.3 ± 1.4	35.7 ± 4.5	3

12-hour light BP

Diet	SBP (mmHg)	DBP (mmHg)	MAP (mmHg)	Pulse pressure (mmHg)	N animals
NS/NK	118.3 ± 1.1	91.7 ± 2.7	106.0 ± 1.6	27.0 ± 2.6	11
NS/+KCl	116.2 ± 1.7	91.4 ± 5.3	104.4 ± 3.3	23.5 ± 3.8	5
NS/+KCit	121.2 ± 2.6	91.5 ± 4.0	106.7 ± 2.8	28.9 ± 3.6	5
HS/NK	122.6 ± 2.1	91.1 ± 3.8	107.0 ± 2.5	30.8 ± 3.4	6
HS/+KCl	117.3 ± 0.3*	85.4 ± 2.0	102.2 ± 0.9	31.8 ± 2.0	3
HS/+KCit	121.0 ± 5.8	84.8 ± 2.7	99.8 ± 5.2	31.6 ± 0.7	3

Supplemental Table 3. Primary antibodies

Protein	Antibody	Supplier	Molecular weight (KDa)	Reference
NCC	SPC-402	StressMarq	~100 and 150	Used and validated in: (12)
pNCC (T58)	1251	Self-made	~ 150	(12)
Proteasome 20S	sc-67339	Santa Cruz	20	
αENaC		Gift from Johannes Loffing	90 (total)<br>25 (cleaved)	(13)
γENaC		Gift from Mark Knepper	85 (total)<br>25 (cleaved)	(14)
Parvalbumin	PV235	Swant	10	
Calbindin D28	10R-C106a	Fitzgerald	30	
HATPase	7659	Self-made	60	(15)
pWNK4		Gift from David Ellison	160	(16)
WNK4		Gift from David Ellison	160	(17)
SPAK		Gift from Hiroshi Shibuya	65	(12)
Kir 5.1	LS-C177333	LSBio	150	
ROMK	NBP1-82874	Novus	45	
Pendrin	RA2671	Self-made	110	(18)
NKCC2	L320	Gift from Mark Knepper	150	(19)
NHE3	LL546	Gift from Mark Knepper	75	(19)
AQP2	AQP2 9398	Self made	25 and 35-45	(20)

Supplemental Figure 1. Telemetric recordings showed a consistently repeating pattern of systolic blood pressure (SBP). SBP was recorded over 3 days showing a consistent repeating pattern. Time of darkness for animals is shown by black boxes above the x-axis. Mean ± SEM for hourly averaged BP. n=6.

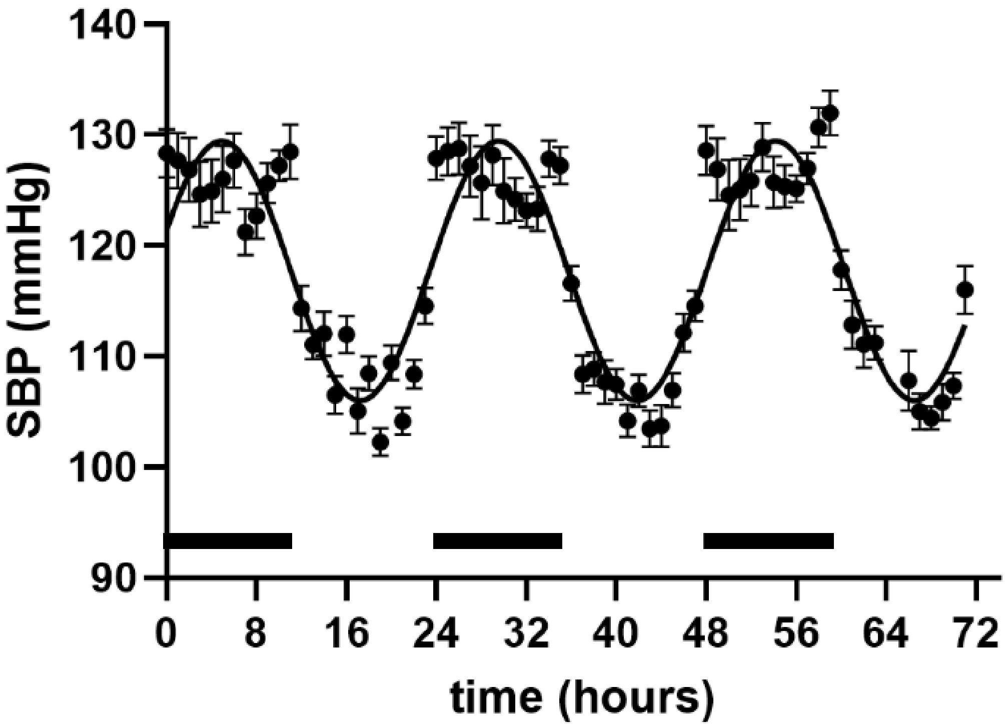

Supplemental Figure 2. Vehicle injection has no significant effect on SBP. Telemetric BP recording over 24 hours. Diet key: NS = normal NaCl (0.3% Na^+), +KCl= high KCl (5.25% K^+). Animals fed NS/+KCl for 3 weeks. ZT 0 is 18:00 hours when lighting turned off in animal area. Black and white bar above axis represents time animals in darkness and light, respectively. Mean ± SEM for hourly averaged BP. n=4-5/treatment. Comparison of fits test.

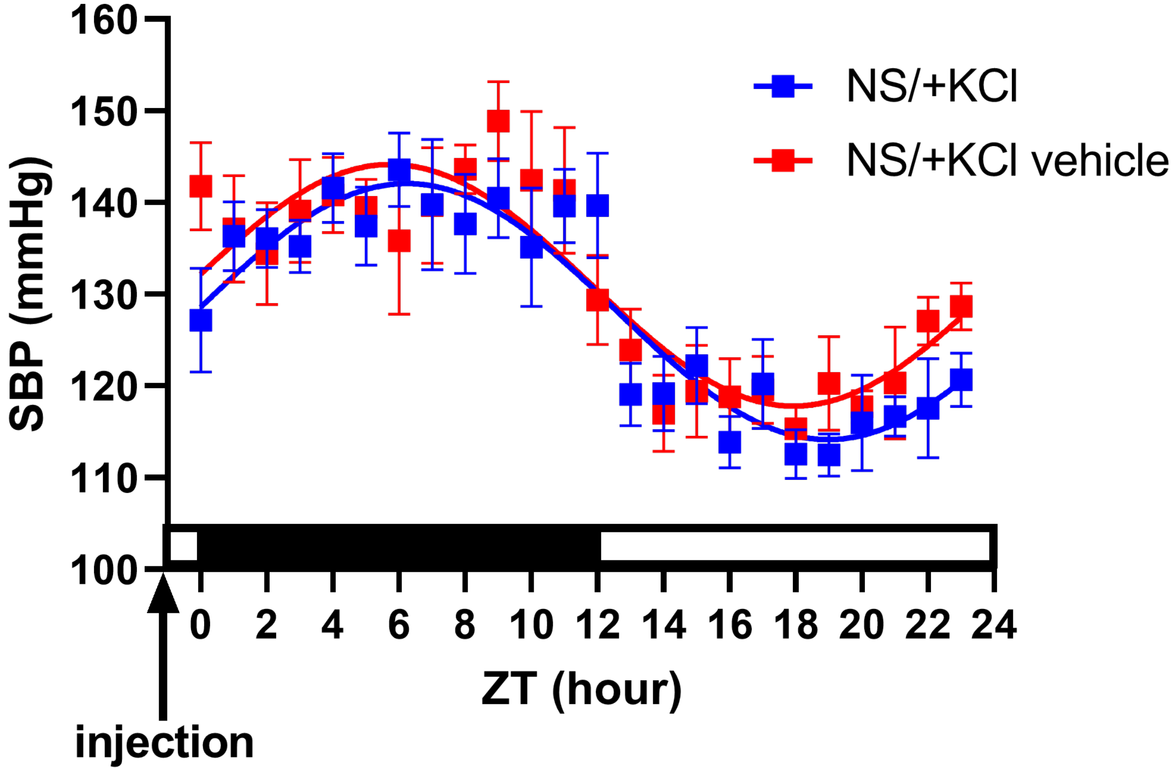

Supplemental Figure 3. High dietary K^+ intake has no significant effect on animals' activity. Diet key: NS = normal NaCl (0.3% Na^+), HS = high NaCl (1.57% Na^+), NK = normal KCl (1.05% K^+), +KCl= high KCl (5.25% K^+), +KCit = high K citrate (5.25% K^+). Following 3 weeks feeding of NS/+KCl or NS/+KCit diets animals' activity was similar to those fed a NS/NK diet. **A**. Activity measurements by telemetry over 24 hours on a normal NaCl (NS) diet. Time of darkness for animals is shown by black boxes below the x-axis. **B**. Stratification of activity measurements to night or day. **C**. Activity measurements over 24 hours on a high salt (HS) diet. Time of darkness for animals is shown by black boxes below the graph. **D**. Stratification of activity measurements to night or day. Data is shown as mean ± SEM with individual data points representing different animals. Individuals shown in **B** and **D** make up the mean value represented in **A.** and **C**. *P<0.05, **P<0.01. Testing by 1 way ANOVA and Dunnett's multiple comparison test.

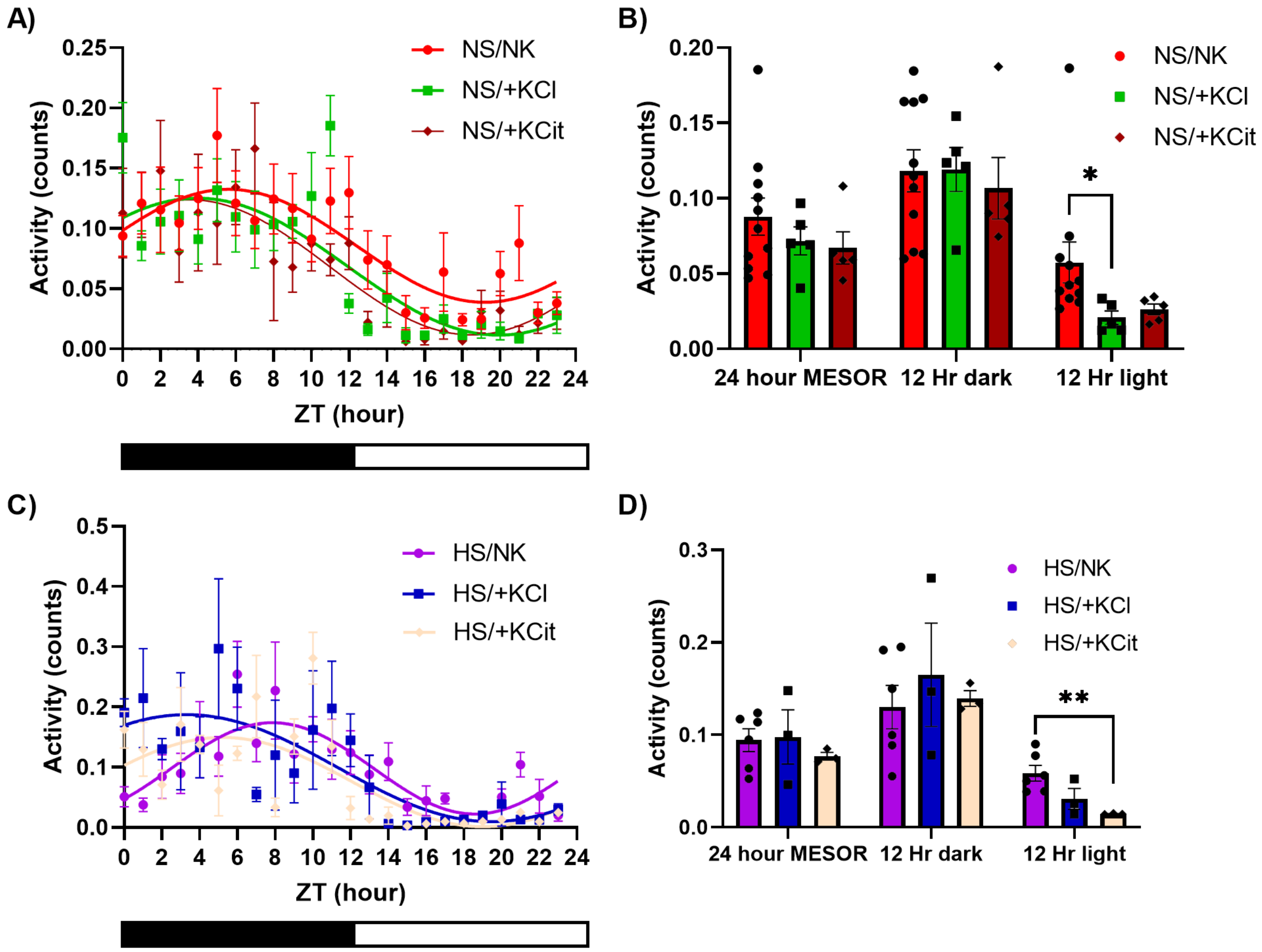

Supplemental Figure 4. NCC protein expression is not significantly altered following long-term high NaCl feeding. Diet key: NS = normal NaCl (0.3% Na^+), HS = high NaCl (1.57% Na^+), NK = normal KCl (1.05% K^+). Representative panels (samples run on the same gel but non-contiguous) of NCC and pNCC protein expression in whole kidney by western blotting are shown above quantification graphs. Neither NCC or pNCC protein was significantly altered by long term (3 weeks) high NaCl (HS) feeding compared to animals fed control NaCl (NS) diets. Data is shown as mean ± SEM with individual data points representing different animals. Statistical testing by students 2-way t-test was performed.

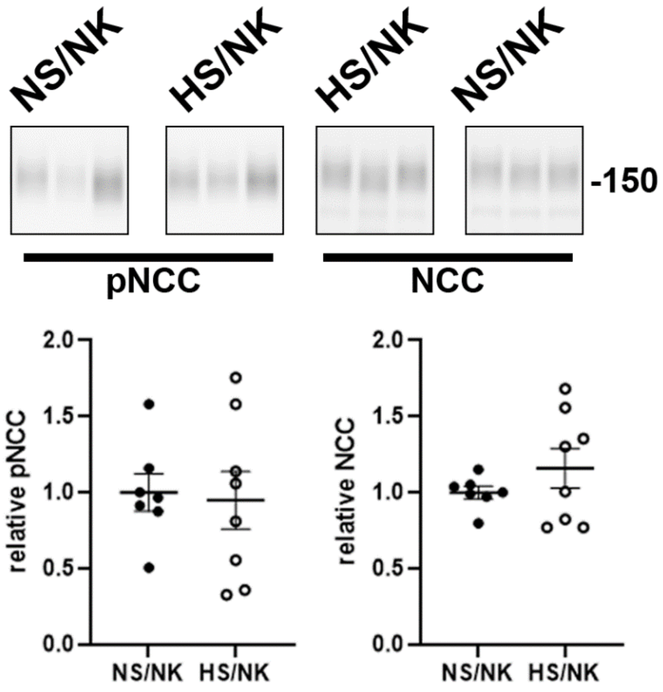

Supplemental Figure 5. High K⁺ diets and HS/0K feeding promote polyuria. Diet key: NS = normal NaCl (0.3% Na^+), HS = high NaCl (1.57% Na^+), NK = normal KCl (1.05% K^+), +KCl= high KCl (5.25% K^+), +KCit = high K citrate (5.25% K^+), 0K = zero K^+ diet. Chronic feeding (3 weeks) of NK/+KCl or NK/+KCit diets increased 24 h urine volume relative to those on a NS/NK diet. Short-term (4 days) of NS/+KCl also significantly increased urine output. Chronic feeding (3 weeks) of HS/+KCl or HS/+KCit diets, or 2 weeks of a HS/0K diet increased 24 h urine volume relative to those on a HS/NK diet. Data is shown as mean ± SEM with individual data points representing different animals. 1 way ANOVA and Dunnett's multiple comparison test where ≥ 3 columns for a condition. *P<0.05. **P<0.01 ****P<0.0001.

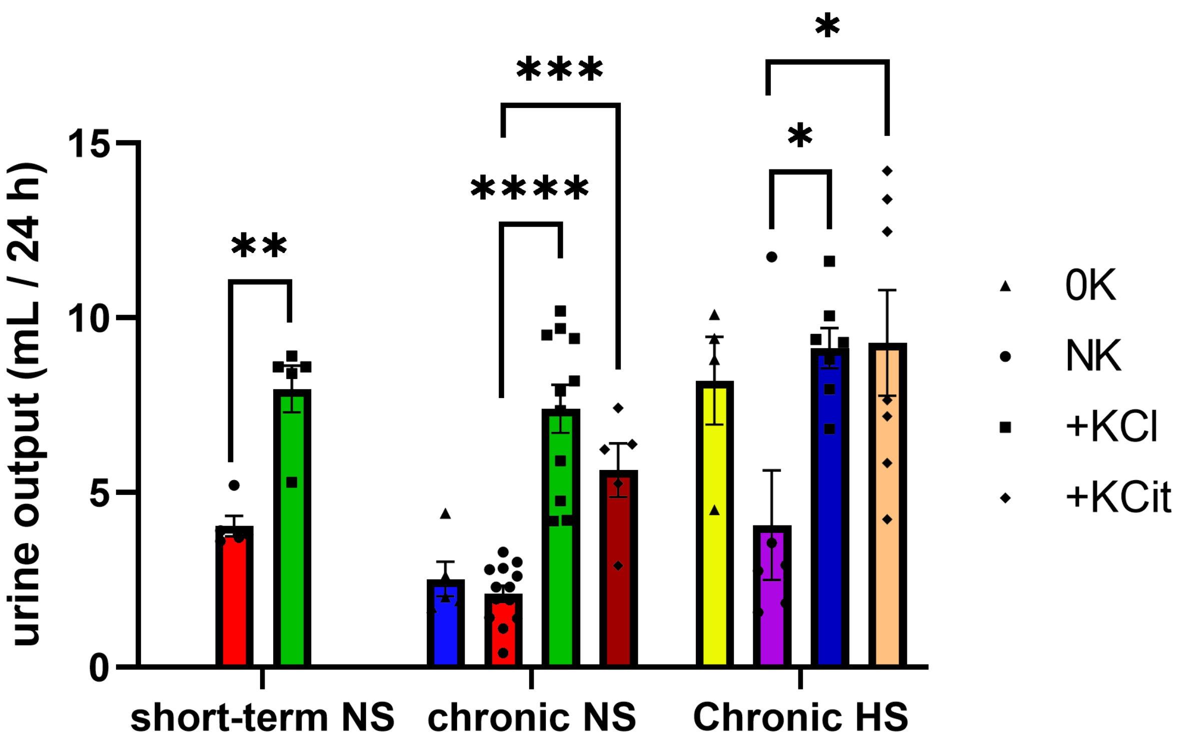

Supplemental Figure 6. BP increases after 1 week and then is further elevated after 3 weeks of high KCl feeding. Diet key: NS = normal NaCl (0.3% Na^+), NK = normal KCl (1.05% K^+), +KCl= high KCl (5.25% K^+), +KCit = high K citrate (5.25% K^+). Telemetric SBP recording over 24 hours for NS/+KCl fed animals was increased during dark period after 1 week and 3 weeks feeding. **A**. 24 hours recording, **B**. quantification of full recording and stratified by dark and light periods. Dark period was 12 hours from ZT hour 0 (18:00) as shown by bar above x axis. SBP recording over 24 hours for NS/+KCit fed animals was increased during dark period after 1 week and 3 weeks feeding; **C**. 24 hours recording, **D**. quantification of full recording and stratified by dark and light periods). Data is shown as mean ± SEM with individual data points representing different animals. Individuals shown in **B** and **D** make up the mean value represented in **A** and **C**. *P<0.05, **P<0.01. Data between groups is analysed by T-test corrected for false discovery rate.

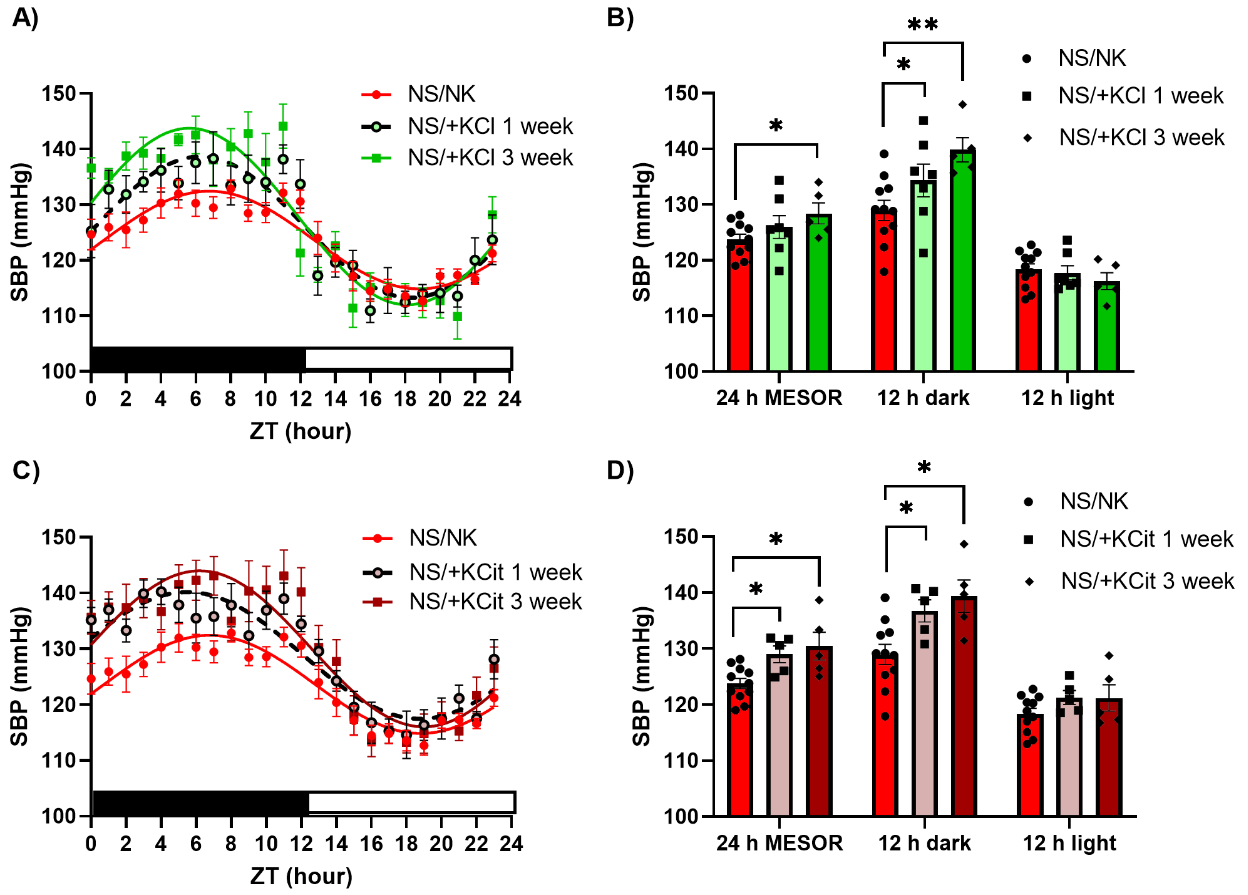

Supplemental Figure 7. Sirius red labelling of kidney sections shows no evidence for hypokalemic nephropathy. Diet key: NS = normal NaCl (0.3% Na^{+}), NK = normal KCl (1.05% K^{+}), HK= high KCl (5.25% K^{+}), 0K = zero K^{+} diet for 2 weeks. Short and chronic HK feeding was for 4 days and 3 weeks respectively**.** Representative images are shown from comparable sectional depths of paraffin embedded whole kidney tissue. Images from ≥5 animals/dietary condition were collected and compared. Mean ± SEM. Data compared using 1-way ANOVA with Tukeys multiple comparisons test.

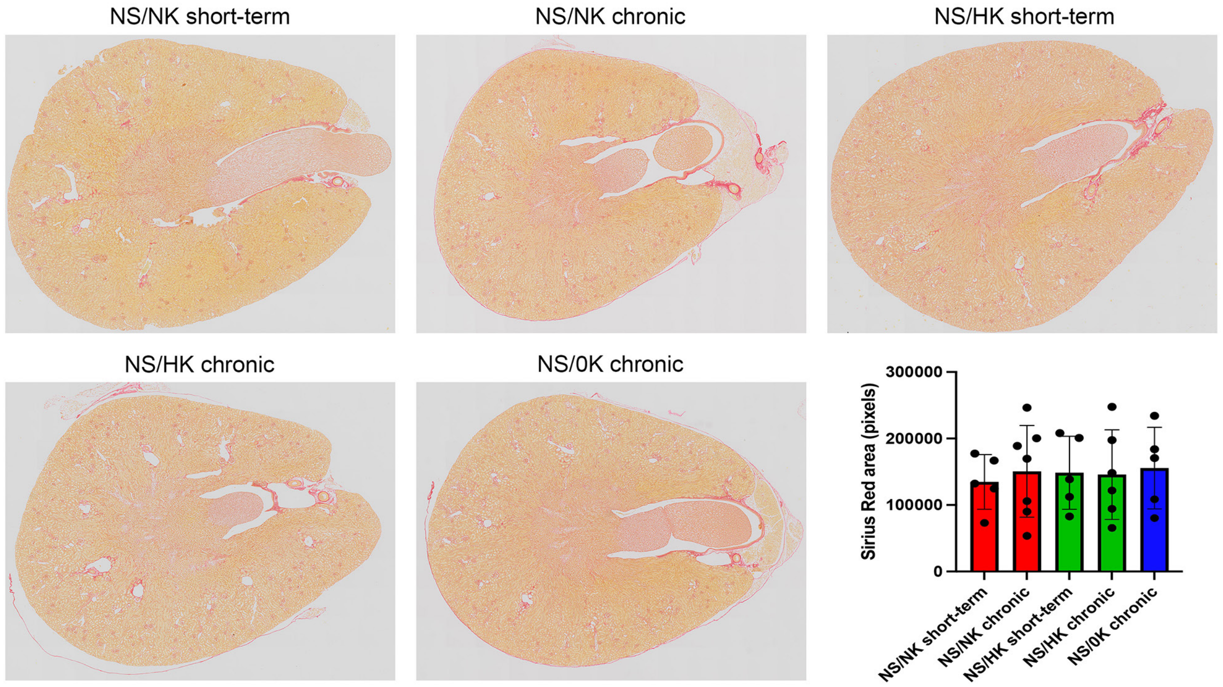

Supplemental Figure 8. Immunohistochemical localization of NCC and γENaC after dietary K^+ manipulation. Diet key: NS = normal NaCl (0.3% Na^+), NK = normal KCl (1.05% K^+), HK= high KCl (5.25% K^+), 0K = zero K^+ diet. Representative images are shown from comparable sectional depths of paraffin embedded whole kidney tissue. Images from ≥5 animals/dietary condition were collected and compared. After 2 weeks of NS/0K diet, qualitative staining intensity of NCC and pNCC in the kidney is greatly increased relative to NS/NK intake, whereas staining intensity is decreased after feeding of a NS/+KCl diet (NS/HK) short-term (4 days) or chronically (3 weeks). After feeding a NS/OK or NS/NK diet γENaC labelling is predominantly intracellular, whereas after short-term NS/+KCl feeding it is both intracellular and located in the apical plasma membrane domain. On a chronic NS/HK diet γENaC is predominantly observed in the apical membrane domain. Scale bar represents 500µm.

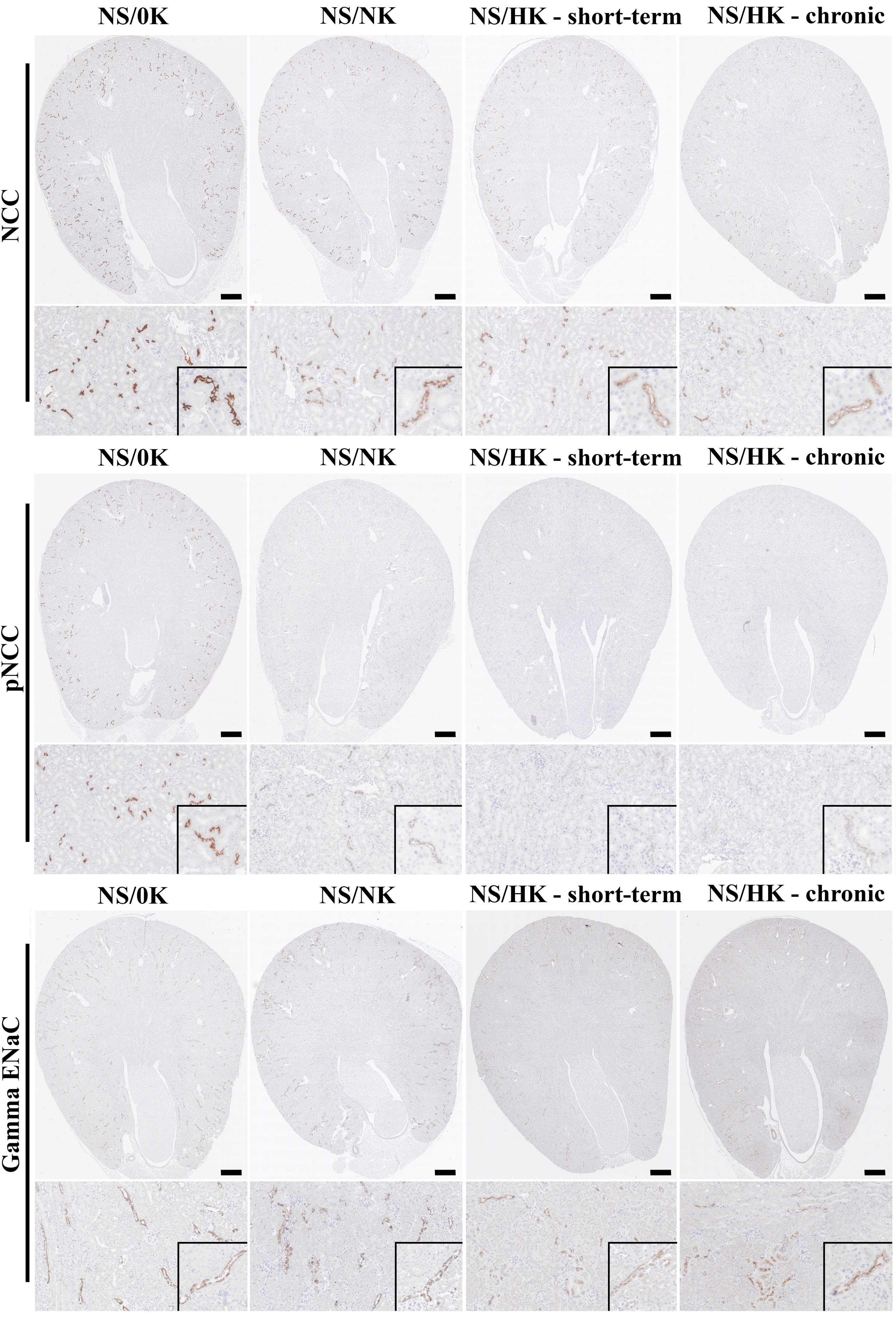
NS/0K
NS/NK
NS/HK - short-term
NS/HK - chronic
NCC
pNCC
Gamma ENaC

Supplemental Figure 9. Western blot analysis of modulators of NCC and/or ENaC activity, or proteins important for Na+, K+ or water balance. Diet key: NS = normal NaCl (0.3% Na^+), NK = normal KCl (1.05% K^+), +KCl= high KCl (5.25% K^+), 0K = zero K^+ diet. Short term feeding was for 4 days, chronic NS/NK and NS/+KCl feeding was for 3 weeks, whereas NS/0K was for 2 weeks. Panels (samples run on the same gel but non-contiguous) from representative blots are shown. A) Phosphorylated (active) With-No-Lysine 4 (pWNK4). B) WNK4. C) SPS1-related proline/alanine-rich kinase (SPAK). D) Inwardly rectifying K^+ channel Kir 5.1. E) Renal outer medullary potassium channel (ROMK). F) The Cl^-/$HCO3^-$ exchanger pendrin. G) Na-K-2Cl cotransporter NKCC2. H) The Na^+/H^+ exchanger NHE3. I) Water channel AQP2. Data is shown as mean ± SEM with individual data points representing different animals. Data compared using 1-way ANOVA with Tukeys multiple comparisons test. *P<0.05.

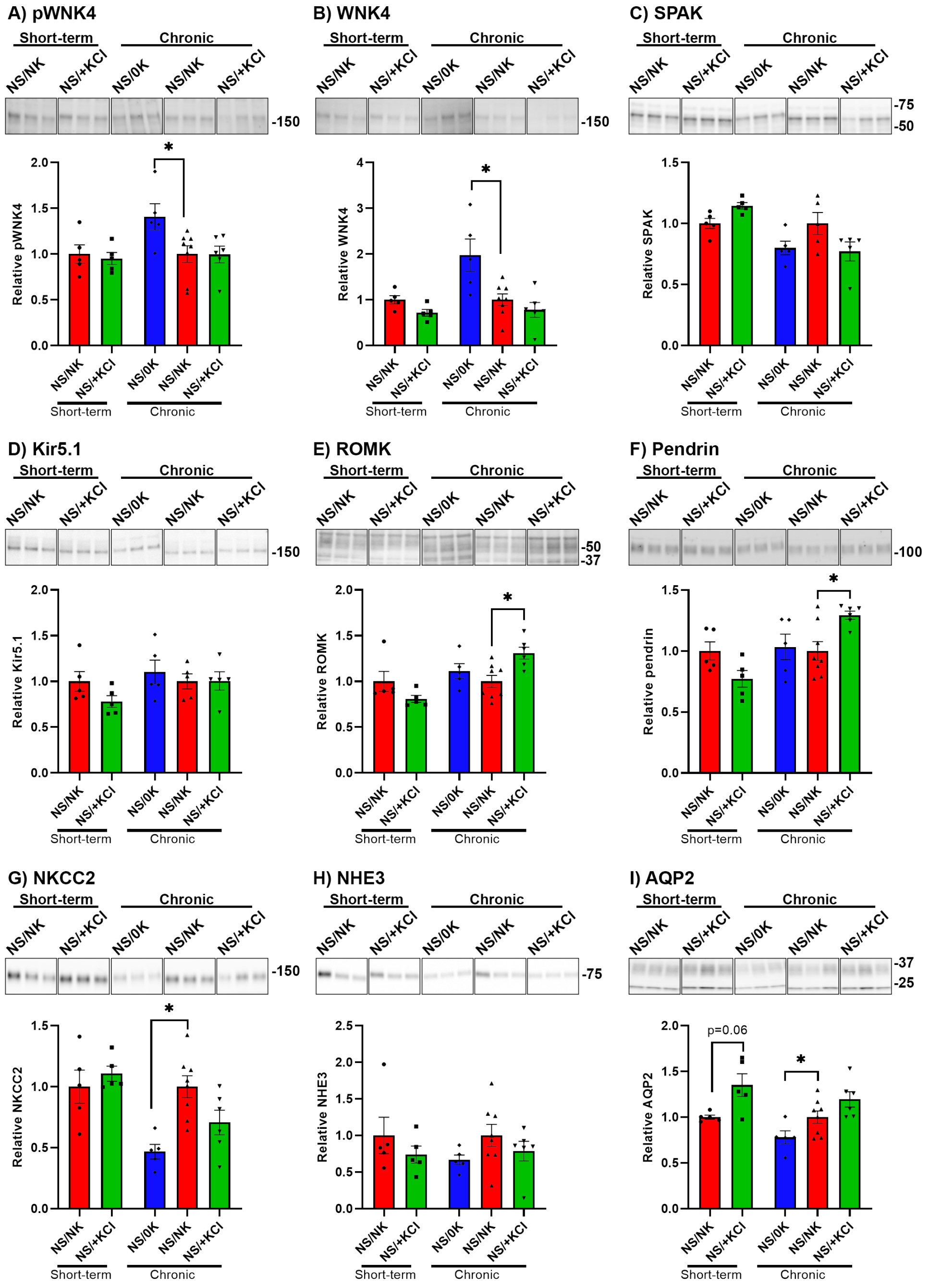

Supplemental Figure 10. Correlations of NCC and ENaC with urine aldosterone concentration. Diet key: NS = normal NaCl (0.3% Na^+), HS = high NaCl (1.57% Na^+), NK = normal KCl (1.05% K^+), +KCl= high KCl (5.25% K^+), +KCit = high K citrate (5.25% K^+), 0K = zero K^+ diet. **A.** Total NCC protein significantly negatively correlates with the urine [aldosterone]. **B**. total αENaC trends to positively correlate. For panel A and B, animals were fed NS/NK, HS/NK, NS/+KCl, HS/+KCl, NS/+KCit or HS/+KCit for 3 weeks, or NS/0K and HS/0K for 2 weeks. As the correlation between total NCC or total αENaC and urine [aldosterone] did not significantly change with diet, all dietary conditions are plotted in combined figures. **C**. Total γENaC significantly negatively correlates with urine [aldosterone], an effect which is more significant following chronic NS/+KCl feeding (short-term *p*=0.0015, chronic *p*=0.0004). Animals were fed a NS/NK or NS/+KCl diet for 4 days (short-term) or 3 weeks (chronic). Protein expression was determined by western blotting of whole kidney samples. Individual data points represent different animals and line shows linear regression analysis.

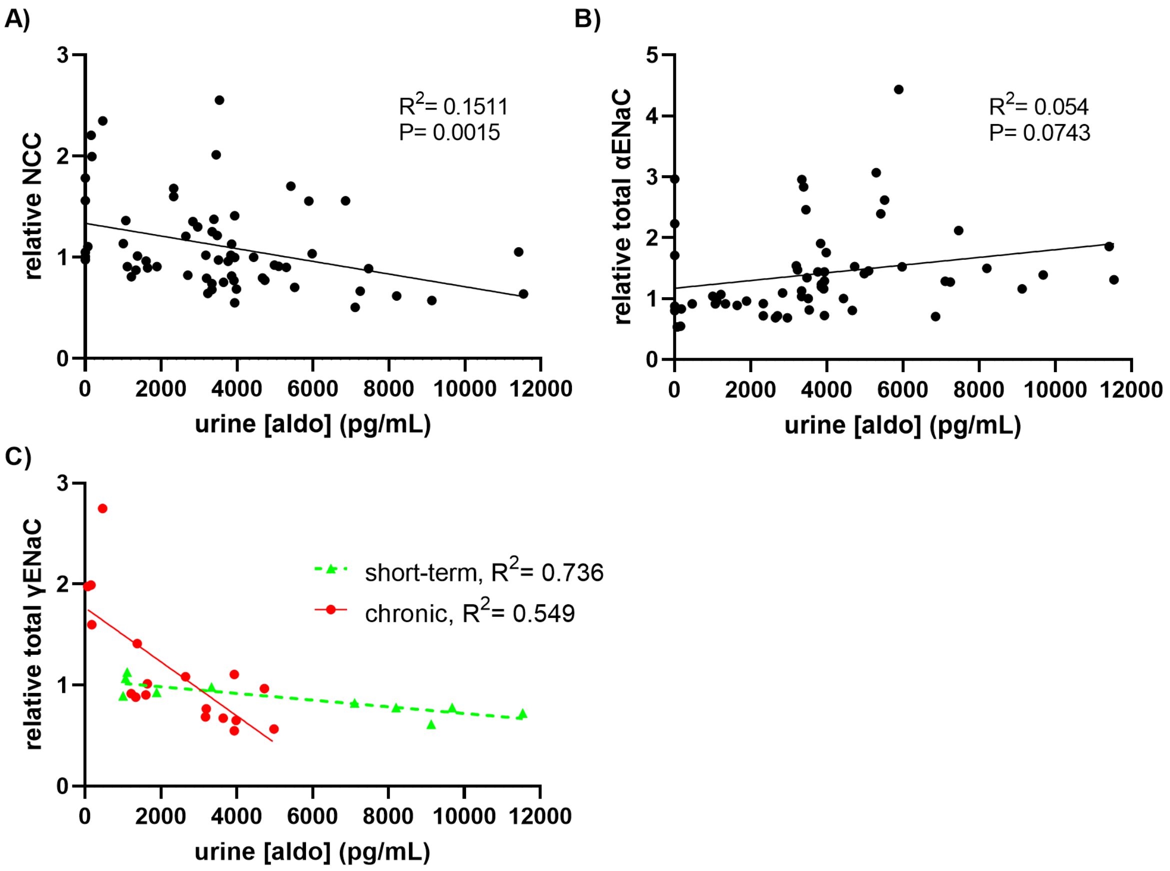

Supplemental Figure 11. Gene ontology (GO) analysis by ClueGO for proteins signficantly changed by short-term or chronic KCl feeding. Short-term feeding is 4 days, whereas chronic is 3 weeks. GO terms are based on a p<0.02 cut-off. A) GO terms overrepresented in the pool of proteins increased in abundance after short-term KCl feeding. B) GO terms signficantly overrepresented in the pool of proteins decreased in abundance after short-term KCl feeding. C) GO terms signficantly overrepresented in the pool of proteins increased in abundance after chronic KCl feeding. D) GO terms signficantly overrepresented in the pool of proteins decreased in abundance after chronic KCl feeding. The number of genes in each GO term group is shown in each GO term subgroup, with the percentage of genes associated with a particular biological function shown in the individual pie charts.

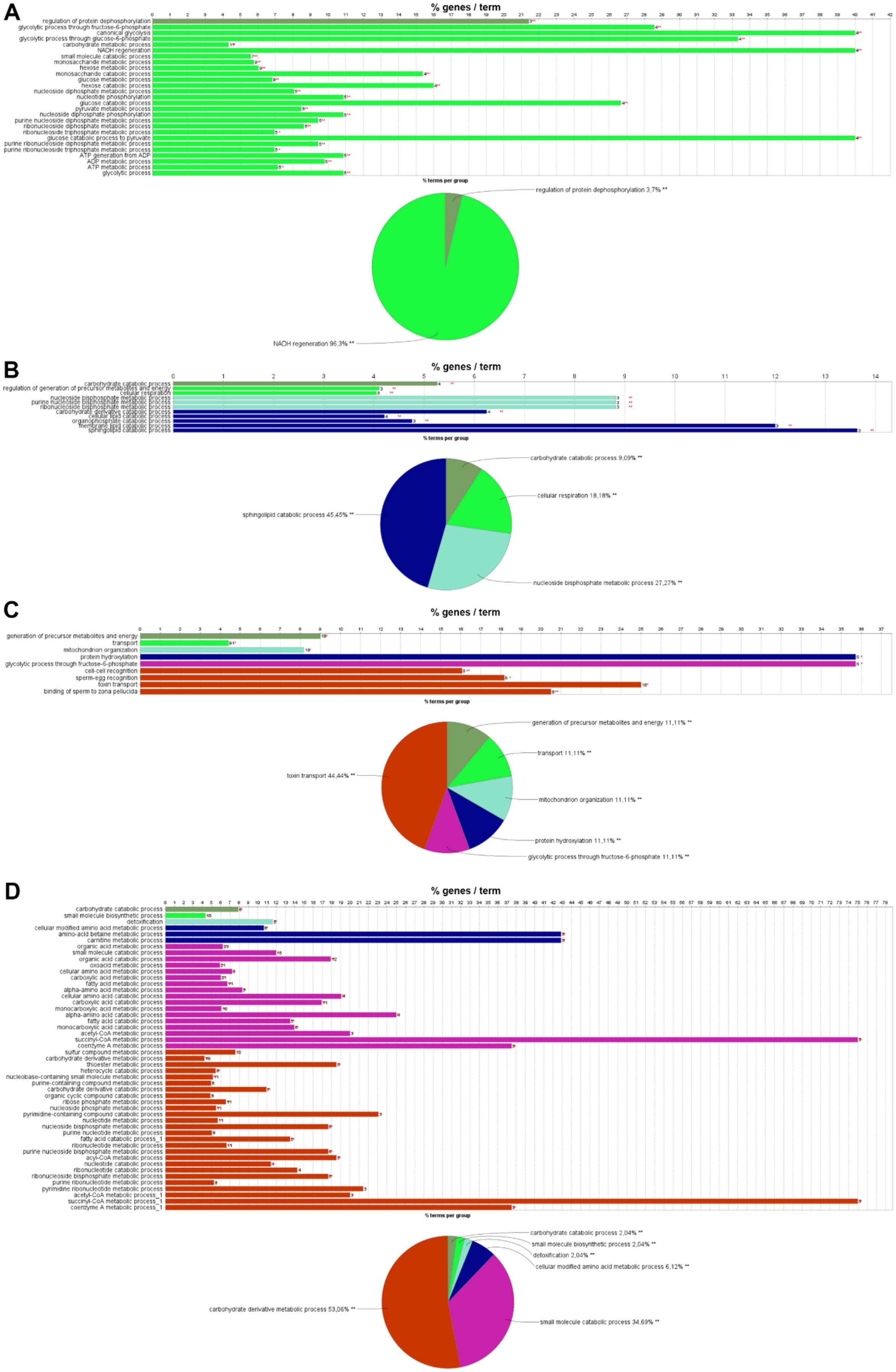
A
% genes / term
regulation of protein dephosphorylation
glycolytic process through fructose-6-phosphate
canonical glycolysis
glycolytic process through glucose-6-phosphate
carbohydrate metabolic process
NADH regeneration
small molecule catabolic process
monosaccharide metabolic process
hexose metabolic process
monosaccharide catabolic process
glucose metabolic process
hexose catabolic process
nucleoside diphosphate metabolic process
nucleotide phosphorylation
glucose catabolic process
pyruvate metabolic process
nucleoside diphosphate phosphorylation
purine nucleoside diphosphate metabolic process
ribonucleoside diphosphate metabolic process
ribonucleoside triphosphate metabolic process
glucose catabolic process to pyruvate
purine ribonucleoside diphosphate metabolic process
purine ribonucleoside triphosphate metabolic process
ATP generation from ADP
ADP metabolic process
ATP metabolic process
glycolytic process
% terms per group
regulation of protein dephosphorylation 3,7% **
NADH regeneration 96,3% **
B
% genes / term
carbohydrate catabolic process
regulation of generation of precursor metabolites and energy
cellular respiration
nucleoside bisphosphate metabolic process
purine nucleoside bisphosphate metabolic process
ribonucleoside bisphosphate metabolic process
carbohydrate derivative catabolic process
cellular lipid catabolic process
organophosphate catabolic process
membrane lipid catabolic process
sphingolipid catabolic process
% terms per group
carbohydrate catabolic process 9,09% **
cellular respiration 18,18% **
sphingolipid catabolic process 45,45% **
nucleoside bisphosphate metabolic process 27,27% **
C
% genes / term
generation of precursor metabolites and energy
transport
mitochondrion organization
protein hydroxylation
glycolytic process through fructose-6-phosphate
cell-cell recognition
sperm-egg recognition
toxin transport
binding of sperm to zona pellucida
% terms per group
generation of precursor metabolites and energy 11,11% **
transport 11,11% **
toxin transport 44,44% **
mitochondrion organization 11,11% **
protein hydroxylation 11,11% **
glycolytic process through fructose-6-phosphate 11,11% **
D
% genes / term
carbohydrate catabolic process
small molecule biosynthetic process
detoxification
cellular modified amino acid metabolic process
amino-acid betaine metabolic process
carnitine metabolic process
organic acid metabolic process
small molecule catabolic process
organic acid catabolic process
oxoacid metabolic process
cellular amino acid metabolic process
carboxylic acid metabolic process
fatty acid metabolic process
alpha-amino acid metabolic process
cellular amino acid catabolic process
carboxylic acid catabolic process
monocarboxylic acid metabolic process
alpha-amino acid catabolic process
fatty acid catabolic process
monocarboxylic acid catabolic process
acetyl-CoA metabolic process
succinyl-CoA metabolic process
coenzyme A metabolic process
sulfur compound metabolic process
carbohydrate derivative metabolic process
thioester metabolic process
heterocycle catabolic process
nucleobase-containing small molecule metabolic process
purine-containing compound metabolic process
carbohydrate derivative catabolic process
organic cyclic compound catabolic process
ribose phosphate metabolic process
nucleoside phosphate metabolic process
pyrimidine-containing compound catabolic process
nucleotide metabolic process
nucleoside bisphosphate metabolic process
purine nucleotide metabolic process
fatty acid catabolic process_1
ribonucleotide metabolic process
purine nucleoside bisphosphate metabolic process
acyl-CoA metabolic process
nucleotide catabolic process
ribonucleotide catabolic process
ribonucleoside bisphosphate metabolic process
purine ribonucleotide metabolic process
pyrimidine ribonucleotide metabolic process
acetyl-CoA metabolic process_1
succinyl-CoA metabolic process_1
coenzyme A metabolic process_1
% terms per group
carbohydrate catabolic process 2,04% **
small molecule biosynthetic process 2,04% **
detoxification 2,04% **
cellular modified amino acid metabolic process 6,12% **
carbohydrate derivative metabolic process 53,06% **
small molecule catabolic process 34,69% **

Supplemental Figure 12. Examples of proteins identified by mass spectrometry to have differential expression in the kidney cortex following high KCl feeding. Diet key: NS = normal NaCl (0.3% Na^+), NK = normal KCl (1.05% K^+), +KCl= high KCl (5.25% K^+). Short-term feeding is 4 days, whereas chronic is 3 weeks. Data is shown as mean ± SEM with individual data points representing different animals. Data compared using 1-way ANOVA with Tukeys multiple comparisons test.*P<0.05, **P<0.01, ***P<0.001, ****P<0.001.

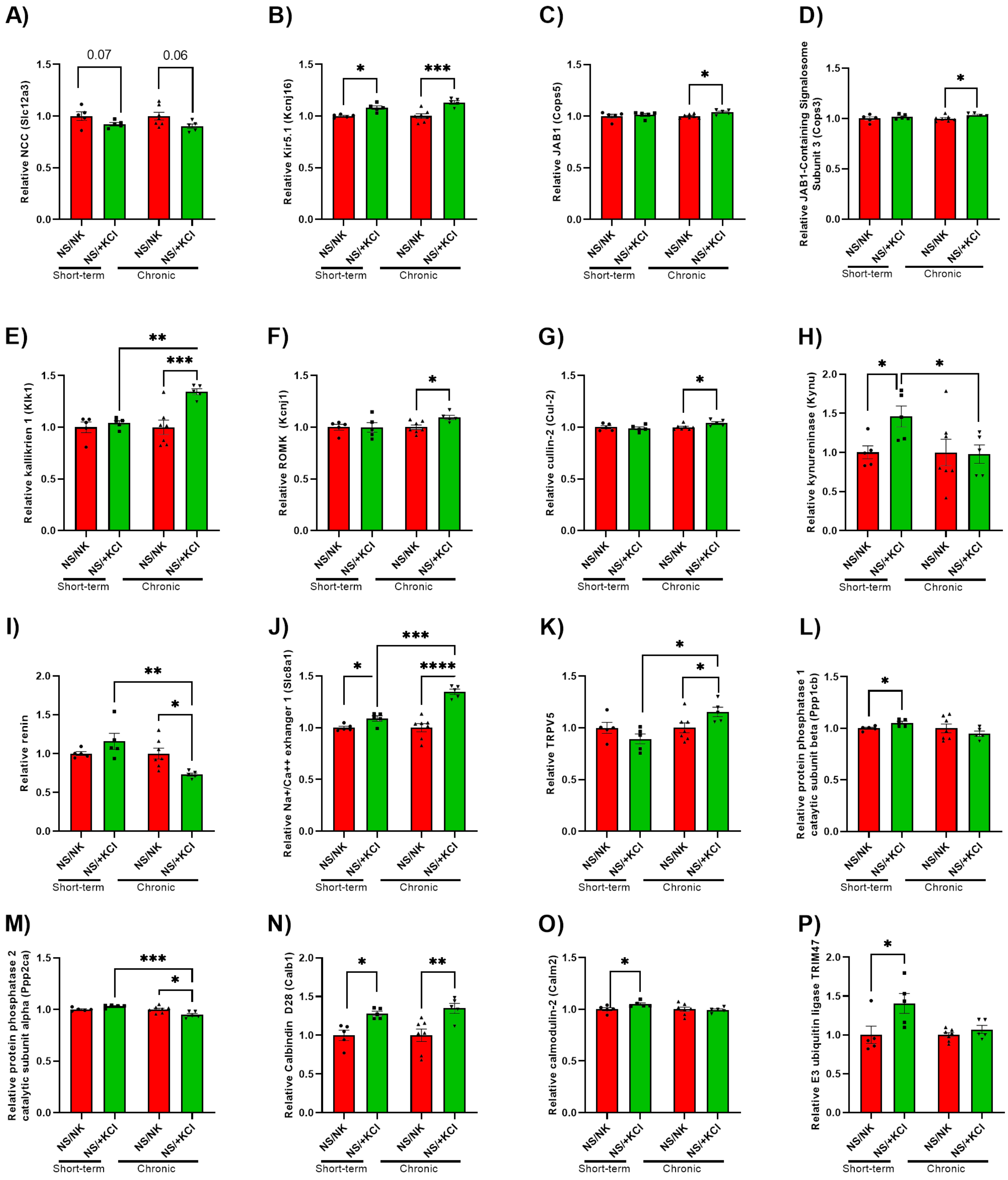

Supplemental table 4. Comparing chronic K^+ intake outcomes in current study to human population studies. Urinary excretion, as a surrogate marker of dietary intake, is reported for the end point of current study and following long term (> 6 weeks) dietary feeding regimes in humans. The chronic KCl feeding (5.25% K+ for 3 weeks) with normal NaCl content (0.3% Na+) was compared to salt subsitution studies in large human groups. The change (Δ) in ion excretion is calculated for the control arm and the intervention arm seperately. The relative change in excretion from intervention compared to control is then determined. For K+ excretion, the mice in the current study excreted ~1.8 to 2 fold more K+, indicating that the K+ intake in the current study was on the high end of what is easily achieveble in humans.

					URINARY EXCRETION													
study	reference	cohorts	summary of manipulation		CONTROL						Treatment: Fed or intervention						relative change	
					baseline		endpoint				baseline		endpoint				treatment Vs control at end	
				Parameter	mean	S.D	mean	S.D	Δ MEAN	relative Δ	mean	S.D	mean	S.D	Δ MEAN	relative Δ	absolute Δ	ratio Δ (1=no change)
Current study		wild type mouse feeding	NS/NK Vs NS/HK	Na (mg/d)	0.533	0.441	0.935	0.418	0.402	1.754	1.297	0.916	2.025	0.677	0.728	1.561	0.326	0.890
			3 weeks HK increases SBP 10 mmHg at night	K (mg/d)	5.987	4.912	9.536	2.892	3.548	1.593	14.542	11.554	62.240	19.945	47.698	4.280	44.150	2.687
				Na/K	88.782	16.354	94.790	17.993	6.008	1.068	92.859	15.043	32.722	4.910	-60.136	0.352	-66.144	0.330
Umeki et al 2021 Nutrients	PMID: 34684498	Japanese men lunches	approx 2X increase in KCl provided	Na (mg/d)	5035	1686	4784.00	1783.00	-251.000	0.950	4776.000	1728.000	4484.000	2019.000	-292.000	0.939	-41.000	0.988
		dietary replacemnet, HK with lower Na	6 week Higher K reduced SBP about 2.1mmHg	K (mg/d)	1898	582	1640.00	528.00	-258.000	0.864	1787.000	587.000	2222.000	804.000	435.000	1.243	693.000	1.439
				Na/K	2.8	1.1	3.10	1.30	0.300	1.107	2.800	1.200	2.100	0.900	-0.700	0.750	-1.000	0.677
Neal et al 2021 NEJM	PMID: 34459569	old Chinese villagers	25% increase in KCl as NaCl substitute	Na (mg/d)	4200	1800	4429.00		229.000	1.055	4400.000	1800.000	4039.000		-361.000	0.918	-590.000	0.870
		cooking salt replacement	SBP reduced by about 3.3 mmHg at 12 months	K (mg/d)	1400	600	1424.60		24.600	1.018	1400.000	600.000	2146.100		746.100	1.533	721.500	1.506
				Na/K	3		3.11		0.109	1.036	3.143		1.882		-1.261	0.599	-1.370	0.578
Yu et al 2021 Am J Clin Nutr	PMID: 33782684	rural India, aged with high BP	30% KCl: 70% NaCl Vs 100% NaCl	Na (mg/d)	3640	1730	2880.00	1980.00	-760.000	0.791	3800.000	1860.000	2900.000	1620.000	-900.000	0.763	-140.000	0.965
		cooking salt replacement	intervention significantly reduced SBP by 4.6mmHg	K (mg/d)	860	530	1130.00	1060.00	270.000	1.314	820.000	450.000	1420.000	1330.000	600.000	1.732	330.000	1.318
				Na/K	4.93	2.13	3.47	1.82	-1.460	0.704	5.360	2.870	2.700	1.580	-2.660	0.504	-1.200	0.716

REFERENCES for supplemental

1. Poulsen SB, Cheng L, Penton D, Kortenoeven MLA, Matchkov VV, Loffing J, et al. Activation of the kidney sodium chloride cotransporter by the beta2-adrenergic receptor agonist salbutamol increases blood pressure. *Kidney Int.* 2021.
2. Schepelmann M, Yarova PL, Lopez-Fernandez I, Davies TS, Brennan SC, Edwards PJ, et al. The vascular Ca2+-sensing receptor regulates blood vessel tone and blood pressure. *Am J Physiol Cell Physiol.* 2016;310(3):C193-204.
3. Ivy JR, Oosthuyzen W, Peltz TS, Howarth AR, Hunter RW, Dhaun N, et al. Glucocorticoids Induce Nondipping Blood Pressure by Activating the Thiazide-Sensitive Cotransporter. *Hypertension.* 2016;67(5):1029-37.
4. Little R, Zi M, Hammad SK, Nguyen L, Njegic A, Kurusamy S, et al. Reduced expression of PMCA1 is associated with increased blood pressure with age which is preceded by remodelling of resistance arteries. *Aging Cell.* 2017;16(5):1104-13.
5. Lewis S, Little R, Baudoin F, Prehar S, Neyses L, Cartwright EJ, et al. Acute inhibition of PMCA4, but not global ablation, reduces blood pressure and arterial contractility via a nNOS-dependent mechanism. *J Cell Mol Med.* 2018;22(2):861-72.
6. de Lannoy LM, Danser AH, van Kats JP, Schoemaker RG, Saxena PR, and Schalekamp MA. Renin-angiotensin system components in the interstitial fluid of the isolated perfused rat heart. Local production of angiotensin I. *Hypertension.* 1997;29(6):1240-51.
7. Christ-Crain M, and Fenske W. Copeptin in the diagnosis of vasopressin-dependent disorders of fluid homeostasis. *Nat Rev Endocrinol.* 2016;12(3):168-76.
8. Hunter RW, Ivy JR, Flatman PW, Kenyon CJ, Craigie E, Mullins LJ, et al. Hypertrophy in the Distal Convoluted Tubule of an 11beta-Hydroxysteroid Dehydrogenase Type 2 Knockout Model. *J Am Soc Nephrol.* 2015;26(7):1537-48.
9. Kortenoeven MLA, Cheng L, Wu Q, and Fenton RA. An in vivo protein landscape of the mouse DCT during high dietary K(+) or low dietary Na(+) intake. *Am J Physiol Renal Physiol.* 2021;320(5):F908-F21.
10. Wu Q, Poulsen SB, Murali SK, Grimm PR, Su XT, Delpire E, et al. Large-Scale Proteomic Assessment of Urinary Extracellular Vesicles Highlights Their Reliability in Reflecting Protein Changes in the Kidney. *J Am Soc Nephrol.* 2021;32(9):2195-209.
11. Benjamini Y, Drai D, Elmer G, Kafkafi N, and Golani I. Controlling the false discovery rate in behavior genetics research. *Behav Brain Res.* 2001;125(1-2):279-84.
12. Pedersen NB, Hofmeister MV, Rosenbaek LL, Nielsen J, and Fenton RA. Vasopressin induces phosphorylation of the thiazide-sensitive sodium chloride cotransporter in the distal convoluted tubule. *Kidney Int.* 2010;78(2):160-9.
13. Sorensen MV, Grossmann S, Roesinger M, Gresko N, Todkar AP, Barmettler G, et al. Rapid dephosphorylation of the renal sodium chloride cotransporter in response to oral potassium intake in mice. *Kidney Int.* 2013;83(5):811-24.
14. Masilamani S, Kim GH, Mitchell C, Wade JB, and Knepper MA. Aldosterone-mediated regulation of ENaC alpha, beta, and gamma subunit proteins in rat kidney. *J Clin Invest.* 1999;104(7):R19-23.
15. Christensen BM, Kim YH, Kwon TH, and Nielsen S. Lithium treatment induces a marked proliferation of primarily principal cells in rat kidney inner medullary collecting duct. *Am J Physiol Renal Physiol.* 2006;291(1):F39-48.
16. Cornelius RJ, Si J, Cuevas CA, Nelson JW, Gratreak BDK, Pardi R, et al. Renal COP9 Signalosome Deficiency Alters CUL3-KLHL3-WNK Signaling Pathway. *J Am Soc Nephrol.* 2018;29(11):2627-40.

17. Yang CL, Liu X, Paliege A, Zhu X, Bachmann S, Dawson DC, et al. WNK1 and WNK4 modulate CFTR activity. *Biochem Biophys Res Commun.* 2007;353(3):535-40.
18. Kim YH, Kwon TH, Frische S, Kim J, Tisher CC, Madsen KM, et al. Immunocytochemical localization of pendrin in intercalated cell subtypes in rat and mouse kidney. *Am J Physiol Renal Physiol.* 2002;283(4):F744-54.
19. Kim GH, Ecelbarger C, Knepper MA, and Packer RK. Regulation of thick ascending limb ion transporter abundance in response to altered acid/base intake. *J Am Soc Nephrol.* 1999;10(5):935-42.
20. Moeller HB, Aroankins TS, Slengerik-Hansen J, Pisitkun T, and Fenton RA. Phosphorylation and ubiquitylation are opposing processes that regulate endocytosis of the water channel aquaporin-2. *J Cell Sci.* 2014;127(Pt 14):3174-83.

Supplemental Data File 1

Dissociation of Sodium-Chloride Cotransporter Expression and Blood Pressure During Chronic High Dietary Potassium Supplementation

Robert Little[1], Sathish K. Murali[1*], Søren B. Poulsen[1*], Paul R. Grimm[2], Adrienne Assmus[1], Lei Cheng[1], Jessica R. Ivy[3], Ewout J. Hoorn[4], Vladimir Matchkov[1], Paul A. Welling[2] and Robert A. Fenton[1]

[1]Department of Biomedicine, Aarhus University, Aarhus, Denmark

[2]Departments of Medicine, Nephrology and Physiology, Johns Hopkins School of Medicine, Baltimore, USA

[3]University/BHF Centre for Cardiovascular Science, The Queen's Medical Research Institute, The University of Edinburgh, Edinburgh, United Kingdom

[4]Erasmus Medical Center, University Medical Center Rotterdam, Rotterdam, the Netherlands

<u>Description of supplemental Data file 1:</u>

File contains all uncropped and full length blots from main figures and supplemental figures

The boxed areas represent panels shown on main manuscript figures whereas quantification data is from all samples and multiple blots

Each raw blot image is directly comparable to the developed chemiluminescence image –so the Mw markers are comparable

Blue arrows represent specific bands that are quantified if non-specific bands are present

F3

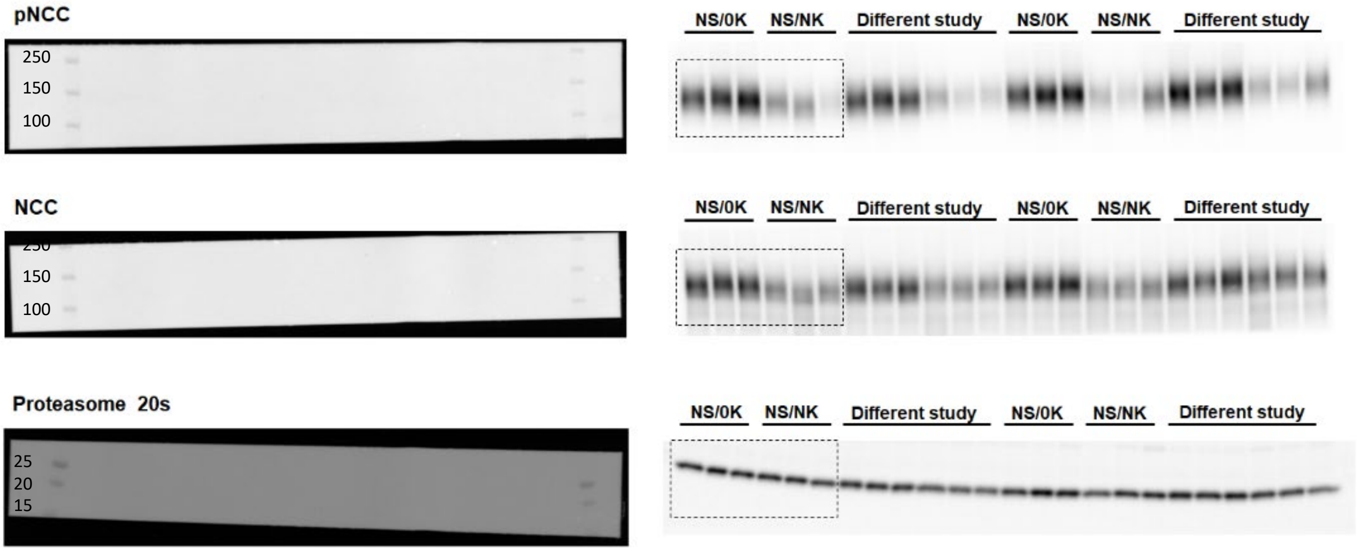

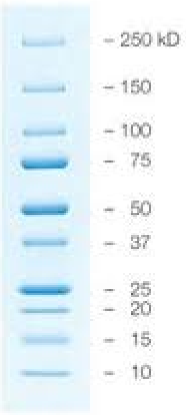

For all blots Precision Plus Protein ALL blue molecular weight standards are used

F5

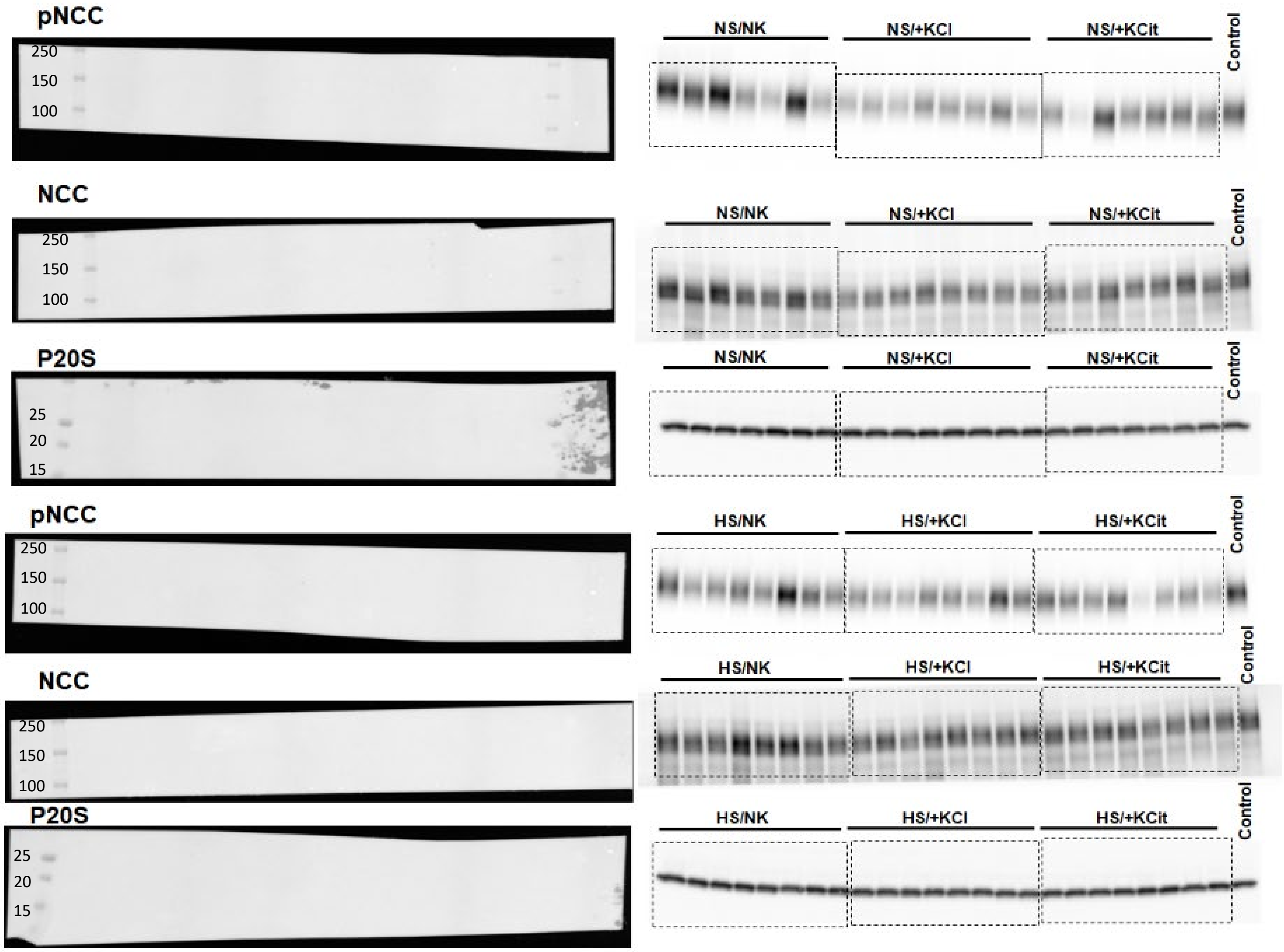

pNCC
250
150
100
NS/NK
NS/+KCl
NS/+KCit
Control
NCC
250
150
100
NS/NK
NS/+KCl
NS/+KCit
Control
P20S
25
20
15
NS/NK
NS/+KCl
NS/+KCit
Control
pNCC
250
150
100
HS/NK
HS/+KCl
HS/+KCit
Control
NCC
250
150
100
HS/NK
HS/+KCl
HS/+KCit
Control
P20S
25
20
15
HS/NK
HS/+KCl
HS/+KCit
Control

F6

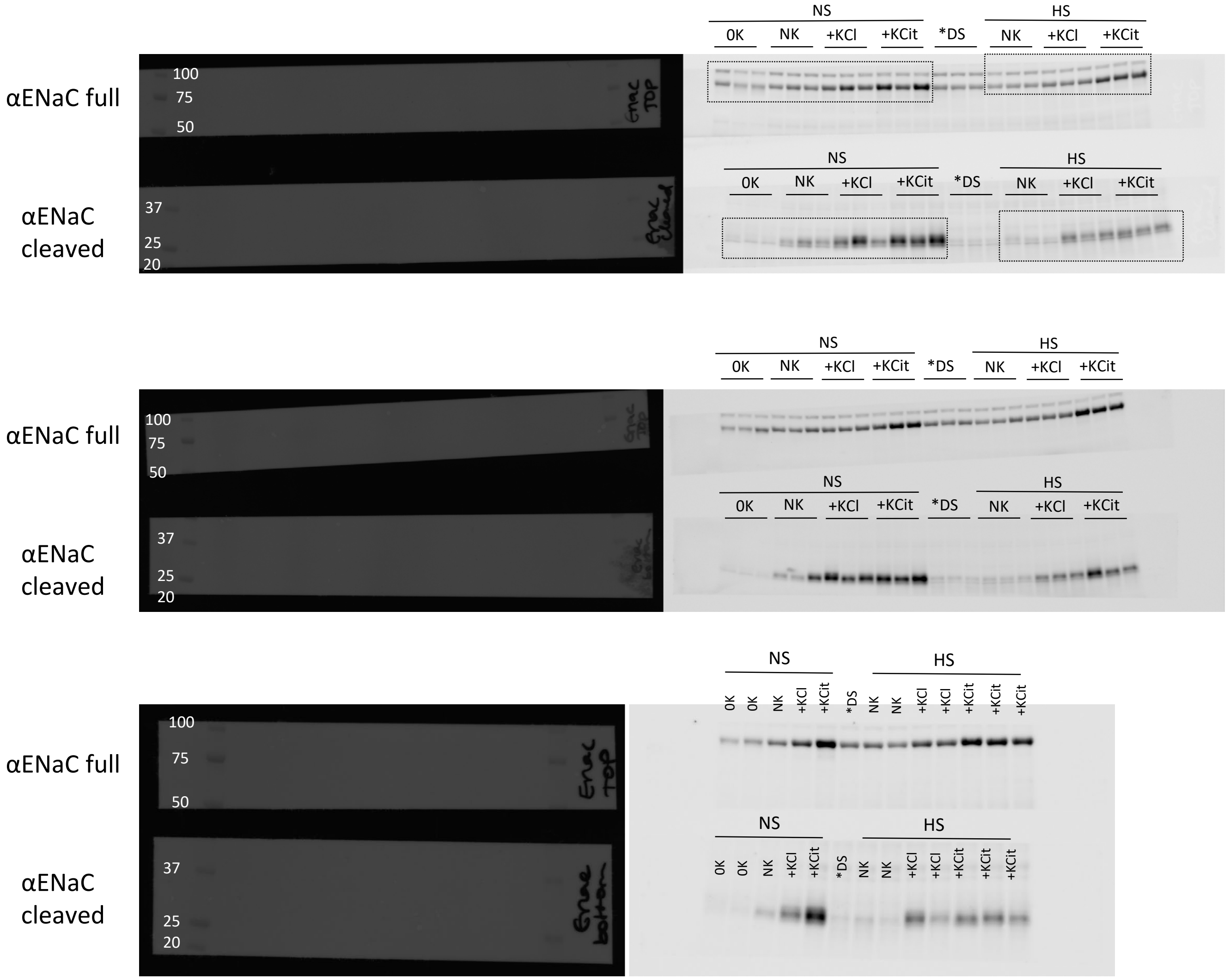
αENaC full
αENaC cleaved
NS
HS
0K
NK
+KCl
+KCit
*DS
100
75
50
37
25
20

F9

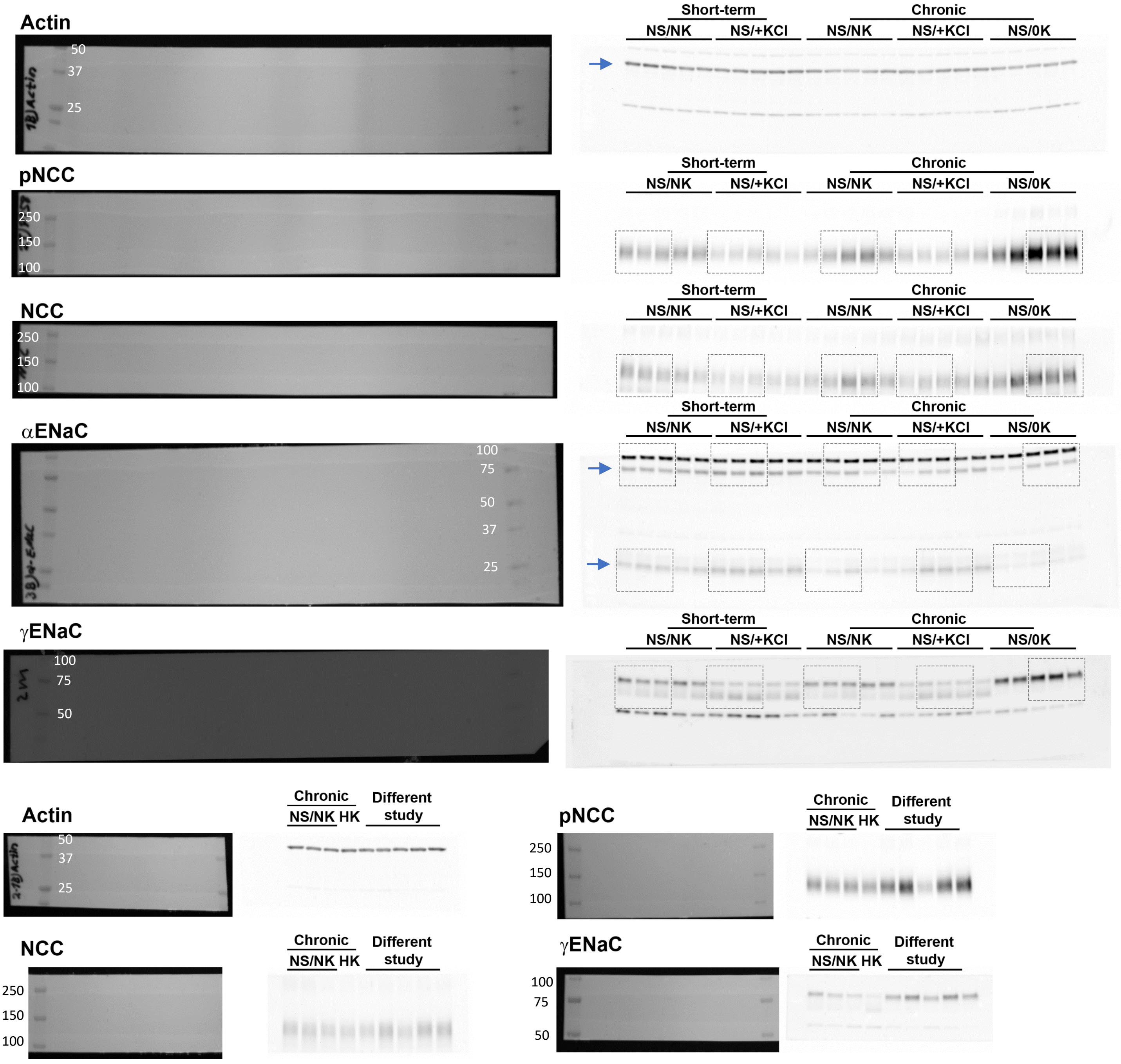
Actin
Short-term
Chronic
NS/NK
NS/+KCl
NS/NK
NS/+KCl
NS/0K
50
37
25
pNCC
250
150
100
NCC
αENaC
100
75
50
37
25
γENaC
Chronic
Different study
NS/NK HK

Calbindin D28

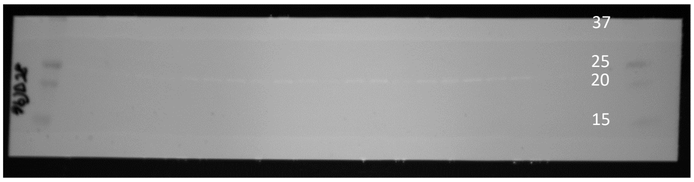

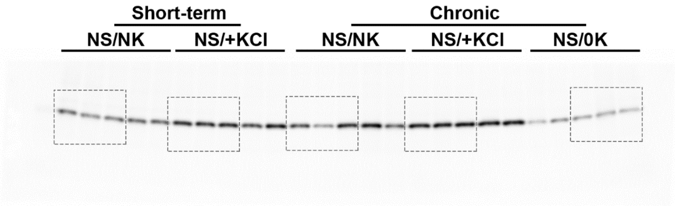

Parvalbumin

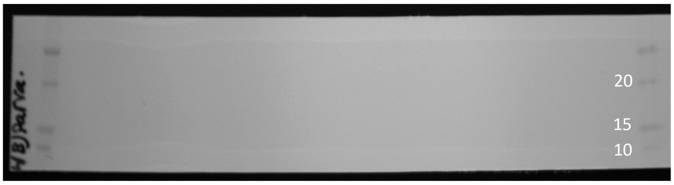

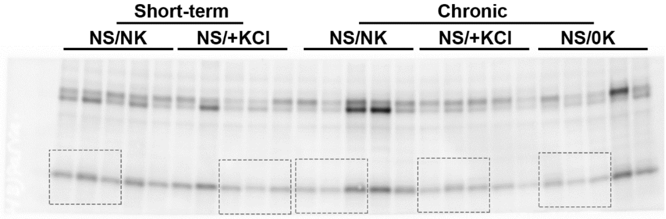

HATPase

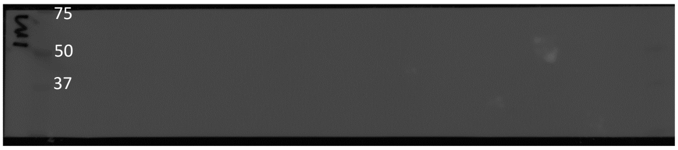

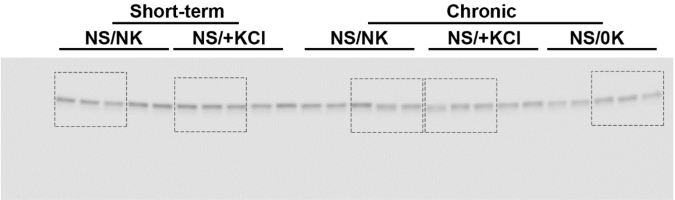

Calbindin D28

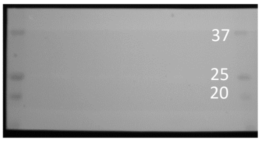

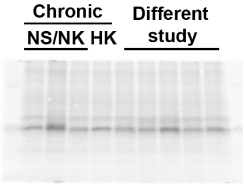

Parvalbumin

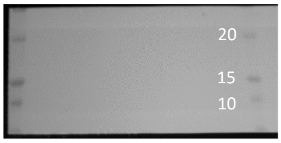

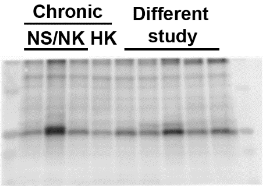

HATPase

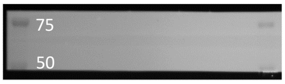

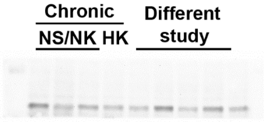

SF4

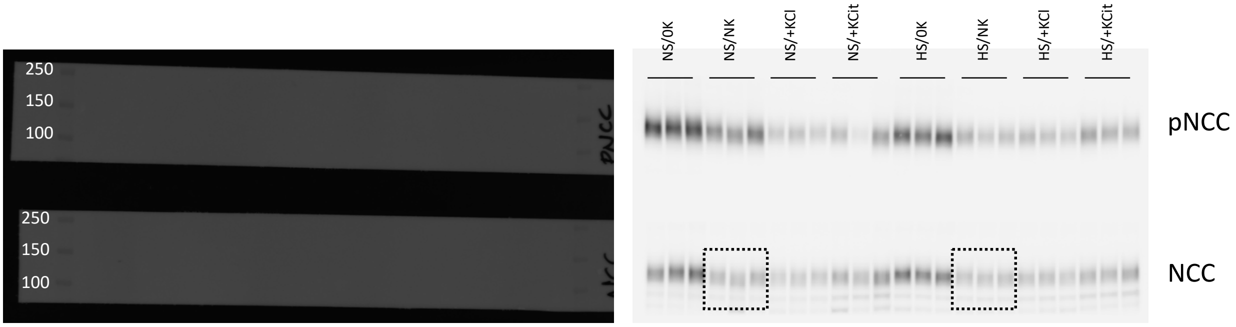
250
150
100
250
150
100
NS/0K
NS/NK
NS/+KCl
NS/+KCit
HS/0K
HS/NK
HS/+KCl
HS/+KCit
pNCC
NCC

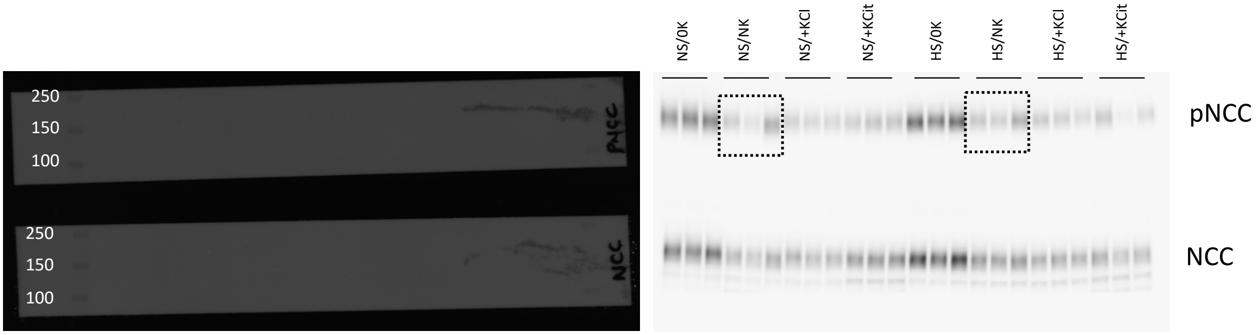
250
150
100
250
150
100
NS/0K
NS/NK
NS/+KCl
NS/+KCit
HS/0K
HS/NK
HS/+KCl
HS/+KCit
pNCC
NCC

SF8

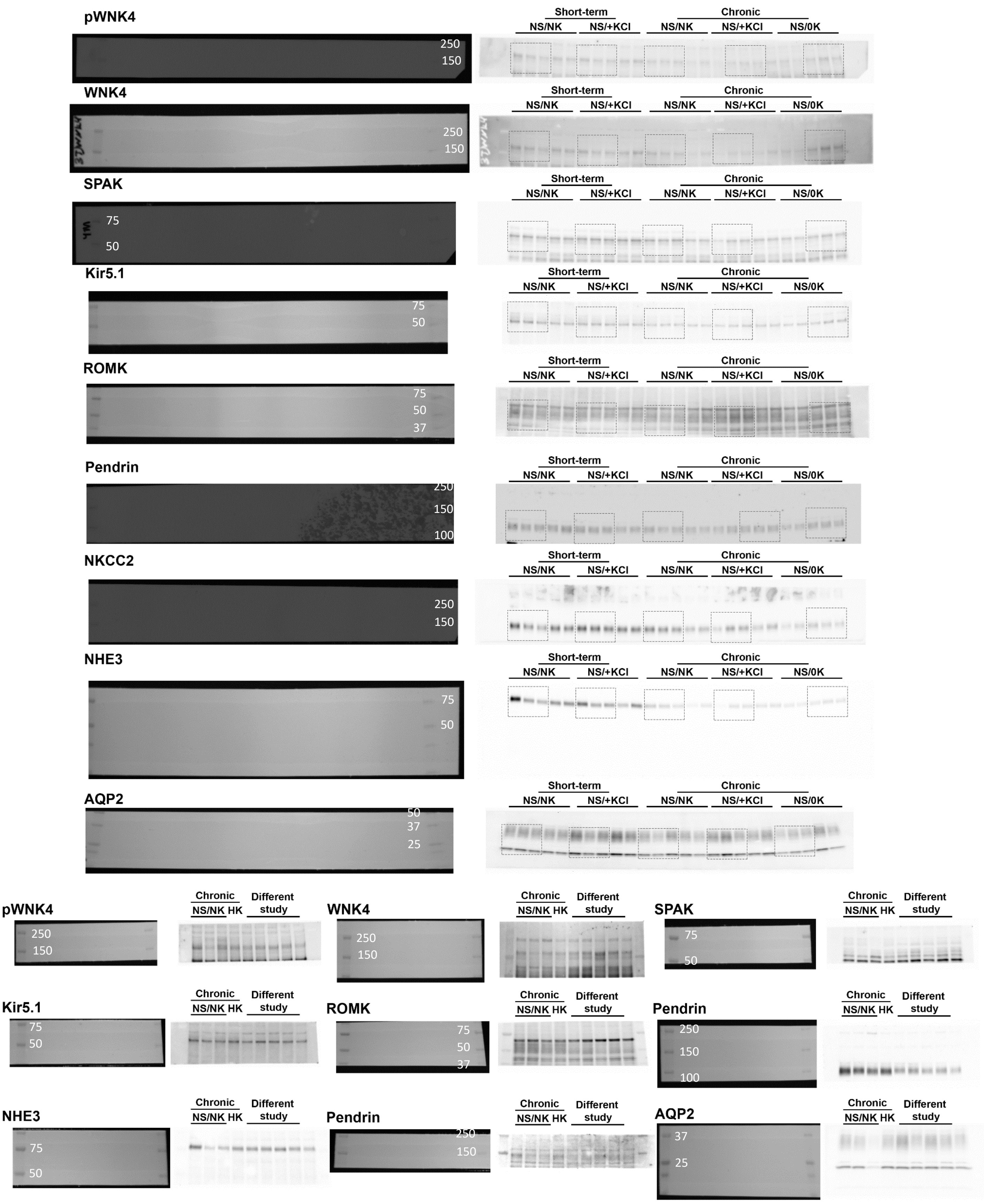
pWNK4
Short-term
Chronic
NS/NK
NS/+KCl
NS/NK
NS/+KCl
NS/0K
250
150
WNK4
Short-term
Chronic
NS/NK
NS/+KCl
NS/NK
NS/+KCl
NS/0K
250
150
SPAK
Short-term
Chronic
NS/NK
NS/+KCl
NS/NK
NS/+KCl
NS/0K
75
50
Kir5.1
Short-term
Chronic
NS/NK
NS/+KCl
NS/NK
NS/+KCl
NS/0K
75
50
ROMK
Short-term
Chronic
NS/NK
NS/+KCl
NS/NK
NS/+KCl
NS/0K
75
50
37
Pendrin
Short-term
Chronic
NS/NK
NS/+KCl
NS/NK
NS/+KCl
NS/0K
250
150
100
NKCC2
Short-term
Chronic
NS/NK
NS/+KCl
NS/NK
NS/+KCl
NS/0K
250
150
NHE3
Short-term
Chronic
NS/NK
NS/+KCl
NS/NK
NS/+KCl
NS/0K
75
50
AQP2
Short-term
Chronic
NS/NK
NS/+KCl
NS/NK
NS/+KCl
NS/0K
50
37
25
pWNK4
Chronic
NS/NK HK
Different study
250
150
WNK4
Chronic
NS/NK HK
Different study
250
150
SPAK
Chronic
NS/NK HK
Different study
75
50
Kir5.1
Chronic
NS/NK HK
Different study
75
50
ROMK
Chronic
NS/NK HK
Different study
75
50
37
Pendrin
Chronic
NS/NK HK
Different study
250
150
100
NHE3
Chronic
NS/NK HK
Different study
75
50
Pendrin
Chronic
NS/NK HK
Different study
250
150
AQP2
Chronic
NS/NK HK
Different study
37
25
